# Compound Augmentation of Myocardial Injury in a Rat Model of Coronary Heart Disease Induced by Ischemia/Reperfusion, Rheumatoid Arthritis, and High-Fat Diet: A Molecular Mechanistic Study

**DOI:** 10.3390/biom16050753

**Published:** 2026-05-21

**Authors:** Qixiang Xu, Jin Zhang, Lvming Li, Zhen Zhang, Zui Pan, Yongqiu Zheng

**Affiliations:** 1 Provincial Engineering Laboratory for Screening and Re-Evaluation of Active Compounds of Herbal Medicines in Southern Anhui, Wannan Medical University, Wuhu 241002, China; xuqixiang@wnmc.edu.cn (Q.X.); 20249139@stu.wnmc.edu.cn (L.L.); 20259157@stu.wnmc.edu.cn (Z.Z.); 2 Beijing Synchrotron Radiation Facility, Institute of High Energy Physics, Chinese Academy of Sciences, Beijing 100049, China; zhangjin2016@ihep.ac.cn; 3 Bone-Muscle Research Center, College of Nursing and Health Innovation, The University of Texas at Arlington, Arlington, TX 76019, USA

**Keywords:** rheumatoid arthritis, coronary heart disease, PTRF, TLR4/MyD88

## Abstract

Aims: Coronary heart disease (CHD) associated with rheumatoid arthritis (RA) is a primary driver of mortality in RA patients. In this study, we sought to establish a combined rat model of CHD and RA by integrating cardiac ischemia/reperfusion (I/R), high-fat diet (HFD), and intradermal administration of bovine type II collagen emulsified in complete Freund’s adjuvant. The aim of constructing this model is to investigate and analyze the pathogenesis of RA-induced CHD under the modulation of HFD and cardiac I/R exposure. Methods and Results: Sixty-four male Sprague–Dawley rats were randomly categorized into eight groups (*n* = 8 per group): control, I/R, HFD, collagen-induced arthritis (CIA), I/R + CIA, HFD + CIA, I/R + HFD, and I/R + HFD + CIA groups (*n* = 8 per group). We applied Synchrotron radiation-based X-ray micro-computed tomography (micro-CT) to observe the structural changes within the model over time. To further elucidate molecular mechanisms, transcriptome RNA-seq analysis was carried out to identify key signaling pathways, with particular emphasis on the homeostasis of Toll-like receptor 4 (TLR4)/Myd88 signaling in the ischemic myocardium. Furthermore, we conducted in vivo shRNA-mediated knockdown of polymerase I and transcription release factor (PTRF) and evaluated the co-localization of PTRF and TLR4 through immunofluorescence experiments. It is worth mentioning that our rat model of RA-induced (CHD) under a high-fat diet effectively manifested the relevant pathological features that align with the Traditional Chinese Medicine (TCM) definition of “bi” syndrome. The results indicate that the combined stimulation of HFD and CIA significantly elevated cardiac injury markers (CK-MB, LDH, CRP, and c-TNT) and was accompanied by a more severe expansion of the infarct area and increased cardiomyocyte apoptosis compared to the I/R group alone. In addition, the histopathological evaluation revealed significantly aggravated myocardial inflammation and fibrosis deposition, accompanied by extensive areas of tissue damage, further indicating a state of heightened inflammation and severe cardiac degenerative changes. Consistently, myocardial tissues from rats in the I/R + CIA + HFD group exhibited robust activation of the TLR4/MyD88 signaling pathway and a pronounced elevation in the p-JNK/JNK ratio. Moreover, pronounced co-localization between PTRF and TLR4 was evident in small vessels surrounding the infarcted myocardium. Importantly, AAV-mediated knockdown of PTRF attenuated the HFD- and CIA-induced exacerbation of myocardial injury in I/R rats. Conclusions: We successfully established a rat model of CHD with rheumatic syndrome using I/R in combination with RA and HFD. The present findings suggest that the PTRF-related TLR4/MyD88-JNK signaling pathway may act as an important regulatory mechanism underlying myocardial injury aggravated by combined HFD and CIA stimulation.

## 1. Introduction

Rheumatoid arthritis (RA) is a chronic autoimmune inflammatory disease that primarily affects the joints but also exerts systemic effects on multiple organs, particularly the cardiovascular system [1,2,3]. Patients with RA are at substantially increased risk of developing coronary heart disease (CHD), which represents a major cause of morbidity and mortality in this population [4,5,6]. This increased risk reflects the convergence of traditional cardiovascular risk factors with systemic inflammation and immune dysregulation inherent to RA. Chronic inflammation characteristic of RA drives endothelial dysfunction, accelerates atherosclerosis, and alters lipid metabolism [7,8,9], collectively promoting the development and progression of CHD. Despite the well-established RA-CHD association, current animal models fail to recapitulate the complex pathophysiological interactions between the two conditions, thereby limiting mechanistic insights and therapeutic evaluation.

From the perspective of Traditional Chinese Medicine (TCM), the coexistence of RA and CHD can be understood through the framework of the “Bi” syndrome, which encompasses systemic manifestations of inflammation and stagnation across multiple organ systems [10]. The rheumatic “Bi” syndrome, especially when accompanied by coronary microvascular disease (CMVD), is considered a predominant syndrome type in RA patients with CHD [11,12]. Developing an animal model that mimics this syndrome is therefore essential for unraveling the mechanisms by which RA exacerbates cardiovascular pathology and advancing integrated therapeutic strategies.

Collagen-induced arthritis (CIA) is a widely accepted model of RA that reproduces joint inflammation and systemic autoimmune responses, while a high-fat diet (HFD) induces dyslipidemia and atherogenesis, and cardiac ischemia/reperfusion (I/R) injury simulates acute ischemic events typical of CHD [13,14,15]. Collectively, these findings indicate that clinical myocardial ischemia is frequently triggered by multiple risk factors, including psychological stress, high-fat diet, and rheumatoid disorders, ultimately resulting in atherosclerosis and arterial luminal stenosis. Conventional animal models of myocardial ischemia, such as left anterior descending coronary artery ligation in rodents and porcine angioplasty models, cannot fully recapitulate the genuine pathogenesis and progressive pathological course of the clinical disease. In the present study, we established a novel rat model of atherosclerosis complicated with rheumatoid CHD induced by CIA. This model closely mimics the progressive pathophysiological process of the comorbid conditions, recapitulating key pathological features, including hyperlipidemia, atherosclerotic plaque deposition, endothelial injury, inflammatory activation, lipid accumulation, fibrous plaque formation, vascular luminal stenosis, and eventual myocardial ischemia. Furthermore, to nondestructively assess the micromorphological characteristics of rat tissues, synchrotron-based X-ray micro-CT was performed. This compound model with concurrent metabolic dysfunction offers a valuable tool to dissect interplay inflammation, autoimmunity, and cardiovascular injury and evaluate therapeutic strategies targeting both RA progression and cardiovascular risk.

Building on our prior work, we established the first rat model of HFD-induced non-alcoholic fatty liver disease (NAFLD) combined with RA. We found that CIA induced by CFA and bovine type II collagen aggravates HFD-related liver injury, and this pathological process is closely associated with the activation of the polymerase 1 and transcript release factor (PTRF/Cavin-1) signaling pathway [16].

Caveolae are specialized plasma membrane invaginations enriched in cholesterol, glycosphingolipids, and lipid-anchored proteins, and they are thought to play important roles in myocardial and coronary functions in CHD [17,18]. Structurally, caveolae are characterized by the presence of caveolin and cavin family proteins. The caveolin family comprises three isoforms: Cav-1 (caveolin-1), Cav-2, and Cav-3 [19,20], while the cavin family consists of four isoforms: PTRF/Cavin-1, serum deprivation protein response (SDPR/Cavin-2), SDR-related gene product that binds to C kinase (SRBC/Cavin-3), and muscle-restricted coiled-coil protein (MURC/Cavin-4) [21,22,23]. Caveolae are abundantly present in ventricular, atrial, and nodal cardiomyocytes [24,25]. Among these, PTRF/Cavin-1 is particularly enriched in the heart [21,26]. Furthermore, it has been reported that its role in mediating Toll-like receptor 4 (TLR4) internalization is crucial for regulating the intensity of the inflammatory signal in response to damage-associated molecular patterns (DAMPs) or lipopolysaccharide (LPS) [27]. Our previous study also demonstrated that PTRF/Cavin-1 acts as a key regulatory protein governing the formation of cellular caveolae and can sequester TLR4 in NAFLD combined with RA [16]. However, its precise role in regulating cardiac function remains controversial [17,26,28], and its contribution to CHD in the context of HFD and/or CIA is largely unknown.

In this study, we employed RNAi-mediated downregulation of PTRF in rats to investigate its role in I/R injury exacerbated by HFD and/or CIA. Elucidating this mechanism may provide new insights into the development of new integrative therapeutic strategies that combine conventional and traditional medicine approaches, ultimately aiming to improve outcomes in patients with RA-associated CHD.

## 2. Materials and Methods

### 2.1. Materials

Kits for the biochemical detection for lactate dehydrogenase (LDH, A020-2-2), creatine kinase MB (CK-MB, H197-1-2), C-reactive protein (CRP, H126-1-2), cardiac troponin T (cTNT, H149-4-2), total cholesterol (T-CHO, A111-1-1), triglycerides (TG, A110-1-1), aspartate transaminase (AST, C010-2-1) and alanine transaminase (ALT, C009-2-1) were purchased from Nanjing Jiancheng (Nanjing, China). Primary antibodies against TLR4 (Cat# 48-2300), Myd88 (Cat# PA5-19919), p-JNK (Cat# MA5-14943), JNK (Cat# AHO1362), GAPDH (Cat# MA5-15738-D800), and TRIzol Reagent (Cat# 15596026) were obtained from Thermo Fisher Scientific (Waltham, MA, USA).

The high-fat diet (XTHF45, Synergy Biotech Co., Ltd., Shanghai, China) provided 45% of energy from fat. The formulation contained (g per batch): casein 200.00, L-cystine 3.00, corn starch 72.80, maltodextrin 100.00, sucrose 176.80, cellulose 50.00, soybean oil 25.00, lard 177.50, mineral mix S10026B 50.00, vitamin mix V10001C 1.00, choline bitartrate 2.00, and FD&C Red Dye#40 0.05. The energy density was 4.70 kcal/g.

### 2.2. Animals

Specific pathogen-free (SPF) grade male Sprague-Dawley (SD) rats weighing 200 ± 20 g were obtained from SCXK (Henan) 2020-0005 (Department of Agriculture and Rural Affairs of Henan Province, Zhengzhou, Henan, China). All rats had free access to food and water and were maintained in a room at 22 ± 1 °C, with 65% ± 10% relative humidity and a 12 h light/dark cycle. All procedures were performed under general anesthesia to minimize animal suffering. During cardiac I/R model establishment, rats’ anesthesia was induced with 5% isoflurane ((Cat# 902-0000-522), EZVET Biotechnology Co., Ltd., Beijing, China) in 30% oxygen/70% medical air (1.5 L/min) and maintained on 2.5–3% isoflurane via a vaporizer and nose cone after loss of the righting reflex. Anesthetic depth was verified every 10 min by pedal withdrawal reflex, and body temperature was maintained at 37.0 ± 0.5 °C using a heating pad (Harvard Apparatus, Holliston, MA, USA). At the end of the protocol, euthanasia was performed under deep anesthesia by exsanguination followed by intracardiac injection of saturated potassium chloride (1 mmol/L, 1 mL/kg). Death was confirmed by apnea and absence of heartbeat for >5 min. The experiments were approved by the Animal Ethics Committee of the Wannan Medical College under the ethical code WNMC-AWE-2023479. All animal welfare and experimental procedures followed the regulations of the Animal Ethics Committee of Wannan Medical College.

### 2.3. Atherosclerosis and RA-Associated CHD Model Establishment

Sixty-four SD rats were randomly allocated to eight experimental groups, each comprising 8 rats. The treatment groups were as follows: control, cardiac I/R, HFD, CIA, cardiac I/R + CIA, CIA + HFD, cardiac I/R + HFD, and cardiac I/R+ HFD + CIA. Following a one-week acclimatization period, during which newly acquired rats were housed and fed, all groups, except the normal control, underwent experimental manipulations. The resulting models were complex and encompassed HFD, CIA, myocardial infarction, and their combinations.

Complete Freund’s Adjuvant (CFA) was prepared by suspending heat-killed Mycobacterium bovis Bacillus Calmette–Guerin (BCG) in liquid paraffin at a concentration of 10 mg/mL. A 0.1 mL emulsion containing 1 g/L of a 1:1 mixture of CFA and bovine type II collagen (Sigma–Aldrich, St. Louis, MO, USA) was administered via subcutaneous injection at the base of the tail to rats in the CIA, cardiac I/R + CIA, CIA + HFD, and cardiac I/R + HFD + CIA groups. A booster immunization with 0.1 mL of the emulsion was given on day 7 post-primary immunization, injected subcutaneously into the proximal one-third of the tail. Control group animals received an equal volume of saline administered at the same anatomical location. The day of the primary immunization was designated as day 0 (Figure 2a). On days 0, 5, 9, 13, 17, and 21 after immunization, the volume of the right hind paw was measured using an MK-550 plethysmometer (Muromachi Kikai Co., Ltd., Tokyo, Japan). Paw swelling (mL) was determined as the difference between the paw volume on each measurement day and the baseline volume recorded on day 0. Arthritis severity was quantitatively assessed using a standardized scoring system based on joint swelling and morphological changes. The rat paws were scored for arthritis as previously described [21]. Inflammation in the three non-injected paws was graded on a scale of 0 to 4 as follows: 0, no swelling or erythema; 1, mild swelling confined to toe joints; 2, moderate swelling of ankle or wrist joints; 3, pronounced swelling of the entire paw; and 4, severe inflammation accompanied by deformity or ankylosis. The scores from all three paws were summed, yielding a maximum possible arthritis score of 12 per animal. Day 0–day 21, rats in the HFD, CIA + HFD, cardiac I/R + HFD, and cardiac I/R + HFD + CIA groups were provided with a full daily ratio of atherogenic HFD, whereas the remaining groups were fed standard clean-grade chow.

The cardiac I/R model was established as described previously [29]. Briefly, rats anesthetized with 5% isoflurane underwent mechanical ventilation and a thoracic incision at the fourth intercostal space. The left anterior descending coronary artery was ligated with a silk suture for 30 min to induce ischemia. After suture release and chest closure, animals were transferred to a heating pad until full recovery from anesthesia. Sham-operated controls (including control, HFD, CIA, and CIA + HFD groups) received the same surgical procedure except for coronary artery ligation (Figure 2a).

### 2.4. Adeno-Associated Virus Serotype 9 (AAV9)-Driven In Vivo Gene Knockdown

For further knockdown experiments, the shPTRF sense oligonucleotide (5′-GCCAGATAAAGAAACTGGAGGTCAA-3′) and antisense oligonucleotide (5′-TTGACCTGGAGTTTCTTTATCTGGC-3′) were annealed and inserted into the GV594-U6-MCS-CAG-firefly_Luciferase vector. A non-targeting scrambled sequence was designed as the negative control. Subsequently, recombinant AAV9-luciferase vectors for PTRF knockdown and corresponding control vectors were successfully constructed.

Another 96 rats were randomly assigned to 16 groups with six animals per group: control group, AAV9 vectors carrying a specific shRNA targeting PTRF (AAV-PTRF-KD/AAV-anti-PTRF-KD) or a negative control (AAV-NC) were delivered via intravenous tail vein injection of 5 × 10^10^ AAV physical particles diluted in PBS (the procedure is shown in Figure 8a).

### 2.5. Biochemical Analysis

Serum concentrations of inflammatory cytokines, including IL-1β (EK301BEGB), IL-6 (EK306), TNF-α (EK382EGB), and VEGF (EK383), were quantified using commercial sandwich ELISA kits (MultiSciences Biotech, Hangzhou, China) according to the manufacturer’s protocols. The assay employed a quantitative sandwich immunoassay format with pre-coated capture antibodies, biotinylated detection antibodies, HRP-conjugated streptavidin, and TMB substrate. Absorbance was measured at 450 nm (reference wavelength: 570 or 630 nm), and cytokine concentrations were calculated using a four-parameter logistic standard curve. All samples were analyzed in duplicate.

Serum lipid profiles, including total cholesterol (T-CHO) and triglycerides (TG), were determined using enzymatic colorimetric kits (Nanjing Jiancheng Bioengineering Institute, Nanjing, China) based on the COD-PAP (Cat# A111-1-1) and GPO-PAP (Cat# A110-1-1) methods, respectively. Hepatic injury markers, alanine aminotransferase (ALT) and aspartate aminotransferase (AST), were measured using the Reitman–Frankel colorimetric assay (Cat# C009-2-1 for ALT; Cat# C010-2-1 for AST). This method detects the reaction of pyruvate (or oxaloacetate) with 2,4-dinitrophenylhydrazine to form a red-brown hydrazone complex, with absorbance read at 505 nm. Results were converted from Karmen units to U/L. Quality control was maintained in each batch using manufacturer-provided controls and standard curves; samples exceeding the linear range were diluted and re-assayed. All absorbance readings were performed on a Tecan Infinite Lumi multimode microplate reader (CH).

### 2.6. Measurement of Infarction Area

Upon completion of the reperfusion period, hearts were rapidly excised and rinsed with saline to remove residual blood. Myocardial tissue was sectioned into slices 0.1–0.2 cm thick, which were then incubated in 1% 2,3,5-triphenyltetrazolium chloride (TTC; Sigma Co., St. Louis, MO, USA) buffer (pH 7.4) at 37 °C in the dark to delineate the non-viable necrotic tissue within the area at risk. The stained sections were segmented and quantitatively analyzed using Image-Pro Plus 6.0 software. Infarct size, identified as white or pale unstained regions, was calculated as the percentage of infarcted myocardium relative to the total left ventricular area (infarct area/left ventricular area ×100%).

Based on the TTC staining results, we collected tissue samples from the myocardial ischemic penumbra (the region adjacent to the TTC-negative infarcted area) for subsequent histological examination, transcriptomic analysis, PCR, and Western blot assays.

### 2.7. Electrocardiogram

Electrocardiograms (ECGs) were used to identify the type of change (ST segment de-pression or elevation) after the experiment ended. The rats were anesthetized and ECGs were calculated as lead II ECGs using a VENTELITE (Harvard Apparatus Model 75-1500 Monitoring System, Holliston, MA 01746, USA). A stable period of at least 5 to 10 min after anesthesia induction but prior to any surgical intervention was recorded to establish the individual baseline for each rat. Subsequently, the type of change (ST segment elevation or depression) in the experimental rats was recorded and assessed by two researchers independently.

### 2.8. Histopathological Examination

For histopathological evaluation, standardized segments of cardiac, aortic, hepatic, and joint tissues were collected from all experimental animals. The myocardial ischemic penumbra adjacent to the TTC-negative infarcted area was dissected from the superior portion of the left ventricular lateral wall. All tissue specimens were fixed in 10% neutral buffered paraformaldehyde prepared in PBS. Joint samples were decalcified with EDTA for 10 days, after which all specimens were embedded in paraffin and serially sectioned at a thickness of 4 μm. Paraffin sections were stained with hematoxylin and eosin (HE), Masson’s trichrome, and terminal dUTP nick end-labeling (TUNEL). Histopathological observation and image acquisition were performed using an Olympus BX51 microscope system (Olympus Co. Ltd., Tokyo, Japan). Morphological alterations and structural lesions were quantified by ImageJ software (ImageJ (Fiji), Version 1.54, National Institutes of Health, Bethesda, MD, USA). All sections were independently examined and assessed by two blinded pathologists. The severity of inflammatory infiltration was evaluated using a classic semi-quantitative scoring system: grade 0, no inflammatory cell infiltration; grade 1 (mild), focal and sparse inflammatory infiltration with preserved tissue structure; grade 2 (moderate), diffuse loose infiltration or small focal inflammatory aggregates accompanied by slightly disorganized tissue architecture; grade 3 (severe), dense and diffuse inflammatory infiltration with cell aggregation, tissue structure destruction, or abscess formation.

### 2.9. TUNEL Staining of Heart Tissue

Paraffin sections of rat heart tissue were prepared and stained with a TUNEL Apoptosis Detection Kit (MilliporeSigma, Merck, Billerica, MA, USA) to detect apoptotic cells, strictly following the manufacturer’s instructions. The TUNEL assay was employed to determine the ratio of TUNEL-positive cells to the total number of cells. The sections were observed and evaluated by two independent pathologists to ensure objectivity. Apoptotic nuclei were visualized under a light microscope (Olympus BX51, Olympus Corporation, Tokyo, Japan), and the mean percentage of apoptotic cells was calculated thereafter.

### 2.10. Immunofluorescence

Paraffin-embedded heart tissue sections were subjected to immunofluorescence staining using the following primary antibodies: *TLR4* (1:50, Thermo Fisher Scientific, Waltham, MA, USA), CD31 (1:50, Abcam, Cambridge, UK), PTRF (1:50, Thermo Fisher Scientific, Waltham, MA, USA), and c-TnT (1:50, Proteintech, Rosemont, IL, USA). Corresponding secondary antibodies included FITC-conjugated goat anti-mouse IgG (1:500, Abcam, Cambridge, UK) and CY3-conjugated goat anti-rabbit IgG (1:500, Abcam, Cambridge, UK). Nuclei were counterstained with 4′,6-diamidino-2-phenylindole (DAPI). Fluorescence images were acquired via laser scanning confocal microscope (LSM 880, Carl Zeiss AG, Oberkochen, Germany).

### 2.11. Micro-Resolution X-Ray Tomography

**Figure 1:**
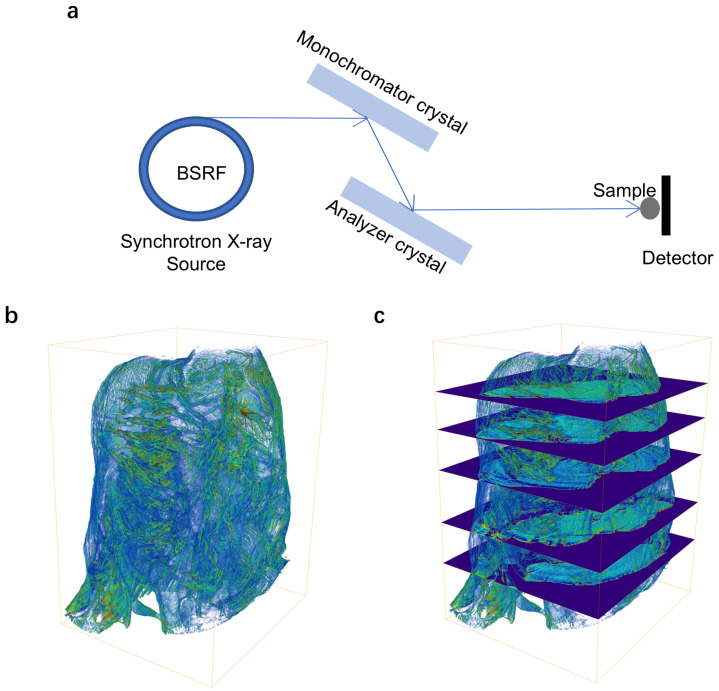
Synchrotron radiation-based micro-CT experimental setup and results of rat hearts. (**a**) Schematic diagram of the micro-resolution X-ray tomography setup at the beamline 4W1A. (**b**) 3D display of the heart of rats. (**c**) The schematic of five randomly selected slices.

To nondestructively investigate the micromorphological characteristics of rats, synchrotron-based X-ray micro-CT measurements were conducted at the 4W1A beamline of the Beijing Synchrotron Radiation Facility (BSRF). The configuration of the experimental apparatus is shown in Figure 1a. The rat was mounted on the sample stage and rotated by 180°. In total, 721 projection images were collected at 0.25° intervals from 0° to 180° at 12 keV [X-ray energy monochromatized by a Si (111) double crystal] with an exposure time of 2 s for each tomography scan. Subsequently, 3D tomographic reconstruction and data analysis were performed using MOCUPY (HEPSCT_MOCUPY V1.0, IHEP, Beijing, China), an in-house software developed by the HEPS TXM beamline team (http://www.ihep.cas.cn/dkxzz/HEPS/download/software/202305/t20230504_6748793.html) (accessed on 26 August 2025). Thereafter, 2D slicing, 3D rendering, and segmentation by threshold were performed using the Avizo software (Avizo v2024.2 , Thermo Fisher Scientific, Grand Island, NY, USA) for visualization. As shown in Figure 1b,c, a three-dimensional reconstruction volume was done and five slices were randomly selected from different regions of the sample. For quantitative analysis, threshold segmentation and binary were employed to differentiate between normal and damaged areas, with subsequent quantification of pixel counts in both the damaged area and the corresponding slice. The proportion of the damaged area in each slice relative to the entire sample slice was calculated by segmentation. The average of these proportions was calculated from the five slices, resulting in a column chart of the proportions of destroyed areas. The image reading/writing processes and associated statistical computations were implemented using custom-developed code in the MATLAB software (MATLAB 2023a, MathWorks, Natick, MA, USA) environment.

### 2.12. Transcriptome Expression Analysis by RNA Sequencing

Total RNA was extracted from rat myocardial samples with TRIzol reagent following the manufacturer’s protocol. Strand-specific RNA-seq libraries were constructed using the KC-Digital™ Stranded mRNA Library Prep Kit (Wuhan Seqhealth Co., Ltd., Wuhan, China), compatible with Illumina^®^ platforms. To identify differentially expressed genes (DEGs) between control and cardiac I/R + HFD + CIA groups, the edgeR package (Version 3.43.7, Bioconductor, Walter and Eliza Hall Institute of Medical Research, Parkville, Victoria, Australia) was employed with significance thresholds set at *p* < 0.05 and absolute fold change ≥2. Functional annotation and pathway enrichment analysis for DEGs were performed using KOBAS (v2.1.1), with Gene Ontology (GO) terms and Kyoto Encyclopedia of Genes and Genomes (KEGG) pathways considered significantly enriched at *p* < 0.05.

### 2.13. Validation of Gene Expression by qRT-PCR

Quantitative reverse transcription polymerase chain reaction (qRT-PCR) was conducted to verify the RNA-seq results for selected genes. Following total RNA extraction as previously outlined, cDNA was synthesized according to a standard reverse transcription protocol. The qPCR reaction mixture contained cDNA templates, specific primers (Table 1), SYBR Green master mix, and PCR buffer. Amplification was performed on a real-time PCR instrument with the following cycling parameters: an initial denaturation step at 95 °C, followed by 40 amplification cycles. The 2Δ^−ΔΔCt^ method was used to calculate relative gene expression, normalizing to β-actin as an internal control.

### 2.14. Western Blotting

Cardiac tissue specimens were homogenized in commercial RIPA lysis buffer, and total protein concentration was quantified using a bicinchoninic acid (BCA) assay. Protein samples were separated via SDS-PAGE and subsequently transferred onto PVDF membranes. After blocking at room temperature and washing with TBST, the membranes were incubated overnight at 4 °C with the following primary antibodies: *TLR4* (1:1500), Myd88 (1:1000), p-JNK (1:1000), and JNK (1:2000). This was followed by incubation with an appropriate horseradish peroxidase (HRP)-conjugated secondary antibody at room temperature. Immunoreactive bands were visualized by enhanced chemiluminescence (ECL) using SuperSignal West Femto Maximum Sensitivity Substrate, and their band density was analyzed with Bio-Rad Image Lab software (version 6.0).

### 2.15. PTRF–TLR4 Molecular Docking Analysis

To investigate the direct interaction between *PTRF* and *TLR4*, protein–protein docking was performed. The 3D structures of *PTRF* and *TLR4* were obtained from the RCSB PDB database. For proteins without experimentally resolved structures, homology modeling was conducted using the SWISS-MODEL server with templates of over 30% sequence identity. Protein structure preprocessing was carried out in Discovery Studio 2021, including removal of water molecules and redundant ligands, addition of hydrogen atoms, optimization of amino acid side chains, and energy minimization with the CHARMM force field to relieve steric hindrance and optimize structural geometry. *PTRF* and *TLR4* were assigned as receptor and ligand, respectively, for rigid-body docking using ZDOCK 3.0.2. With a grid resolution of 1.2 Å, 2000 initial complex conformations were generated and ranked by the ZDOCK scoring function based on van der Waals, electrostatic and desolvation effects. Representative binding modes were clustered by RMSD, and the lowest-energy conformation was selected for further analysis.

The binding free energy of the optimal complex was calculated via the PRODIGY server (kcal/mol), and conformations with binding energy ≤−5.0 kcal/mol were defined as having stable binding affinity. Structural visualization and interfacial interaction analysis were performed using PyMOL v2.5 and Discovery Studio to evaluate key residues, hydrogen bonds and hydrophobic interactions. The lowest-energy conformation was used for the presentation in Figure 7h.

### 2.16. Statistical Analysis

All experimental data are expressed as mean ± standard deviation (SD) unless otherwise specified. Prior to statistical testing, the normality of data distribution was assessed using the Shapiro–Wilk test, and homogeneity of variances was evaluated by Levene’s test. For data conforming to normal distribution and equal variances, one-way analysis of variance (ANOVA) followed by Least Significant Difference (LSD) post-hoc test was employed to compare differences among multiple groups. When the assumption of homogeneity of variances was violated, Welch’s ANOVA with Games-Howell post-hoc correction was applied. For data that did not meet the normality assumption, the non-parametric Kruskal–Wallis H test was performed, followed by Dunn’s post-hoc test with Bonferroni correction for multiple comparisons. Categorical data were analyzed using the Chi-square test or Fisher’s exact test, as appropriate. All statistical analyses were conducted using IBM SPSS Statistics software (version 26.0; IBM Corp., Armonk, NY, USA) and GraphPad Prism (version 9.0; GraphPad Software, San Diego, CA, USA). A two-tailed *p* value < 0.05 was considered statistically significant. Sample size (*n* = 8 per group) was determined based on preliminary experimental data and power analysis (power ≥80%, α = 0.05) to ensure adequate detection of biologically relevant differences.

## 3. Results

### 3.1. CFA Injection Induced Secondary Inflammatory Reaction of CIA in Cardiac I/R or HFD Rats

**Figure 2:**
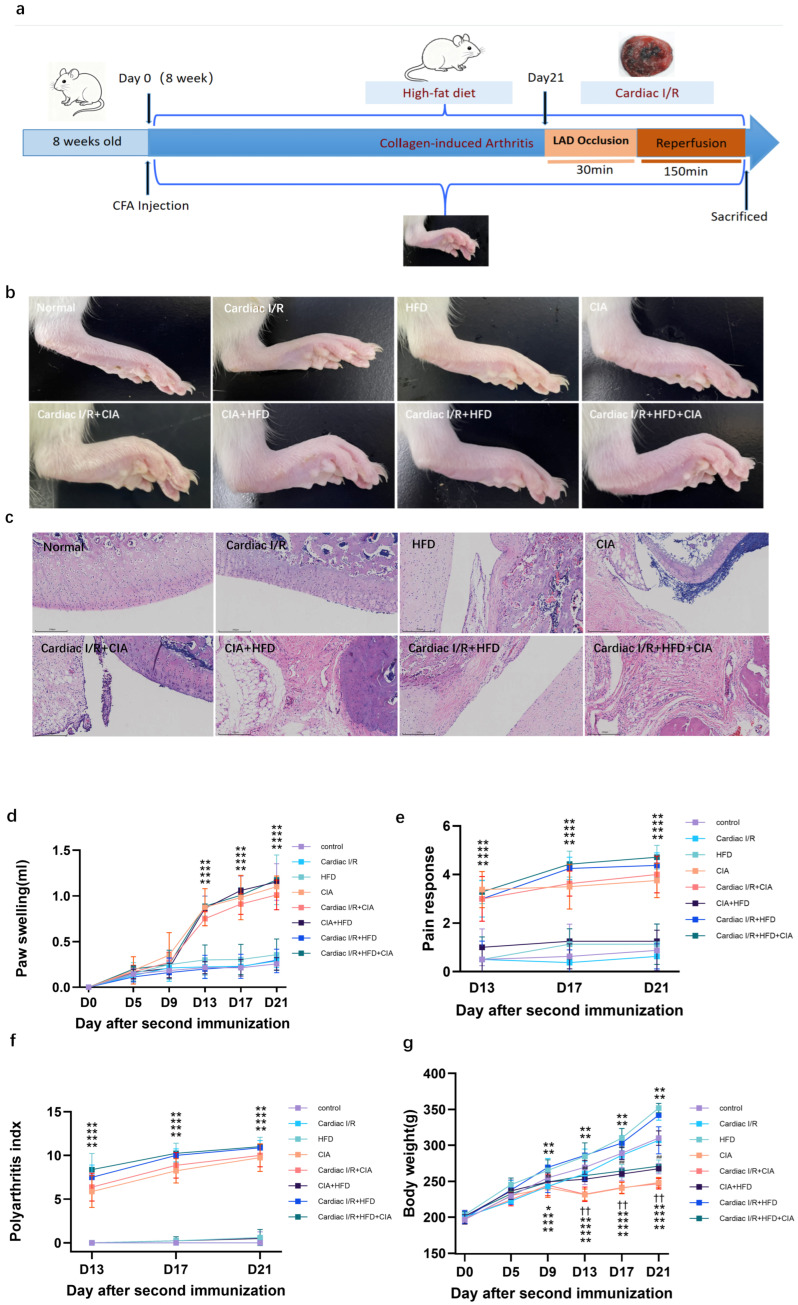
Paw swelling and histopathologic changes of the synovium in rats. (**a**) Experimental protocol. (**b**) Representative images of the swelling of the secondary paw of rats. (**c**) The histopathologic alterations of the synovial tissue in the rat. Scale bar = 250 µm (*n* = 8). (**d**) Degree of swelling of the non-injected hind paw (in mL) of CIA rats with or without HFD or cardiac I/R (*n* = 8). (**e**) Polyarthritis index in CIA rats with or without HFD or cardiac I/R (*n* = 8). (**f**) Pain response in CIA rats with or without HFD or cardiac I/R (*n* = 8). (**g**) Body weight changes in CIA rats with or without HFD or cardiac I/R (*n* = 8). Values are presented as mean ± SD, *n* = 8. * *p* < 0.05, ** *p* < 0.01 versus Control group, ^††^ VS cardiac I/R.

On day 9 after the initial immunization, rats developed swelling of the left hind paw, which reached its peak by day 21. By comparison, no significant swelling was observed in non-immunized rats. Secondary joint pathology was detected across groups. No pathological changes were noted in the control or cardiac I/R groups, while HFD induced mild joint lesions, with more pronounced damage in the HFD + I/R and HFD + CIA groups. CIA rats exhibited synovial proliferation, destruction of articular cartilage, and pannus formation with neovascularization. The most severe joint pathology occurred in the CIA + HFD and cardiac I/R + HFD + CIA groups, characterized by extensive synovial hyperplasia, pannus formation, and marked cartilage destruction. In addition, CFA reduced body weight in HFD-fed and cardiac I/R rats. However, HFD or cardiac I/R did not alter the arthritis index in CIA rats, including histopathological changes, paw swelling, pain response, and polyarthritis index (Figure 2). Collectively, this result demonstrates that the ischemia-reperfusion procedure itself exerts no influence on paw size and histological morphology, in contrast to the mild changes in CIA joint pathology caused by HFD.

### 3.2. HFD and CIA Exacerbate Arterial Inflammation, Liver Injury and Lipid-Inflammatory Abnormities

**Figure 3:**
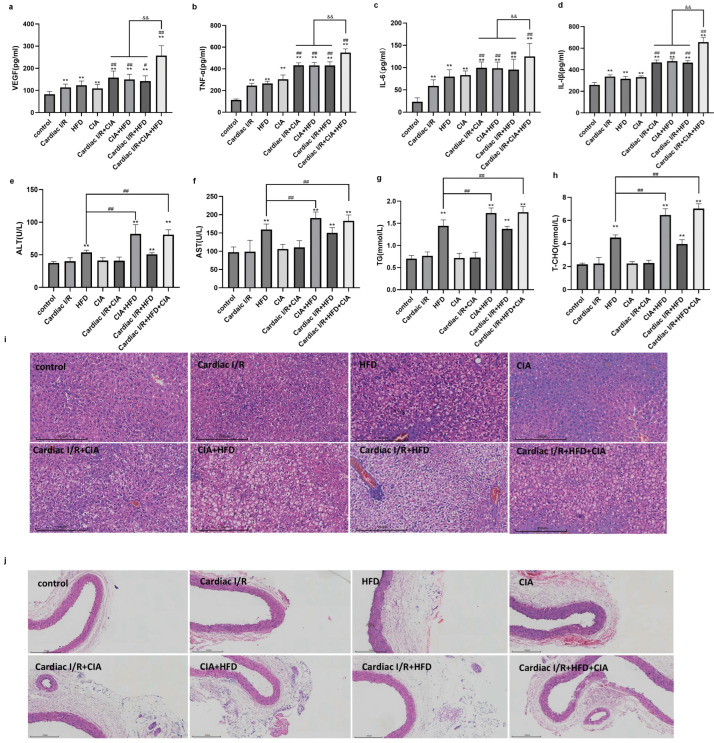
Expression of biomarkers related to lipid metabolism and liver function in the serum of rats in each experimental group. (**a**–**d**) Concentrations of serum VEGF, TNF-α, IL-6 and IL-1β were measured by the ELISA method. Data are presented as mean ± SD, *n* = 8. ** *p* < 0.01 vs. Control group. ^#^ *p* < 0.05, ^##^ *p* < 0.01 vs. cardiac I/R. ^&&^ *p* < 0.01 vs. cardiac I/R + HFD+CIA. (**e**–**h**) Levels of serum ALT, AST, TG and T-CHO (*n* = 8). Values are presented as mean ± SD. ** *p* < 0.01 vs. Control group. ^##^ *p* < 0.01 vs. HFD. (**i**) The representative pathological images of rat liver tissues. Scale bar = 250 µm. (**j**) The representative pathological images of rat abdominal aorta tissues. Scale bar = 250 µm.

We next evaluated circulating inflammatory mediators and lipid levels in each experimental group. Circulating levels of interleukin-1 beta (IL-1β), interleukin-6 (IL-6), tumor necrosis factor-alpha (TNF-α) and vascular endothelial growth factor (VEGF) were also elevated in cardiac I/R rats compared with controls. Moreover, both CIA and HFD markedly amplified these increases, with the highest levels observed in the cardiac I/R + HFD + CIA group (Figure 3a–d). Serum lipid profiles, including TG and T-CHO, were significantly elevated in all HFD-fed groups (HFD, CIA + HFD, I/R + HFD, and I/R + HFD + CIA) compared with the control group. These changes were accompanied by increased ALT and AST levels, indicating hepatic injury (*p* < 0.01). Notably, CIA further aggravated HFD-induced dyslipidemia in the CIA + HFD and cardiac I/R + HFD + CIA groups (*p* < 0.01) (Figure 3e–h).

As illustrated in Figure 3i, the liver tissue of rats in the control group exhibited a normal hepatic lobule structure, with neatly arranged hepatic cords and polygonal hepatocytes. No fibroplasia, inflammatory cell infiltration, or collagen accumulation was detected in this group. In the CIA group, mild fibroplasia, inflammatory cell infiltration, and collagen accumulation were observed; however, the hepatic lobule structure remained intact without obvious damage. In contrast, significant morphological abnormalities were found in the liver tissues of rats in the HFD, HFD + CIA, Cardiac I/R + HFD, and Cardiac I/R + HFD+ CIA groups. Specifically, a large number of lipid droplets accumulated in the cytoplasm, with the cell nucleus deviated to one side. The hepatic cords were disorganized, and the hepatocytes showed diffuse steatosis. Additionally, the hepatocytes were enlarged with loose cytoplasm, accompanied by prominent lipid droplet deposition. Notably, compared with the HFD group, the aforementioned morphological changes were significantly exacerbated in the Cardiac I/R + HFD+ CIA group, indicating a more severe liver injury and distinct lipid droplets in this group.

Furthermore, HE staining revealed that abdominal aortic tissues from the control and cardiac I/R groups maintained structural integrity, characterized by intact endothelial cells, orderly smooth muscle cell arrangement, and normal morphology of the adventitia. In contrast, the CIA + HFD and cardiac I/R + HFD + CIA groups exhibited marked vascular injury, including blurred vascular wall layers, extensive endothelial cell detachment, and disorganization with fibrous degeneration of smooth muscle cells. Compared with these groups, the HFD, CIA, cardiac I/R + CIA, and cardiac I/R + HFD groups largely preserved vascular integrity, with neatly arranged intima-media smooth muscle and reduced endothelial detachment and inflammatory infiltration (Figure 3i). Collectively, these findings suggest that the combined burden of CFA-induced inflammation and HFD-induced dyslipidemia exacerbates vascular injury, thereby increasing the risk of CVD, including coronary atherosclerotic events, heart failure, and other adverse cardiovascular outcomes (Figure 3j).

### 3.3. Administration of HFD and CIA Exacerbated Myocardial Injury in Cardiac I/R Rats

**Figure 4:**
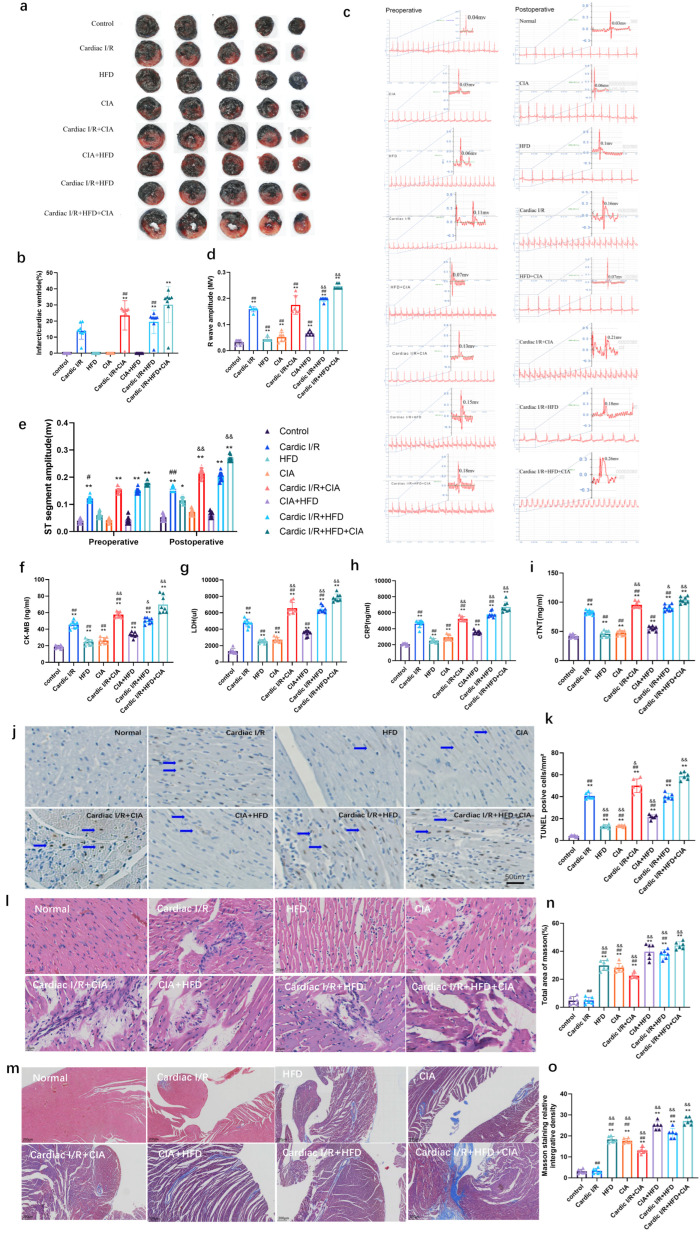
HFD and CIA treatments aggravated myocardial injury and myocardial apoptosis after cardiac I/R in vivo. (**a**) Representative heart tissue slices were subjected to cardiac I/R injury. Subsequently, 150 min after I/R, the heart was explanted, and local injuries to the left ventricle were shown in circled areas. TTC staining revealed increased infarct area following treatments with CFA or HFD. (**b**) Statistical data of infarct size in hearts subjected to I/R. Values are presented as mean ± SD, *n* = 8. ** *p* < 0.01 vs. Control group. ## *p* < 0.01 vs. cardiac I/R. (**c**) Comparison of electrocardiograms in rats of all groups. (**d**) Values of R wave amplitude of each group. Values are presented as mean ± SD, *n* = 8. * *p* < 0.05, ** *p* < 0.01 vs. control. && *p* < 0.01 vs. cardiac I/R. ## *p* < 0.01 vs. cardiac I/R + HFD + CIA. (**e**) Preoperative and Postoperative ST segment amplitude (mv). (**f**–**i**) Cardiac injury-related biomarkers in the serum of the rats in each group: serum CK-MB (**f**), LDH (**g**), CRP (**h**) and cTNT (**i**) levels as determined using respective kits. Data are presented as mean ± SD, *n* = 8. ** *p* < 0.01 vs. control. & *p* < 0.05, && *p* < 0.01 vs. cardiac I/R. ## *p* < 0.01 vs. cardiac I/R + HFD + CIA. (**j**) Myocardial apoptosis was analyzed using the TUNEL assay. Brown nuclei are TUNEL-positive apoptotic nuclei, indicated by blue arrows. Scale bar = 50 µm. (**k**) Summary of TUNEL staining shows that HFD or FCA treatments significantly increased apoptotic cardiomyocytes in infarct area (150 min after I/R injury). Data are presented as mean ± SD, *n* = 8. ** *p* < 0.01 vs. control. & *p* < 0.05, && *p* < 0.01 vs. cardiac I/R. ## *p* < 0.01 vs. cardiac I/R + HFD + CIA. (**l**) HE staining of myocardium in various groups of animals. Scale bar = 20 µm. (**m**) Masson staining of myocardium in each group of animals. Scale bar = 200 µm. (**n**,**o**) Summary of Masson staining shows that HFD or FCA treatments significantly increased both density and area of fibrosis in ischemic myocardium (150 min after I/R injury). Data are presented as mean ± SD, *n* = 8. ** *p* < 0.01 versus Control group. && *p* < 0.01 vs. cardiac I/R. ^#^ *p* < 0.05, ## *p* < 0.01 vs. cardiac I/R + HFD + CIA.

We observed distinct morphological and functional changes in the hearts of rats across the experimental groups. Myocardial sections revealed no infarcted regions in the control, HFD, CIA, and HFD + CIA groups. By contrast, the infarcted area, expressed as a percentage of the ventricular area, was significantly increased in the cardiac I/R, cardiac I/R + CIA, cardiac I/R + HFD, and cardiac I/R + HFD + CIA groups compared with controls, with the largest infarct size observed in the cardiac I/R + HFD + CIA groups (Figure 4a,b).

Electrocardiography showed elevated heart rates and ST segment elevations in the cardiac I/R, cardiac I/R + CIA, cardiac I/R + HFD, and cardiac I/R + HFD + CIA groups compared with the control group, with the most pronounced changes again in the cardiac I/R + HFD + CIA groups. No significant alterations were detected in the HFD, CIA, and HFD+CIA groups (Figure 4c,d).

Serum biomarkers of cardiac injury were consistent with these findings. Levels of CK-MB and LDH were significantly elevated in the cardiac I/R + HFD, cardiac I/R + CIA, and cardiac I/R + HFD + CIA groups compared with the cardiac I/R group, with the highest expression in the cardiac I/R + HFD + CIA group. Serum CRP levels were increased in all experimental groups except controls, with particularly high levels in the cardiac I/R + CIA, cardiac I/R + HFD, and cardiac I/R + HFD + CIA groups. The latter group also showed significantly greater CRP levels than the cardiac I/R + CIA and cardiac I/R + HFD groups. Similarly, serum cTNT levels were elevated in the cardiac I/R, CIA, cardiac I/R + CIA, cardiac I/R + HFD, and cardiac I/R + HFD + CIA groups, with the highest concentrations observed in the cardiac I/R + HFD + CIA group (Figure 4f–i).

Apoptosis analysis by TUNEL staining revealed only a few positive cells in the control, HFD, CIA, and HFD + CIA groups, but substantial numbers in the cardiac I/R, cardiac I/R + CIA, cardiac I/R + HFD, and cardiac I/R + HFD + CIA groups. The cardiac I/R + HFD + CIA group exhibited the greatest number of apoptotic cells (Figure 4j–k).

Histopathological analysis supported these findings. HE staining showed neat cardiomyocyte arrangement and intact structure in the control group, while the cardiac I/R, cardiac I/R + CIA, and cardiac I/R + HFD groups displayed disrupted, swollen, and disorganized myocardial fibers. The combination of cardiac I/R, HFD, and CIA led to extensive inflammatory infiltration and necrosis. In contrast, cardiomyocytes in the HFD, CIA, and HFD + CIA groups largely retained normal morphology with only minor inflammatory changes (Figure 4l and Table 2).

Masson’s trichrome staining further demonstrated significant myocardial fibrosis, with increased collagen deposition and blue-stained fibers in the HFD, CIA, HFD+CIA, and cardiac I/R + HFD+CIA groups, compared with minimal fibrosis in other groups (Figure 4m–o).

### 3.4. HFD and CIA Exacerbated Cardiac Damage and Collagen Deposition Assessed by Micro-Resolution X-Ray Tomography

**Figure 5:**
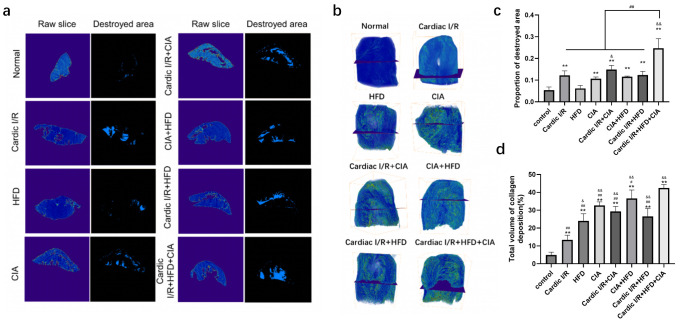
X-ray tomograms of the cardiac structure in each group of rats. (**a**) Raw and segmentation slice of the region of cardiac destruction in each group of rats (5×). (**b**) 3D volume visualization of selected slices combined with collagen deposition and cardiac destruction based on X-ray micro-CT. (**c**) Proportion of cardiac destruction area in rats. (**d**) Volume of collagen deposition. Data are presented as mean ± SD, *n* = 8. ** *p* < 0.01 vs. control group. ^&^ *p* < 0.05, ^&&^ *p* < 0.01 vs. cardiac I/R. ^#^ *p* < 0.05, ^##^ *p* < 0.01 vs. cardiac I/R + HFD + CIA.

Based on the TTC staining, three regions were identified in control hearts: the infarct area (TTC-negative), the border zone, and the remote area (healthy myocardium, TTC-positive). However, in rats subjected to cardiac I/R combined with HFD and/or CFA, extensive collagen deposition obscured the ischemic injury zone, complicating interpretation. To overcome this limitation, micro-resolution X-ray tomography was performed, providing high-resolution, multidimensional slices in a single nondestructive scan. Representative slices from both experimental and control groups are shown in Figure 5a. Cross-sectional spatial analyses revealed preserved cardiac structure in controls, whereas cardiac I/R and CIA treatments induced structural damage. The combination of HFD and CIA further exacerbated myocardial injury, with extensive collagen deposition compared to the cardiac I/R group alone (Figure 5b). Quantitative analysis, performed by threshold segmentation, binarization, and pixel-based statistical calculations across randomly selected slices, confirmed these observations and demonstrated a significantly greater proportion of damaged areas in the cardiac I/R + HFD + CIA group (Figure 5c).

### 3.5. Transcriptomic Analysis Revealed Altered Membrane Raft-Associated Processes in Cardiac I/R + HFD + CIA Animal Hearts

**Figure 6:**
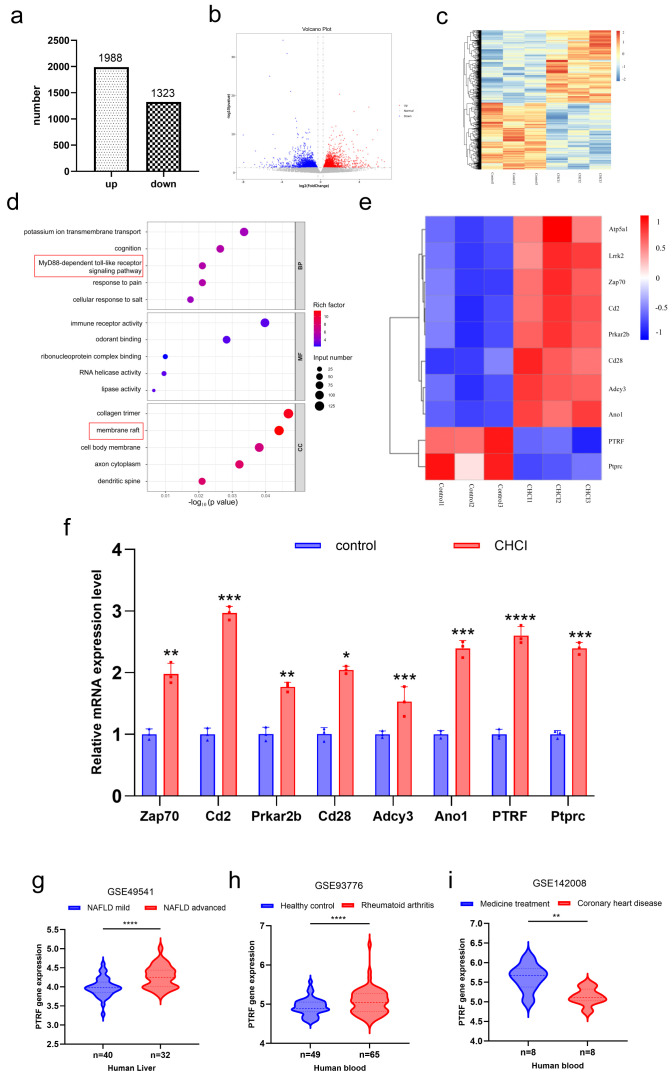
Transcriptomic analysis identifies PTRF as a key regulator of the membrane raft biological process in CHCI. (**a**) RNA sequencing revealed 2325 upregulated and 1387 downregulated genes in the CIA + HFD + Cardiac I/R (CHCI) group compared to controls. (**b**) Volcano plot highlighting differentially expressed genes (DEGs). (**c**) Heatmap illustrating global expression patterns of DEGs. (**d**) GO analysis showed significant enrichment in membrane raft organization and MyD88-dependent Toll-like receptor signaling pathways.The red box highlights the positions of the MYD88 pathway and membrane rafts within the top 15 most correlated genes. (**e**) Heatmap of DEGs associated with membrane raft-related processes. (**f**) RT-qPCR validation of eight upregulated membrane raft-related genes (*Adcy3*, *Ptprc*, *Ano1*, *PTRF*, *Prkar2b*, *Cd2*, *Cd28*, and *Zap70*), consistent with RNA-seq findings. (**g**–**i**) Expression profiles of PTRF in GEO datasets related to NAFLD (**g**), rheumatoid arthritis (RA) (**h**), and coronary heart disease (**i**). * *p* < 0.05; ** *p* < 0.01; *** *p* < 0.001; **** *p* < 0.0001.

Transcriptomic profiling was conducted by RNA sequencing (RNA-seq) of cardiac tissues from treated and control rats to investigate the molecular effects of combined I/R, HFD, and CIA treatments. In the I/R + HFD + CIA group, differential expression analysis revealed 2325 upregulated and 1387 downregulated genes compared with controls (fold change ≥2 or ≤−2; *p* < 0.05; Figure 6a–c). GO enrichment analysis indicated significant enrichment of DEGs in pathways related to “membrane raft organization” and the “MyD88-dependent Toll-like receptor signaling pathway” (Figure 6d).

Heatmap visualization (Figure 6e) demonstrated consistent upregulation of several membrane raft-associated genes, including *Adcy3*, *Ptprc*, *Ano1*, *PTRF*, *Prkar2b*, *Cd2*, *Cd28*, and *Zap70*, in the treated group. Validation by RT-qPCR confirmed the upregulation of these eight key genes (Figure 6f), with PTFR showing the most pronounced increase. Furthermore, integrative analysis of publicly available GEO datasets confirmed consistent upregulation of PTRF across three independent human disease cohorts (Figure 6g–i). Together, these data support the potential relevance of PTRF in the pathological response to combined cardiac I/R, HFD, and CIA stress.

### 3.6. HFD and CIA Activated PTRF-Related TLR4/MyD88-JNK Pathways in Ischemic Myocardium

**Figure 7:**
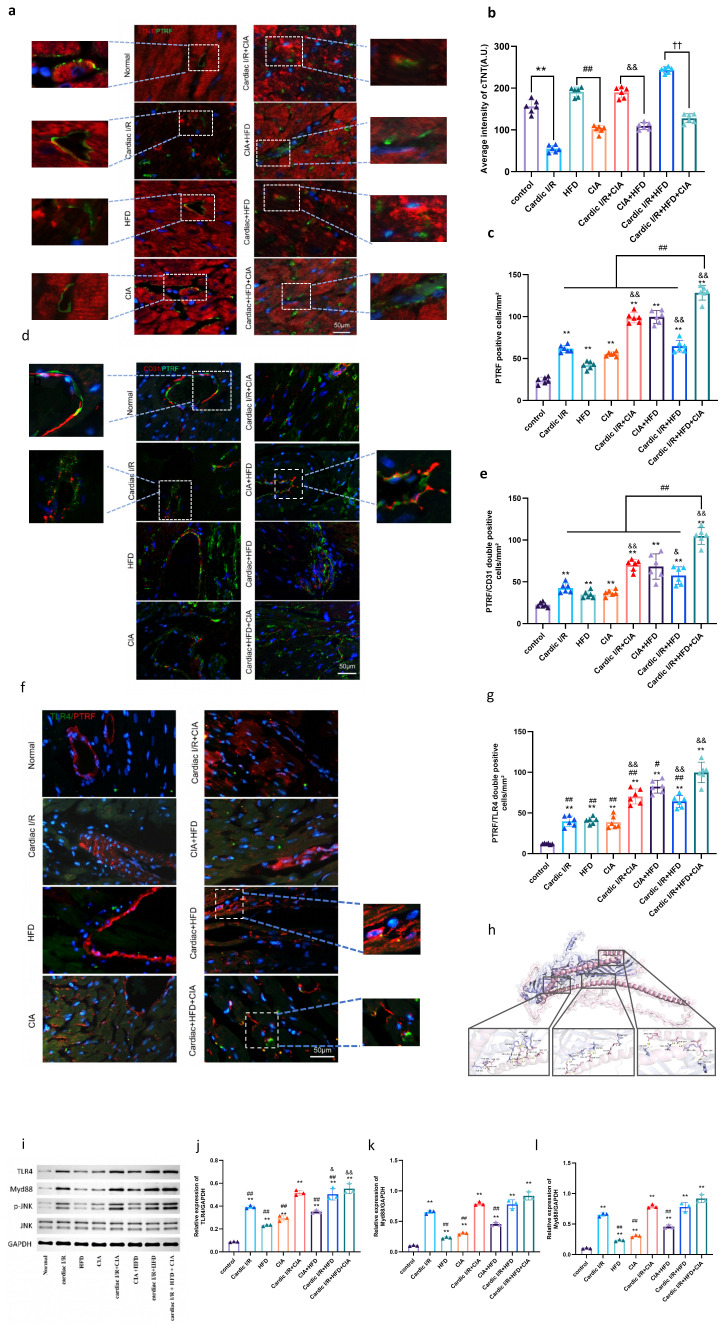
Expression of proteins associated with PTRF related-TLR4/Myd88- JNK pathways. (**a**) Images representing overlays of PTRF (green), cTNT (red), and nuclear staining by DAPI (blue). Scale bar = 50 μm. (**b**) Quantification of cTNT intensity. Data are presented as mean ± SD, *n* = 8. ** *p* < 0.01 vs. control, ^##^ *p* < 0.01 vs. HFD, ^&&^ *p* < 0.01 vs. CIA, ^††^ *p* < 0.01 vs. HFD + CIA. (**c**) Quantitative data of PTRF-positive cells. Data are presented as mean ± SD, *n* = 8. ** *p* < 0.01 vs. control. ^&&^ *p* < 0.01 vs. cardiac I/R. ^##^ *p* < 0.01 vs. cardiac I/R + HFD + CIA. (**d**) Merged confocal images representing overlays of PTRF (green), CD31 (red), and nuclear staining by DAPI (blue). Scale bar = 50 μm. (**e**) Quantitative data of PTRF and CD31 double-positive cells. Data are presented as mean ± SD, *n* = 8. ** *p* < 0.01 versus control; ^&^ *p* < 0.05, ^&&^ *p* < 0.01 vs. cardiac I/R; ^##^ *p* < 0.01 vs. cardiac I/R + HFD + CIA. (**f**) Co-localization of PTRF and TLR4 in merged confocal. Merged confocal images representing overlays of PTRF (red), TLR4 (green), and nuclear staining by DAPI (blue). Scale bar = 50 μm. (**g**) Quantitative data of PTRF and TLR4 double-positive cells. Data are presented as mean ± SD, *n* = 8. ** *p* < 0.01 versus control; ^&&^ *p* < 0.01 vs. cardiac I/R; ^#^ *p* < 0.05, ^##^ *p* < 0.01 vs. cardiac I/R + HFD + CIA. (**h**) Combination of the main compounds and protein targets. (**i**) Western blot analysis of the expression of the proteins TLR4, Myd88, p-JNK and JNK in the cardiac tissues of the rats. GAPDH was used as the internal control. (**j**) Densitometric analysis of TLR4/GAPDH. (**k**) Densitometric analysis of Myd88/GAPDH. (**l**) Representative quantitative analyses of JNK and p-JNK levels. Data are reported as mean ± SD (*n* = 3). ** *p* < 0.01 versus control; ^&^ *p* < 0.05, ^&&^ *p* < 0.01 vs. cardiac I/R; ^##^ *p* < 0.01 vs. cardiac I/R + HFD + CIA. Original images of (**i**) can be found in Supplementary Materials.

To elucidate the pathological mechanism by which HFD and CIA exacerbate myocardial injury, we first detected PTRF expression in myocardial tissues. Confocal microscopy revealed that PTRF was mainly distributed around small blood vessels, which was verified by its co-staining with the myocardial marker cTNT (Figure 7a,c) and the vascular marker CD31 (Figure 7d,e). Cardiac I/R induced obvious myocardial damage, and the number of PTRF-positive cells was significantly increased in the I/R, CIA, HFD, and I/R combined with HFD+CIA groups (Figure 7a–e).

To further explore the molecular interaction mechanism, protein-protein docking analysis was conducted. The results showed a stable binding conformation between PTRF and TLR4, with a minimum binding free energy of −6.8 kcal/mol (absolute value ≥5 kcal/mol), indicating a favorable binding affinity between the two (Figure 7h). These findings confirm a biologically relevant interaction between PTRF and TLR4.

Next, we examined the cellular localization of PTRF and TLR4 in ischemic myocardium (limited to I/R-treated groups). Strong colocalization of PTRF and TLR4 was observed in the I/R groups treated with HFD and/or CIA (Figure 7f,g). Western blot analysis further confirmed that the protein expression levels of TLR4 and MyD88 were significantly higher in the I/R group than in the control group, with more prominent increases in the I/R + CIA, I/R + HFD, and I/R + HFD + CIA groups(Figure 7i).

Finally, we evaluated the activation of the downstream signaling pathway by detecting the phosphorylation level of JNK. The ratio of phosphorylated JNK (p-JNK) to total JNK was significantly higher in the I/R group than in the control group, and this ratio was further elevated in the I/R + CIA, I/R + HFD, and I/R + HFD + CIA groups (Figure 7i–l). Collectively, these results indicate that HFD and CIA synergistically promote the activation of the PTRF-related TLR4/MyD88-JNK signaling pathway in ischemic myocardium.

### 3.7. AAV-Mediated PTRF Knockdown Attenuated HFD- and CIA- Exacerbated Myocardial Injury in Cardiac I/R Rats

**Figure 8:**
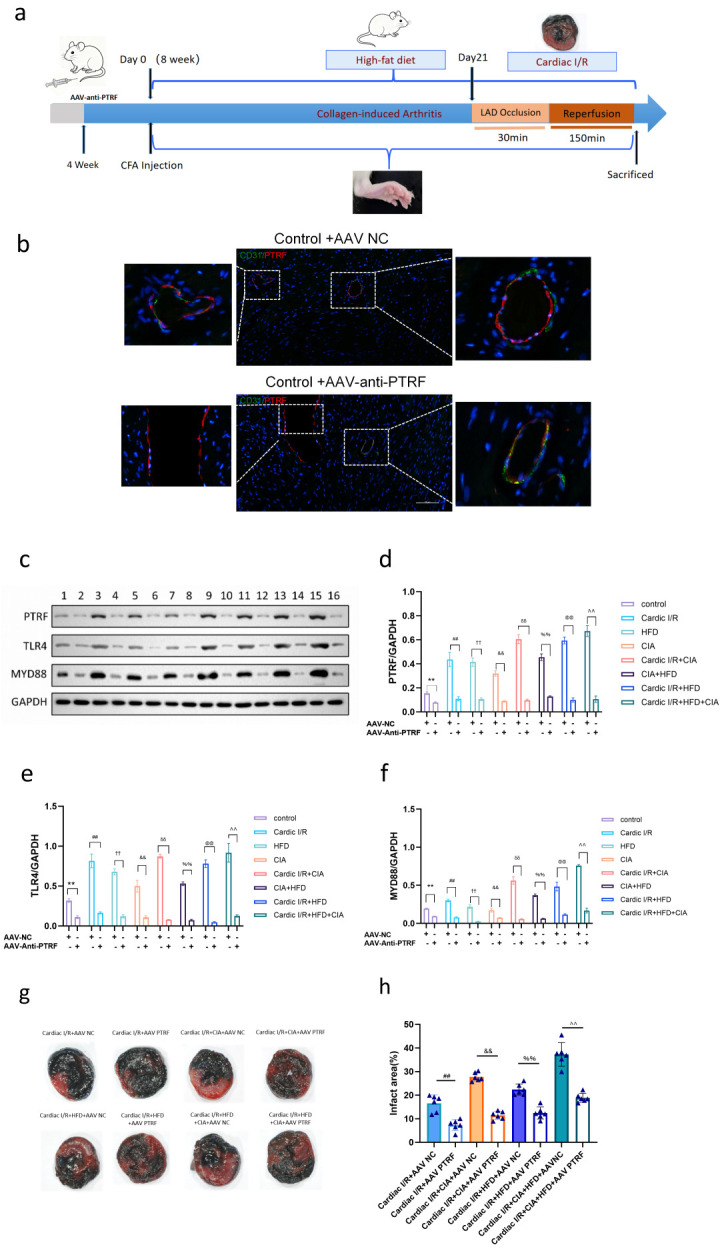
Cardioprotective effects of AAV-mediated PTRF knockdown in cardiac injury with HFD and/or CIA. (**a**) Experimental protocol. (**b**) Co-localization of PTRF and CD31 in merged confocal images representing overlays of PTRF (red), CD31 (green), and nuclear staining by DAPI (blue). Scale bar = 50 μm. (**c**) Western blot analysis of the expression of the proteins TLR4, Myd88, p-JNK and JNK in the cardiac tissues of the rats. GAPDH was used as the internal control. Results were obtained from three independent experiments; only one representative experiment is shown. (**d**) Densitometric analysis of PTRF/GAPDH. Data are reported as mean ± SD (*n* = 3). ** *p* < 0.01 versus control group; ^&&^ *p* < 0.01 vs. cardiac I/R + CIA; ^##^ *p* < 0.01 vs. cardiac I/R; ^††^ *p* < 0.01 vs. HFD; ^δδ^ *p* < 0.01 vs. CIA; ^%%^ *p* < 0.01 vs. CIA+HFD; ^@@^ *p* < 0.01 vs. cardiac I/R + HFD; ^^ *p* < 0.01 vs. cardiac I/R + HFD + CIA. (**e**) Densitometric analysis of TLR4/GAPDH. Data are reported as mean ± SD (*n* = 3). ** *p* < 0.01 vs. control; ^&&^ *p* < 0.01 vs. cardiac I/R + CIA; ^##^ *p* < 0.01 vs. cardiac I/R; ^††^ *p* < 0.01 vs. HFD; ^δδ^ *p* < 0.01 vs. CIA; ^%%^ *p* < 0.01 vs. CIA+HFD; ^@@^ *p* < 0.01 vs. cardiac I/R + HFD; ^^ *p* < 0.01 vs. cardiac I/R + HFD + CIA. (**f**) Densitometric analysis of Myd88/GAPDH. Data are reported as mean ± SD (*n* = 3). ** *p* < 0.01 vs. control; ^&&^ *p* < 0.01 vs. cardiac I/R + CIA; ^##^ *p* < 0.01 vs. cardiac I/R; ^††^ *p* < 0.01 vs. HFD; ^δδ^ *p* < 0.01 vs. CIA; ^%%^ *p* < 0.01 vs. CIA+HFD; ^@@^ *p* < 0.01 vs. cardiac I/R + HFD; ^^ *p* < 0.01 vs. cardiac I/R + HFD + CIA. (**g**) Representative heart slices from rats subjected to cardiac I/R injury. TTC staining revealed reduced infarct area following treatments with AAV-PTRF-KD vector. (**h**) Statistical data of infarct size in rat hearts subjected to I/R. Data are reported as mean ± SD (*n* = 6). ^##^ *p* < 0.01 vs. cardiac I/R + AAV NC; ^&&^ *p* < 0.01 vs. cardiac I/R + CIA + AAV NC; ^%%^ *p* < 0.01 vs. cardiac I/R + HFD + AAV NC; ^^ *p* < 0.01 vs. cardiac I/R + CIA + HFD + AAV NC. Original images of (**c**) can be found in Supplementary Materials.

Based on the above findings, we hypothesized that reducing PTRF expression would attenuate TLR4 activation and thereby alleviate cardiac injury induced by HFD and CIA in I/R rats. To test this, AAV9 vectors carrying a specific shRNA targeting PTRF (AAV-PTRF-KD) or a negative control (AAV-NC) were delivered via intravenous tail vein injection (Figure 8a). Confocal microscopy revealed that PTRF was significantly reduced around small vessels transfected with AAV-PTRF-KD compared with their respective AAV-NC controls (Figure 8b). Western blot analysis confirmed that PTRF expression was significantly reduced in all groups transfected with AAV-PTRF-KD compared with their respective AAV-NC controls (Figure 8c,d). As anticipated, knockdown of PTRF markedly decreased the expression of TLR4 and MyD88 proteins in the treated groups (Figure 8c,e–f), indicating that PTRF reduction could inhibit overactivation of the TLR4 pathway under conditions of HFD and/or CIA.

Furthermore, TTC staining demonstrated that infarct size was significantly reduced in all cardiac I/R groups treated with AAV-PTRF-KD compared with controls (Figure 8g,h). These results suggest that PTRF knockdown mitigates myocardial damage by suppressing TLR4/Myd88 signaling in cardiac I/R rats subjected to HFD and CIA.

## 4. Discussion

In this study, we established a novel rodent model combining CIA, HFD, and myocardial I/R injury. This model reproduces the synergistic effects of systemic inflammation, dyslipidemia, and acute ischemic stress, reflecting the multifactorial burden observed in patients with RA and CHD. Importantly, it enabled us to identify PTRF/cavin-1 as a critical mediator linking caveolae dysfunction to activation of innate immune signaling pathways in the ischemic myocardium.

CIA and HFD significantly aggravated myocardial damage in I/R rats, as evidenced by larger infarcts, more extensive fibrosis, greater cardiomyocyte apoptosis, and higher levels of cardiac injury biomarkers. These results support the concept that systemic inflammation and dyslipidemia act in concert to worsen ischemic outcomes, converging on endothelial dysfunction, vascular lipid accumulation, and extracellular matrix remodeling. Notably, severe collagen deposition and vascular pathology were observed in CIA+HFD groups even without I/R (Figure 4 and Figure 5), indicating that chronic inflammatory and metabolic stressors create a primed environment for ischemic injury.

As a scaffolding protein essential for caveolae formation, PTRF has been implicated in lipid homeostasis, vascular integrity, and stress signaling. Our transcriptomic profiling revealed enrichment of genes related to membrane raft organization, with PTRF emerging as one of the most consistently upregulated transcripts in the CIA + HFD + /R model and in independent human disease datasets. Immunofluorescence localized PTRF predominantly to vascular structures in the ischemic border zone, where increased CD31/PTRF double-positive cells suggest a role in endothelial–immune interactions. Our results here further position PTRF as a central node in coordinating inflammatory and metabolic cues during cardiac ischemia.

Evidence supports that the TLR4/MyD88/MAPKs signaling pathway serves as a key receptor mediating rapid responses in cardiac ischemic injury, which may be associated with the activation of oxidative stress, inflammation, and apoptosis through the induction of IL-1 and TNF-α production during myocardial I/R injury [30,31]. Additionally, studies have shown that PTRF impairs TLR4/MyD88 complex formation, whereas LPS enhances PTRF-TLR4 co-localization and interaction in lipid rafts. This suggests that LPS recognition induces TLR4 and its downstream components to translocate from non-raft to lipid raft regions of the plasma membrane, and PTRF is essential for TLR4 signaling assembly (especially post-LPS stimulation) by retaining pathway components in lipid rafts to trigger downstream ERK, p38, and JNK cascades [32]. Our previous studies have also demonstrated that AAV-mediated PTRF knockdown reduces the TLR4/PTRF interaction, indicating a strong co-localization pattern between PTRF and TLR4 [33]. Furthermore, TLR4/PTRF co-expression was decreased when PTRF was knocked down by AAV-PTRF shRNA in rat models of PI-IBS, alcoholic fatty liver (AFL), and NAFLD. Moreover, downregulation of TLR4/PTRF interaction affects all MAPK pathways, including ERK, p38, and JNK [16,33,34]. However, the existence and role of PTRF-TLR4 co-localization in myocardial ischemia have not yet been reported. In the present study, we extended this paradigm to the heart, showing that AAV-mediated PTRF knockdown attenuates TLR4/Myd88–JNK activation, reduces infarct size, and mitigates myocardial damage in I/R rats subjected to CIA and HFD (Figure 8). These findings suggest that modulation of PTRF or its downstream TLR4/Myd88–JNK axis could represent a therapeutic approach for cardiovascular complications in RA and metabolic syndrome. Future studies should address the cell-specific contributions of PTRF (endothelial, cardiomyocyte, and immune compartments), as well as its role in chronic remodeling and plaque vulnerability. Translational studies in RA and CHD patients are warranted to evaluate PTRF as a potential biomarker or target for therapeutic intervention.

In summary, this study has established a novel animal model of atherosclerosis combined with rheumatoid arthritis-associated coronary heart disease (RA-CHD), which faithfully recapitulates the clinical pathophysiological processes of comorbid conditions. Specifically, the HFD-induced model mimics the progressive development of both atherosclerosis and rheumatoid arthritis, characterized by hyperlipidemia, atherosclerotic plaque deposition, endothelial injury, inflammatory activation, collagen accumulation, fibrous plaque formation, vascular luminal stenosis, and subsequent myocardial ischemia. Due to limitations in manuscript length, this study mainly focuses on the role of HFD and RA in exacerbating atherosclerosis and myocardial ischemia, as well as the underlying mechanisms, specifically the promotion of PTRF-TLR4 colocalization in ischemic myocardium. However, the direct impact of the combination of CIA and HFD on myocardial ischemia and the related dynamic changes and regulatory mechanisms at different disease stages remain unexplored. Additionally, ethical constraints restrict the clinical translation of the findings derived from this animal model. Our results from Figure 4, Figure 5 and Figure 7 demonstrate that the combination of cardiac ischemia-reperfusion (I/R), CIA, and HFD significantly upregulates PTRF expression in damaged myocardium and exacerbates myocardial injury to a greater extent than CIA and/or HFD alone, while CIA and/or HFD alone can also induce mild cardiac injury (with the relevant mechanisms to be further clarified). Furthermore, in this study, the ST segment changes in ECG were mainly used as a model for successful establishment and supportive electrophysiological evidence, rather than as a core indicator for precisely quantifying the degree of injury. The severity of myocardial injury is mainly evaluated through more objective quantitative methods such as infarction area measurement, serum myocardial enzymes, and pathology data. Future studies will address these research gaps by integrating clinical data to further elucidate the pathogenesis of this comorbidity and its therapeutic implications.

## 5. Conclusions

In conclusion, we developed a novel model using a combination of CIA, hyperlipidemia and cardiac I/R injury, which reflects the TCM view of “disease”—“syndrome” interactions, emphasizing a holistic approach to understanding these complex co-morbidities. Using this model, we identified PTRF as a central regulator of TLR4/Myd88–JNK signaling, linking caveolae dysfunction to inflammatory amplification and myocardial injury. These findings provide mechanistic insight into how systemic inflammation and metabolic dysregulation converge on cardiac ischemic pathology, which may open avenues for therapeutic targeting in high-risk RA patients.

## Figures and Tables

**Table 1:** Primer sequences for qPCR of specific genes.

Specific Genes	Primer Sequences
*Adcy3*	F: 5′-AGCACCTATATGGCAGCTTCTGGA-3′ R: 5′-TAAGCGTGTCCTTCATGGCTAGTG-3′
*Ptprc*	F: 5′-GTGTTCAGCCAGCTGATCC-3′ R: 5′-CAGATTCCACGGACCACTG-3′
*Ano1*	F: 5′-GGGAGAAGCAACACTTATTCGA-3′ R: 5′-TGCACGTTGTTCTCTTCAGGAT-3′
*PTRF*	F: 5′-GTCAGCGTCAACGTGAAGA-3′ R: 5′-AGCTTGACTTCATCCTGGTAG-3′
*Prkar2b*	F: 5′-GGAGAACGAGCGCAAGG-3′ R: 5′-CCGGTTTATAACTGGAGCGTT-3′
*Cd2*	F: 5′-TTGTCAACTGTCCAGAGAAAGG-3′ R: 5′-CAGAAAATAAACAGCGCCC-3′
*Cd28*	F: 5′-CTCAGTTCAAGTAACAGAAAACAA-3′ R: 5′-CATTCCCGACACAGACTTCC-3′
*Zap70*	F: 5′-GTGAGCTGTGGTAGCGTCG-3′ R: 5′-TCTAGTGCATCACCCTCCAGT-3′
*β-actin*	F: 5′-CTCTCTTCCAGCCTTCCTTC-3′ R: 5′-CTCTCTTCCAGCCTTCCTTC-3′

**Table 2:** Semi-quantitative histopathological scoring of myocardial tissue across experimental groups.

	Masson’s Trichrome Score (Fibrosis/Collagen Deposition)	H&E Score (Histological Injury)	TUNEL Score (Apoptosis)
Control	0	0	0
Cardiac I/R	0	2	3
HFD	2	1	1
CIA	2	1	1
Cardiac I/R + CIA	1	3	3
CIA + HFD	3	1	1
Cardiac I/R + HFD	1	2	2
Cardiac I/R + HFD + CIA	3	3	3

## Data Availability

The original contributions presented in this study are included in the article/Supplementary Materials. Further inquiries can be directed to the corresponding authors.

## Supplementary Materials

The following supporting information can be downloaded at: https://www.mdpi.com/article/10.3390/biom16050753/s1, Original images of Figure 7i and Figure 8c can be found in supplementary materials.
