# Supplementary material for: Compound Augmentation of Myocardial Injury in a Rat Model of Coronary Heart Disease Induced by Ischemia/Reperfusion, Rheumatoid Arthritis, and High-Fat Diet: A Molecular Mechanistic Study
Source: Biomolecules. 2026 May 21;16(5):753. doi: 10.3390/biom16050753 (PMC13204909; doi:10.3390/biom16050753)
Supplement: Supplementary file 1 [file biomolecules-16-00753-s001.zip › biomolecules-4275579-supplementary.pdf]

Figure 2C CIA_20.00X

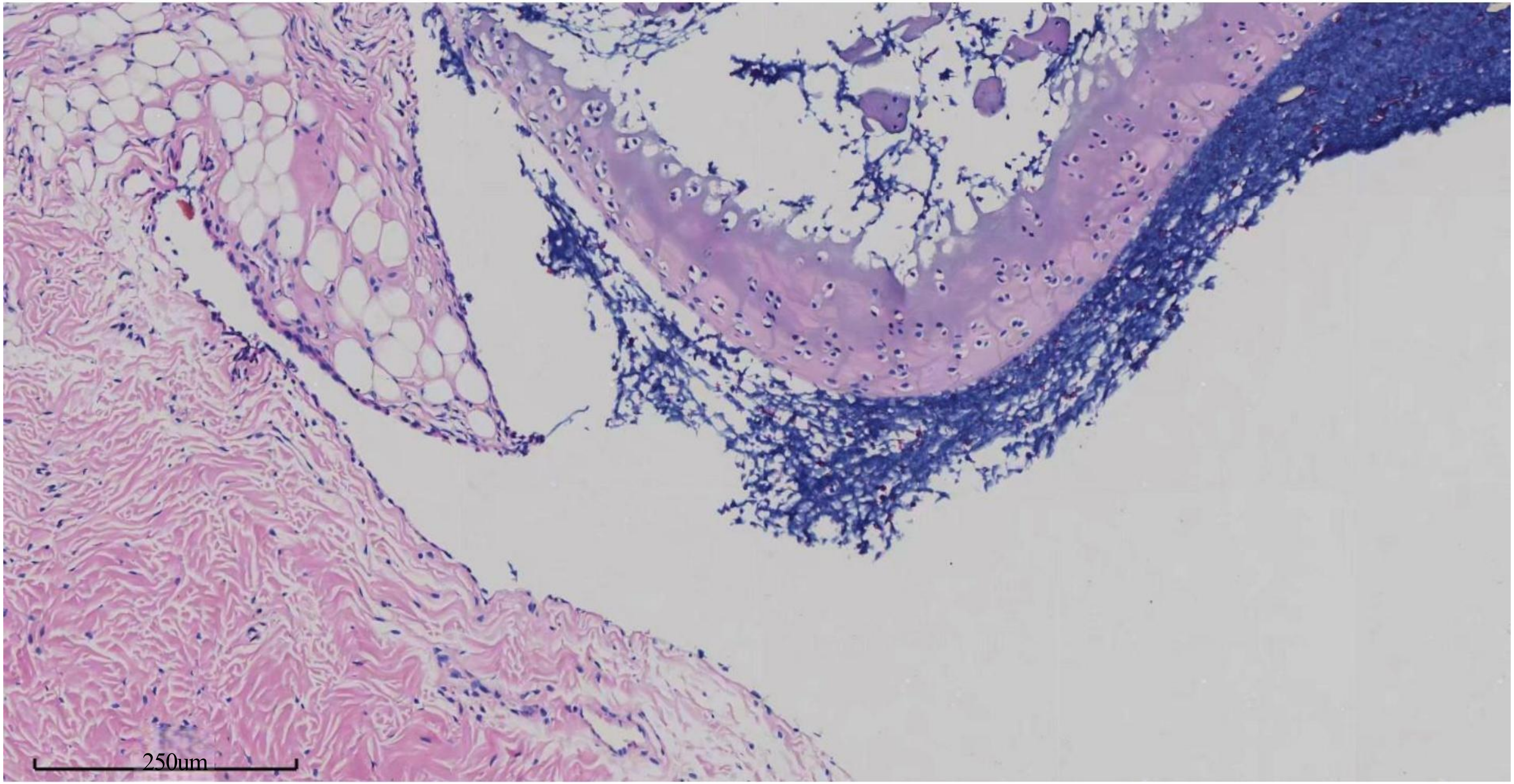

CIA+HFD_20.00X

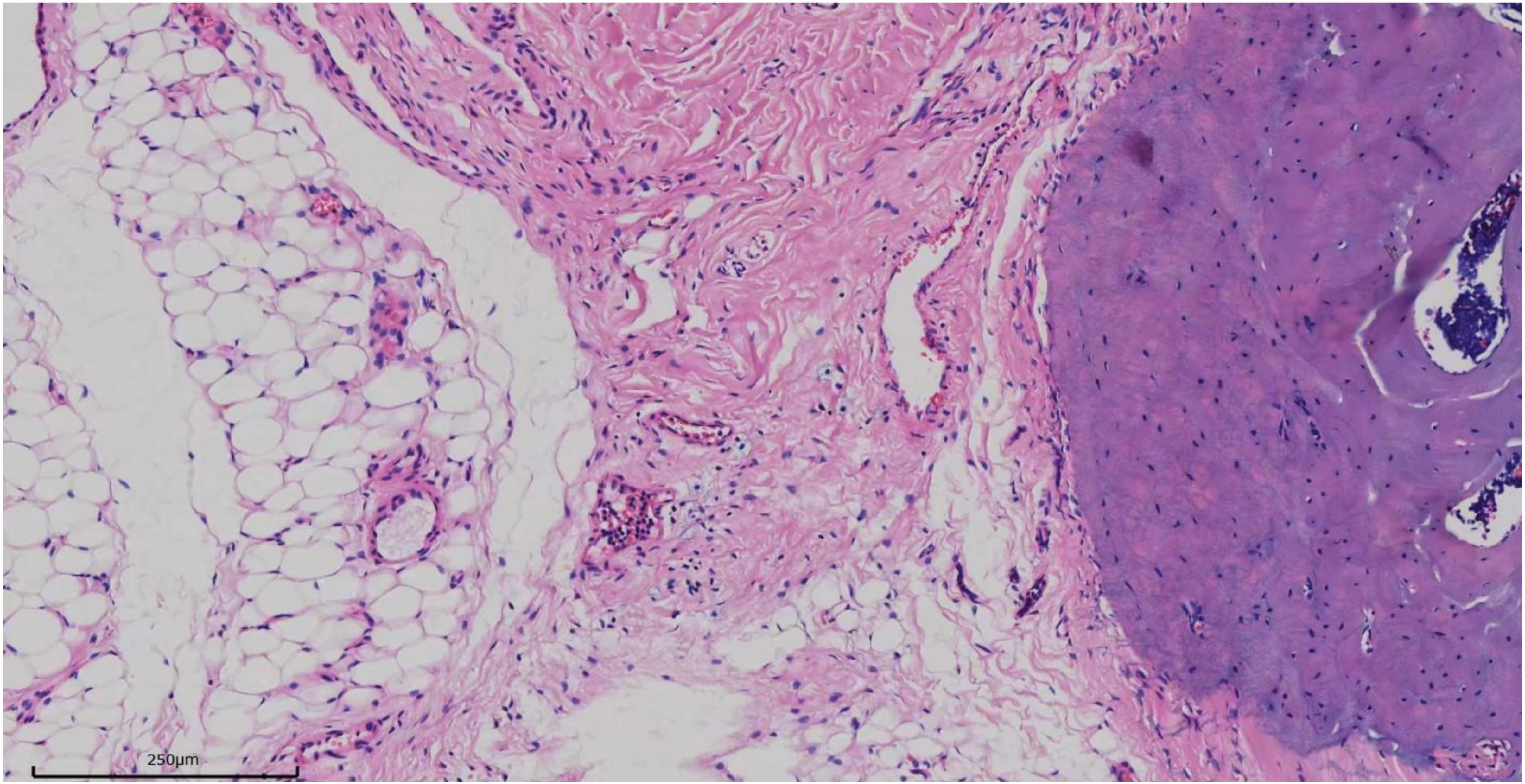

CIA+HFD+IR_20.00X

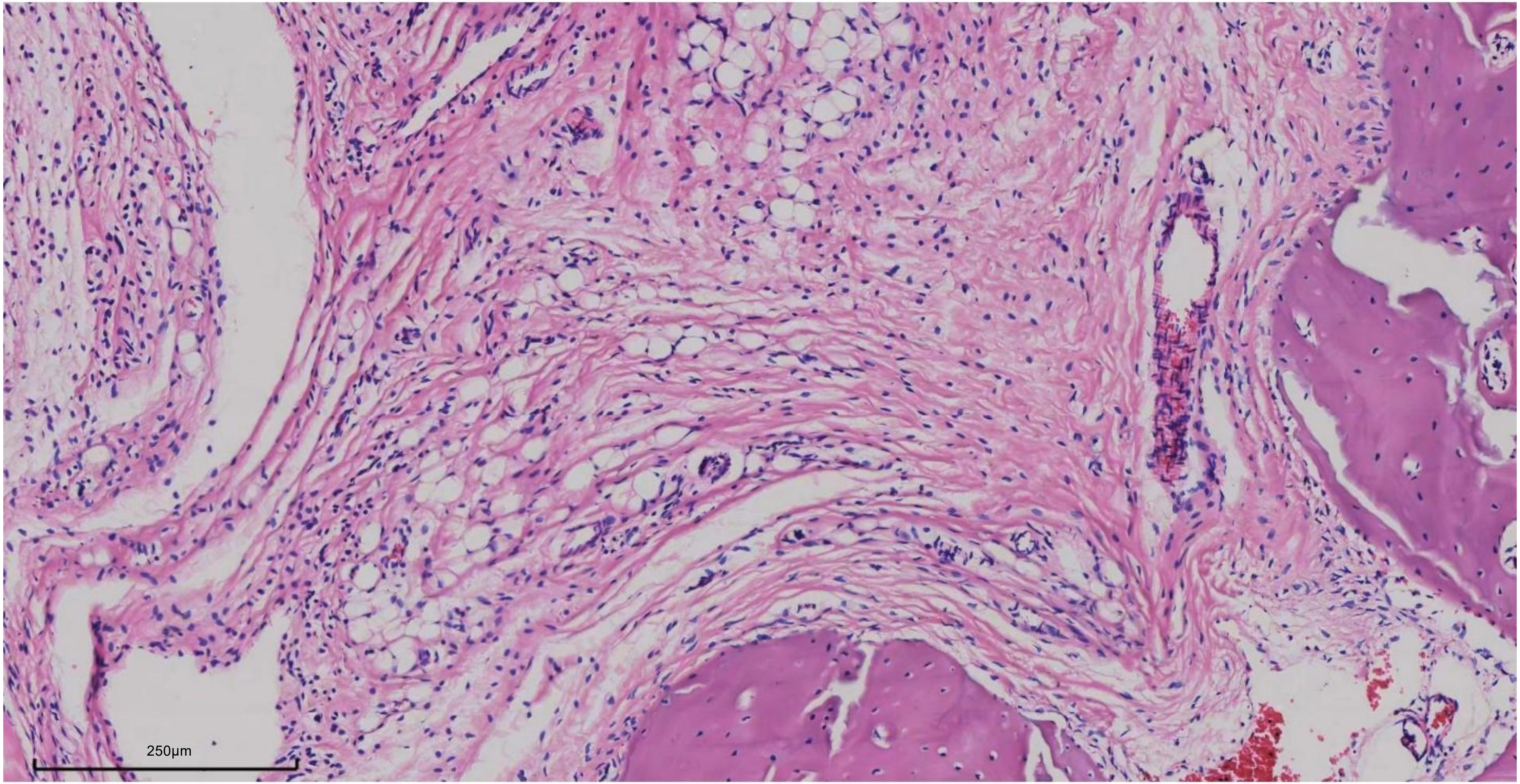

HFD_20.00X

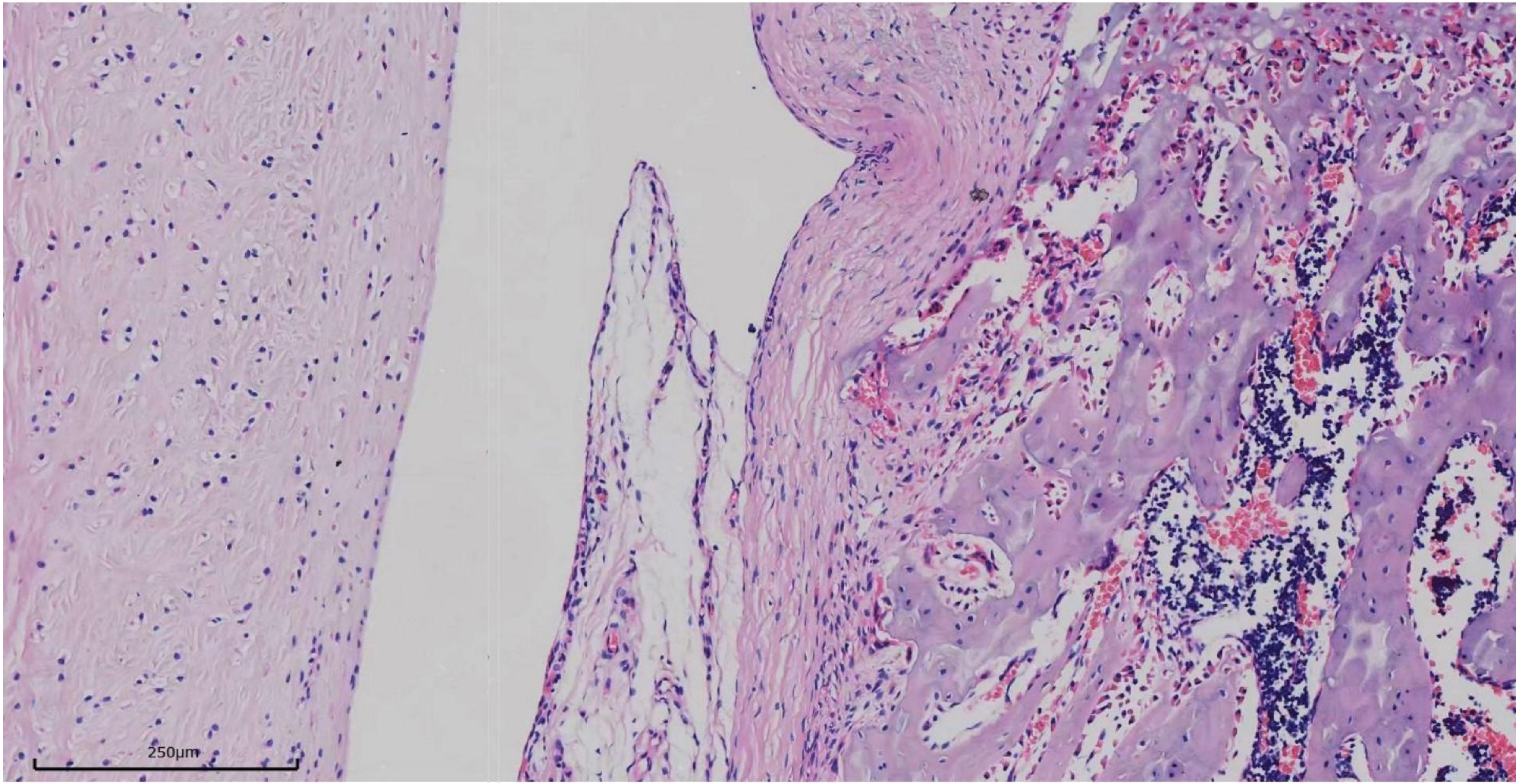

IR+CIA_20.00X

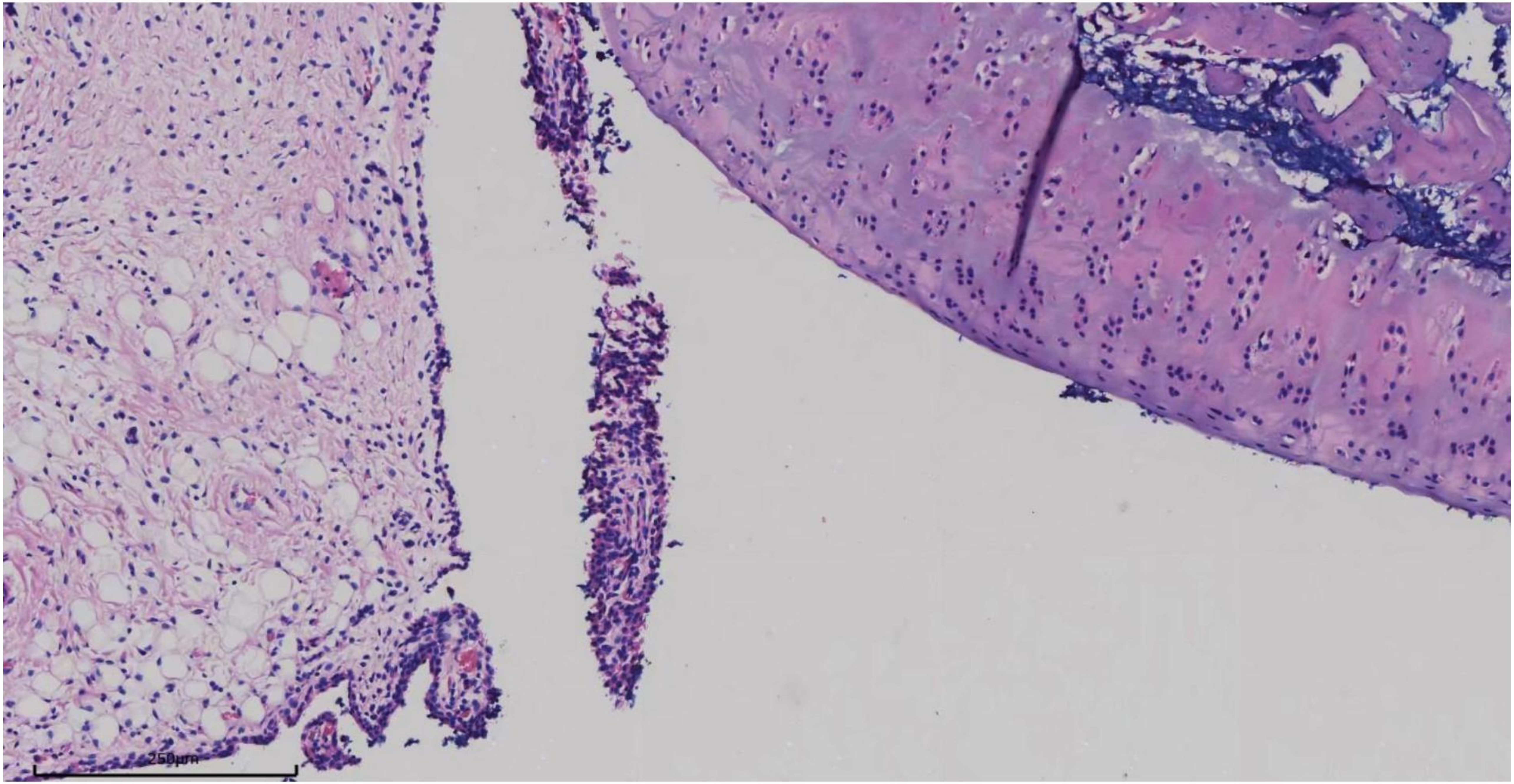

IR+CIA_20.00X

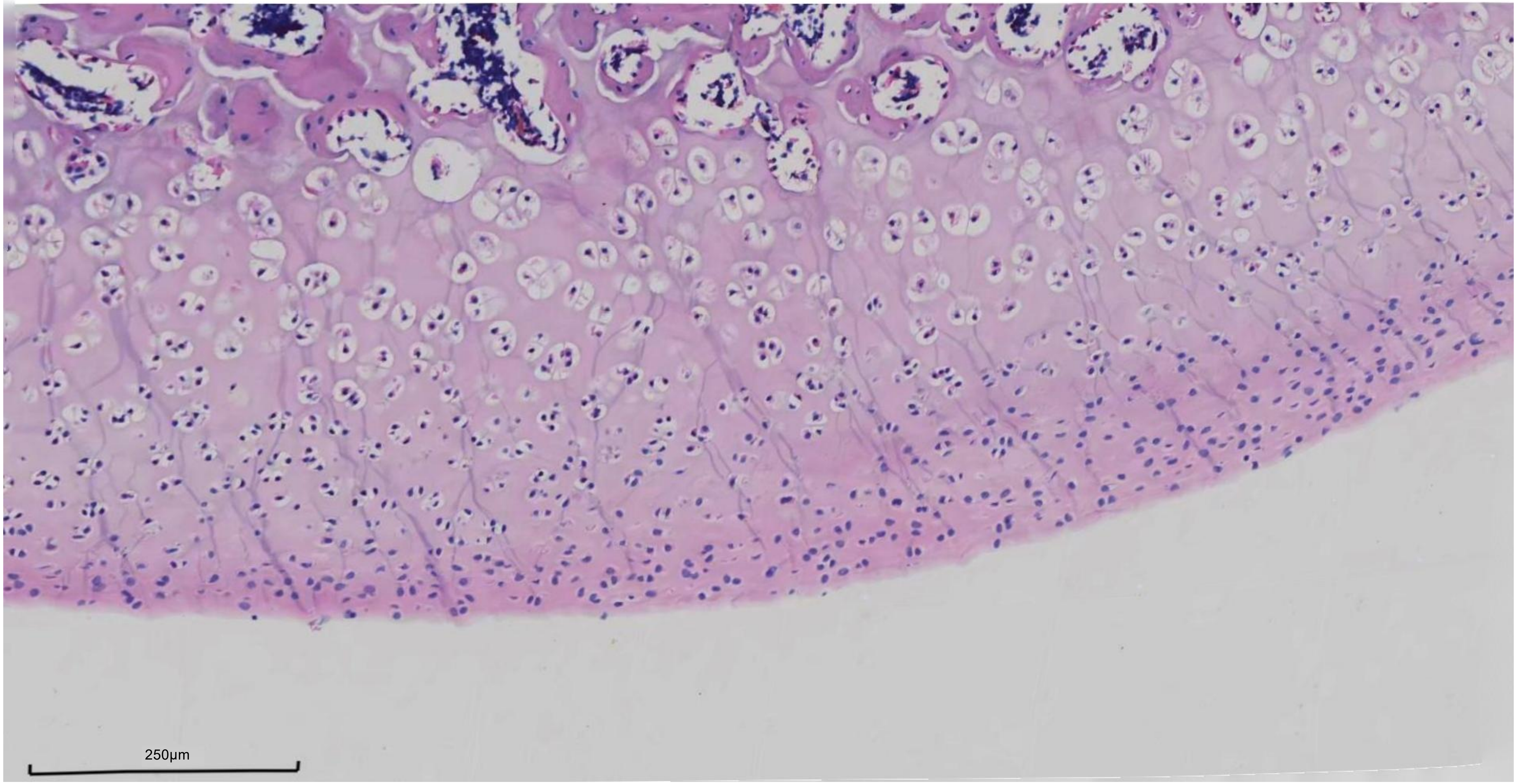

IR+CIA_20.00X

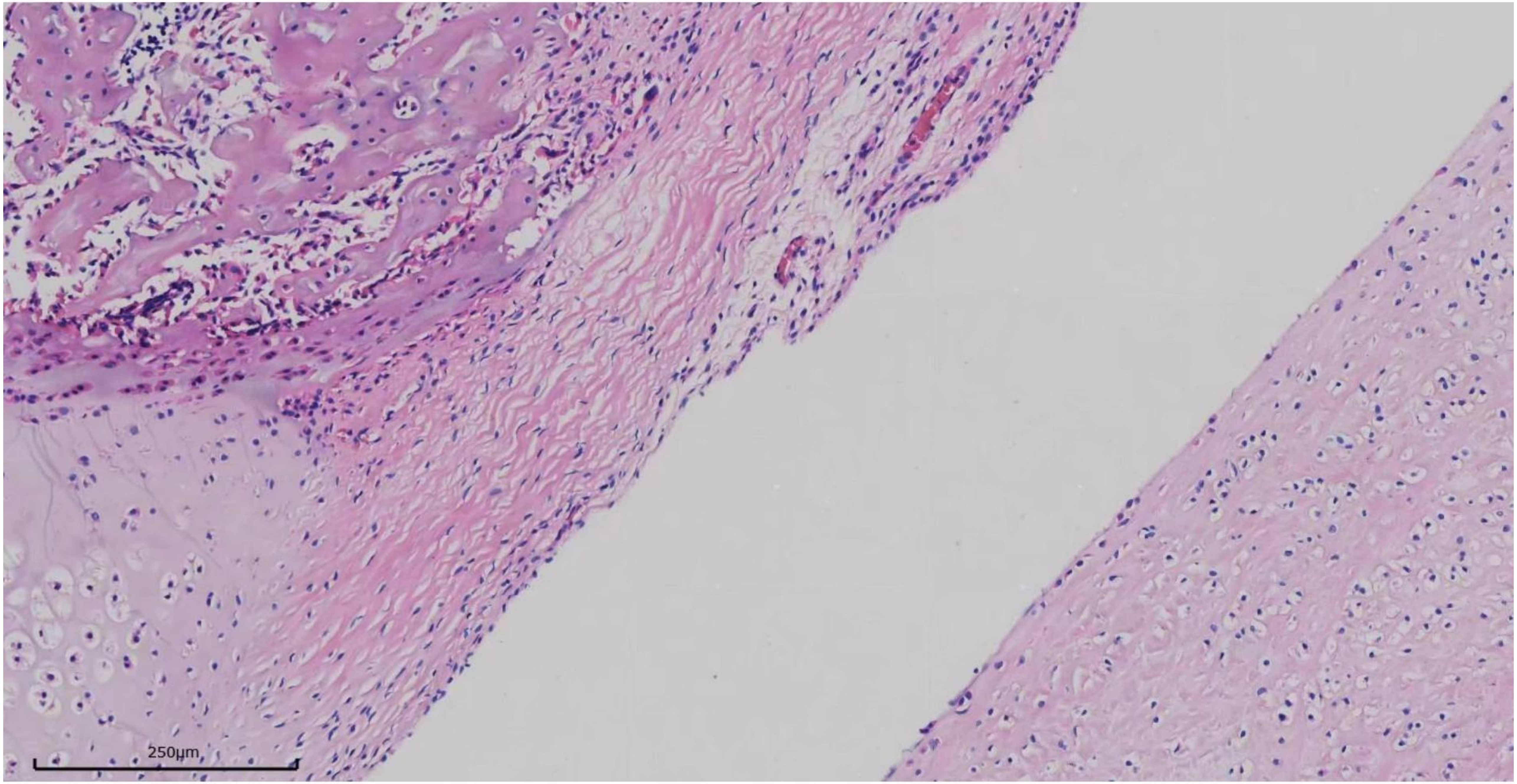

Normal_20.00X

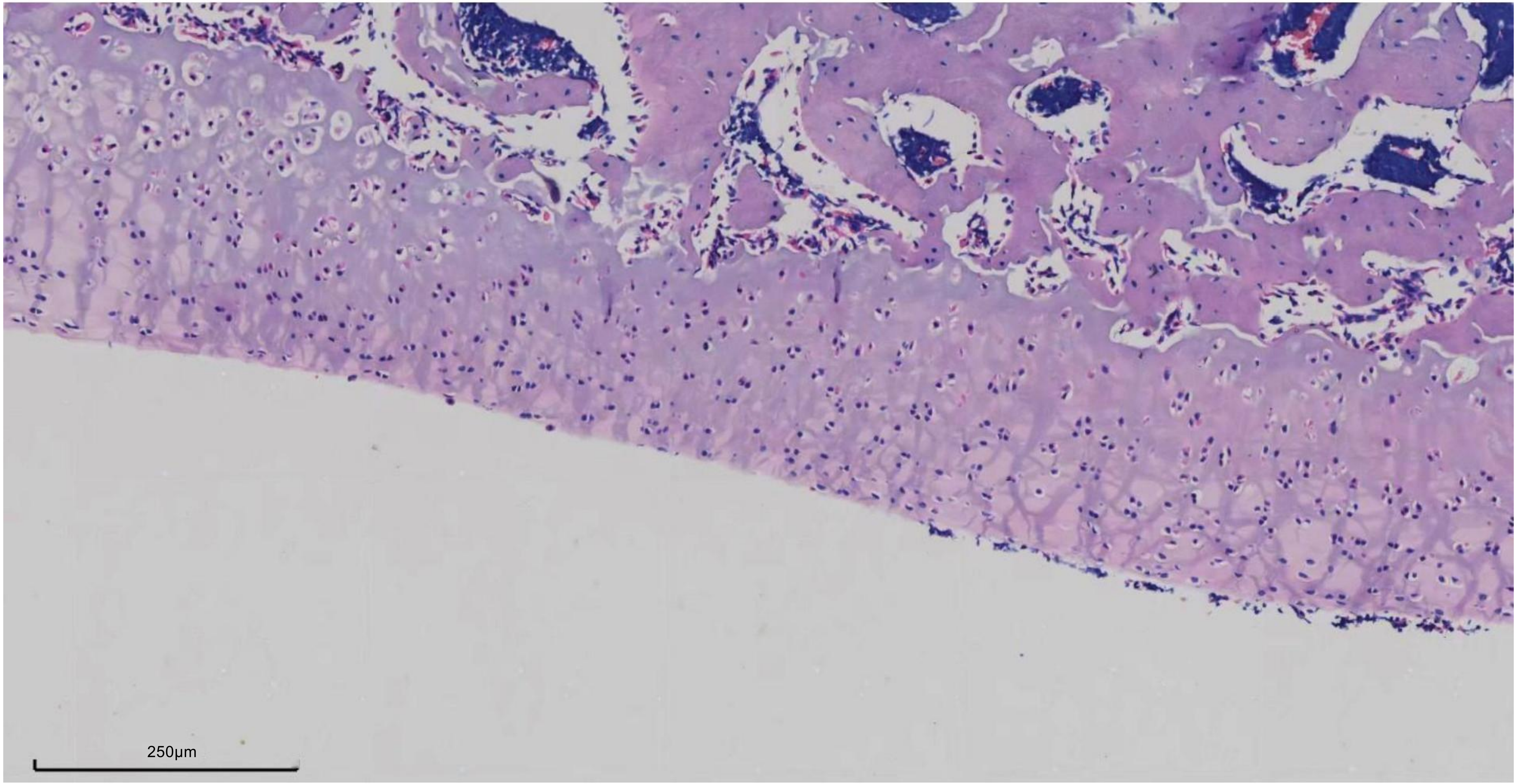

Figure 3J _20.00X

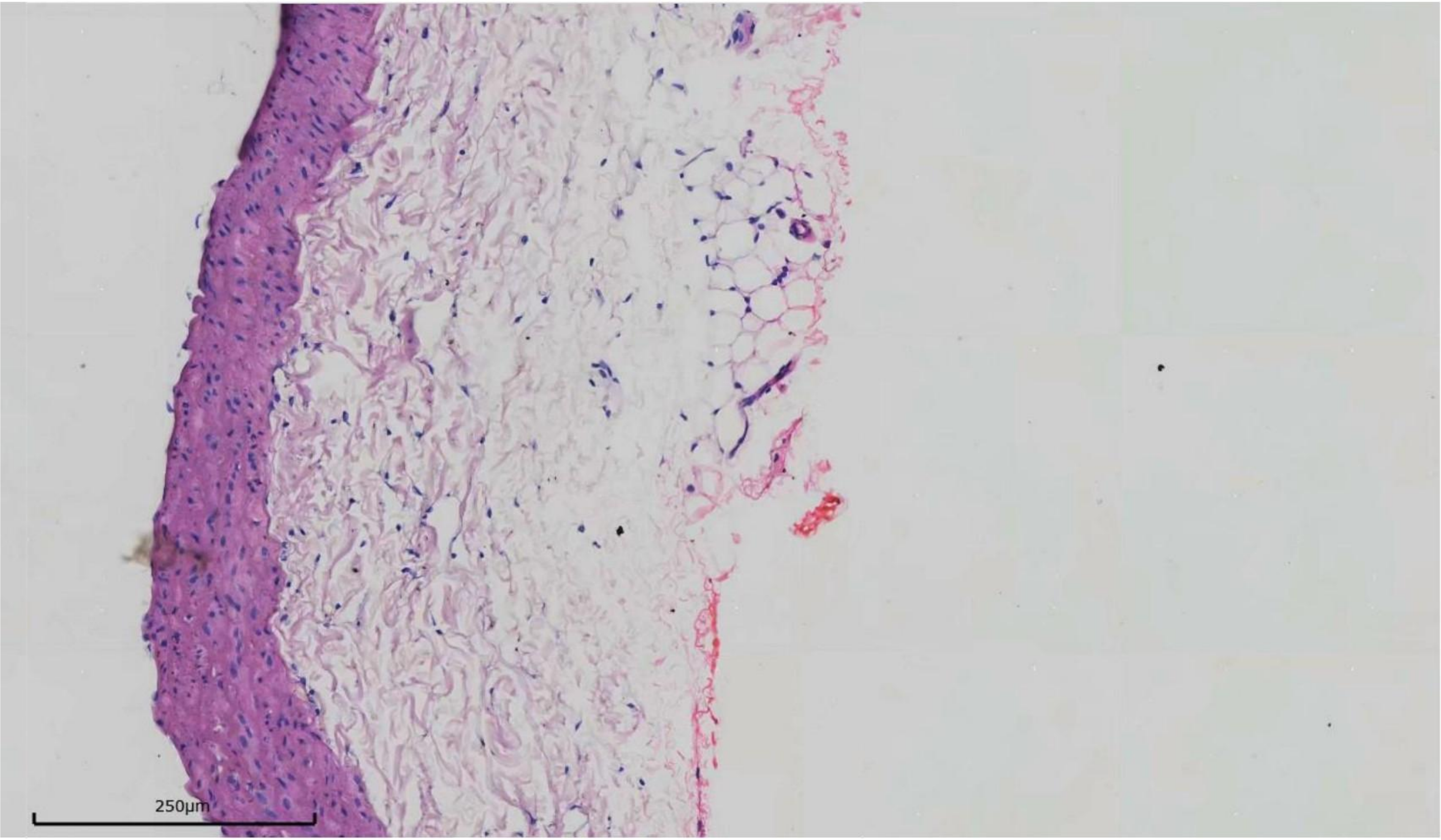

GJ

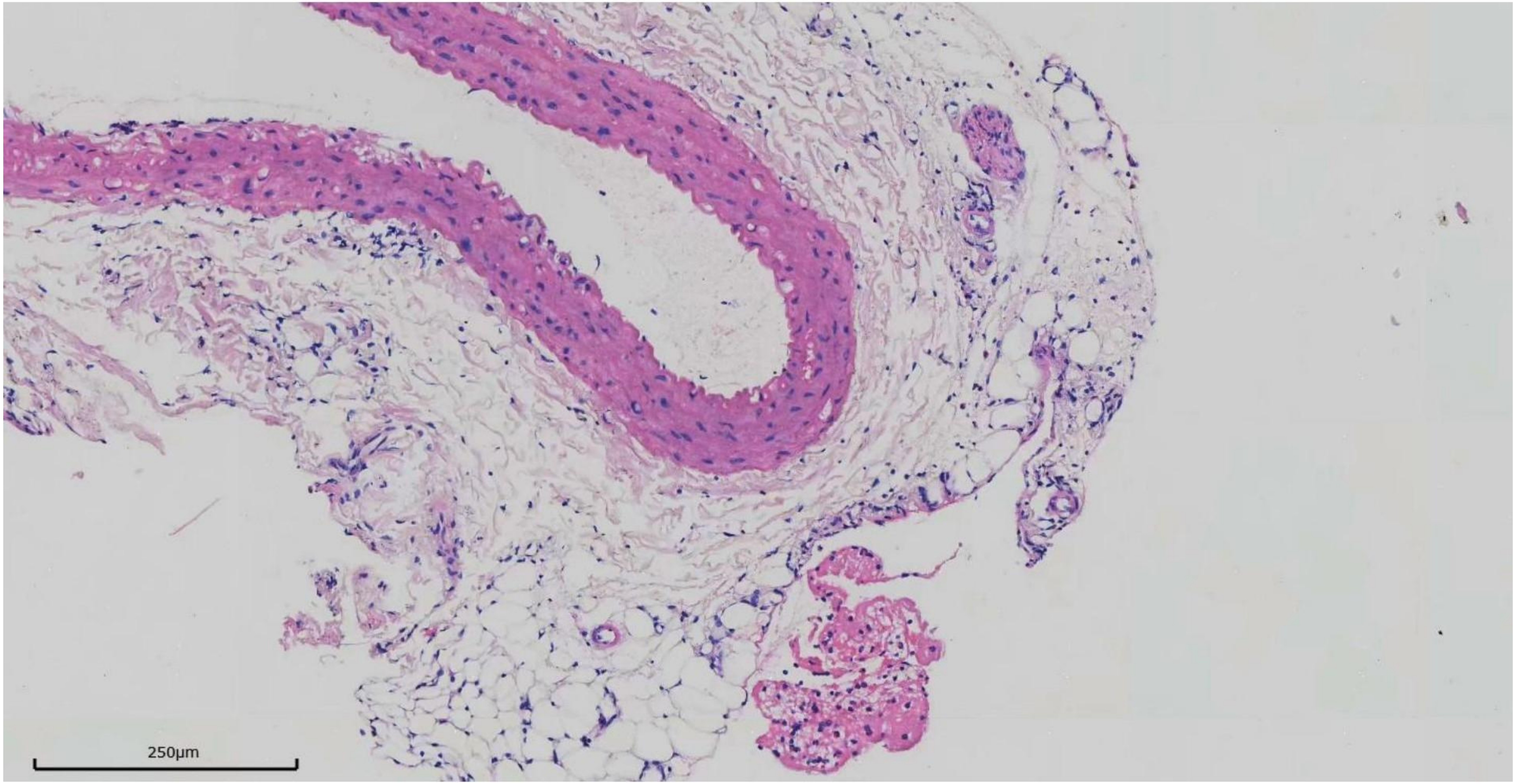

GJZ

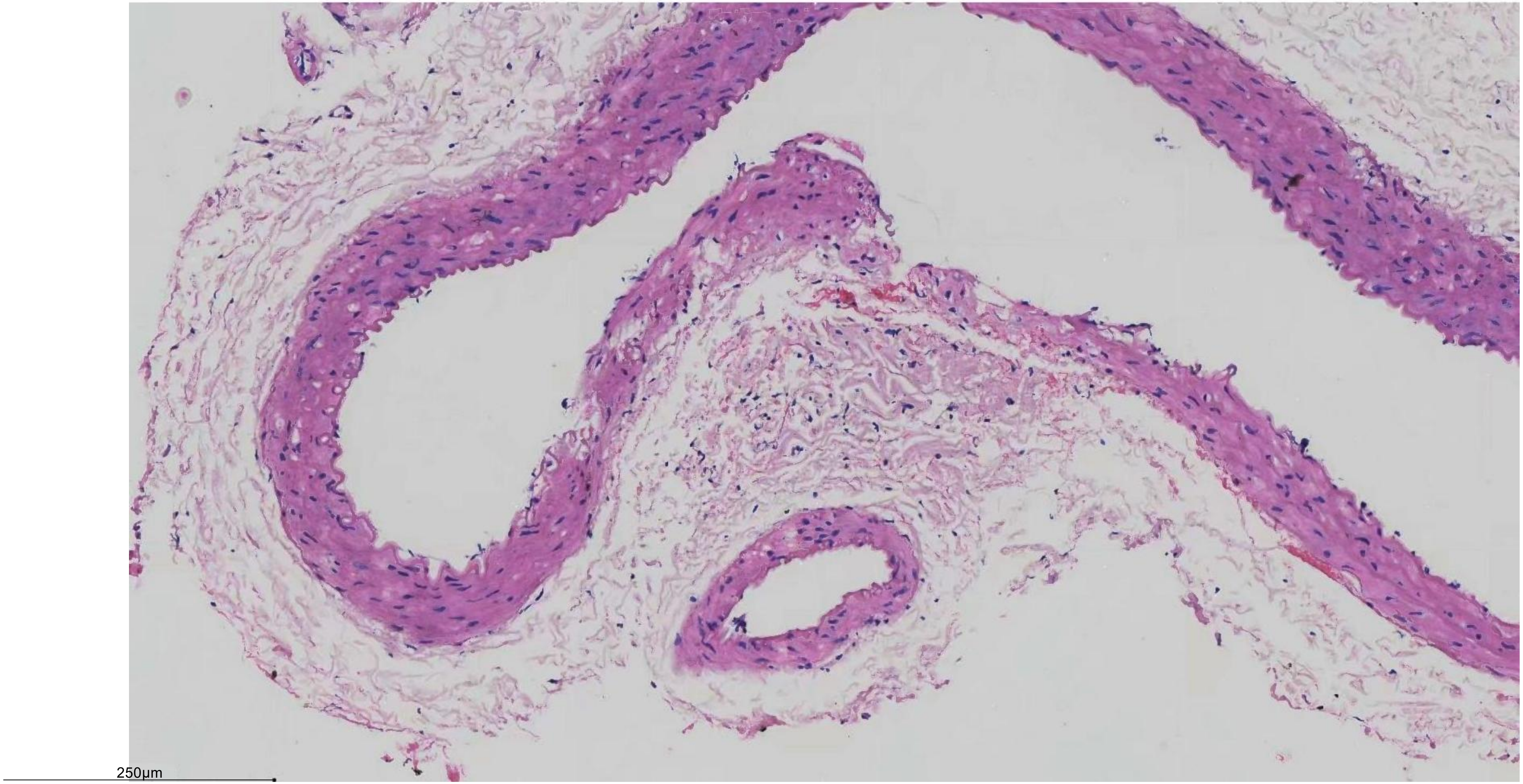

GZ 20.00X

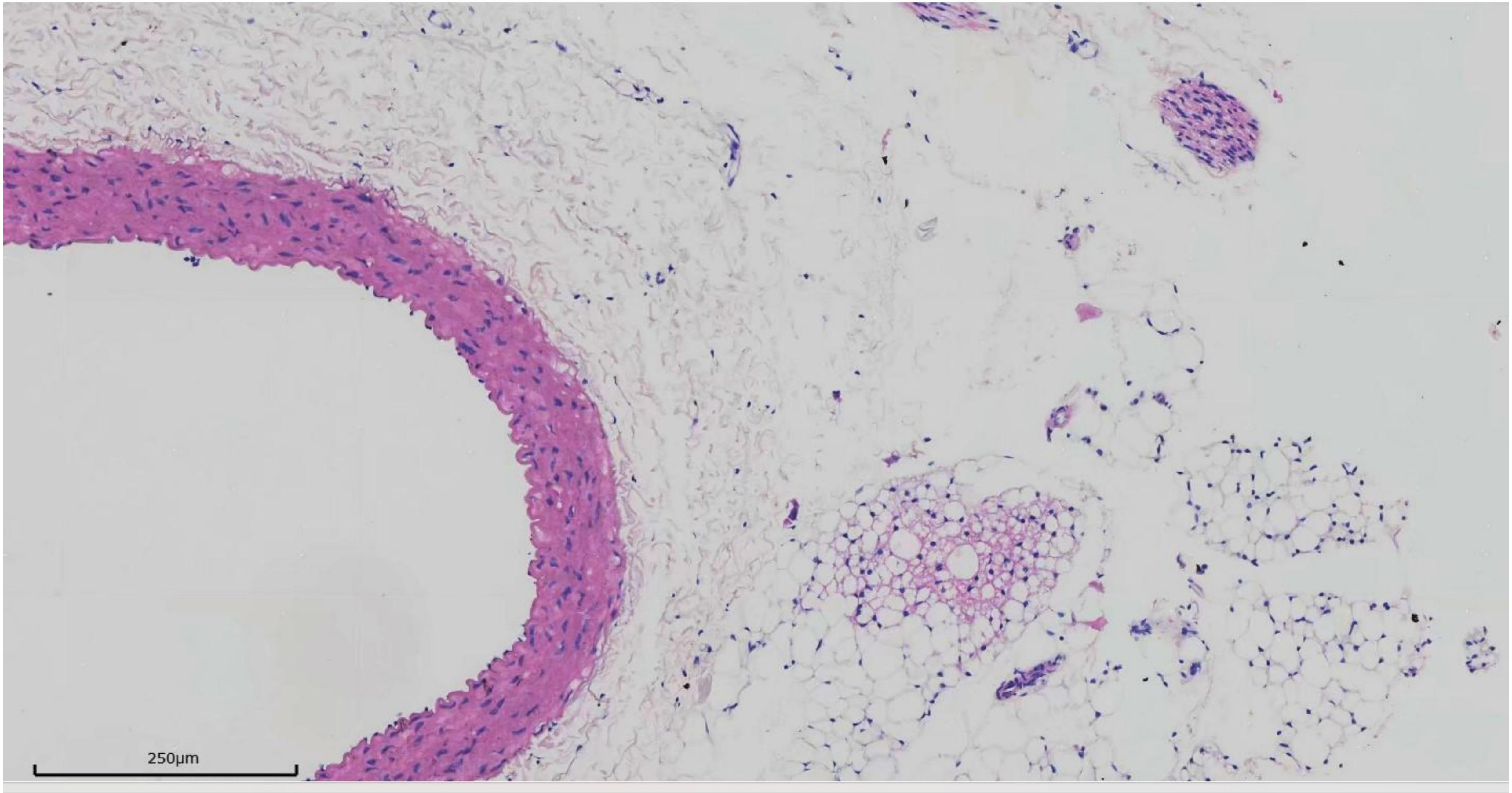

总

J_20.00X

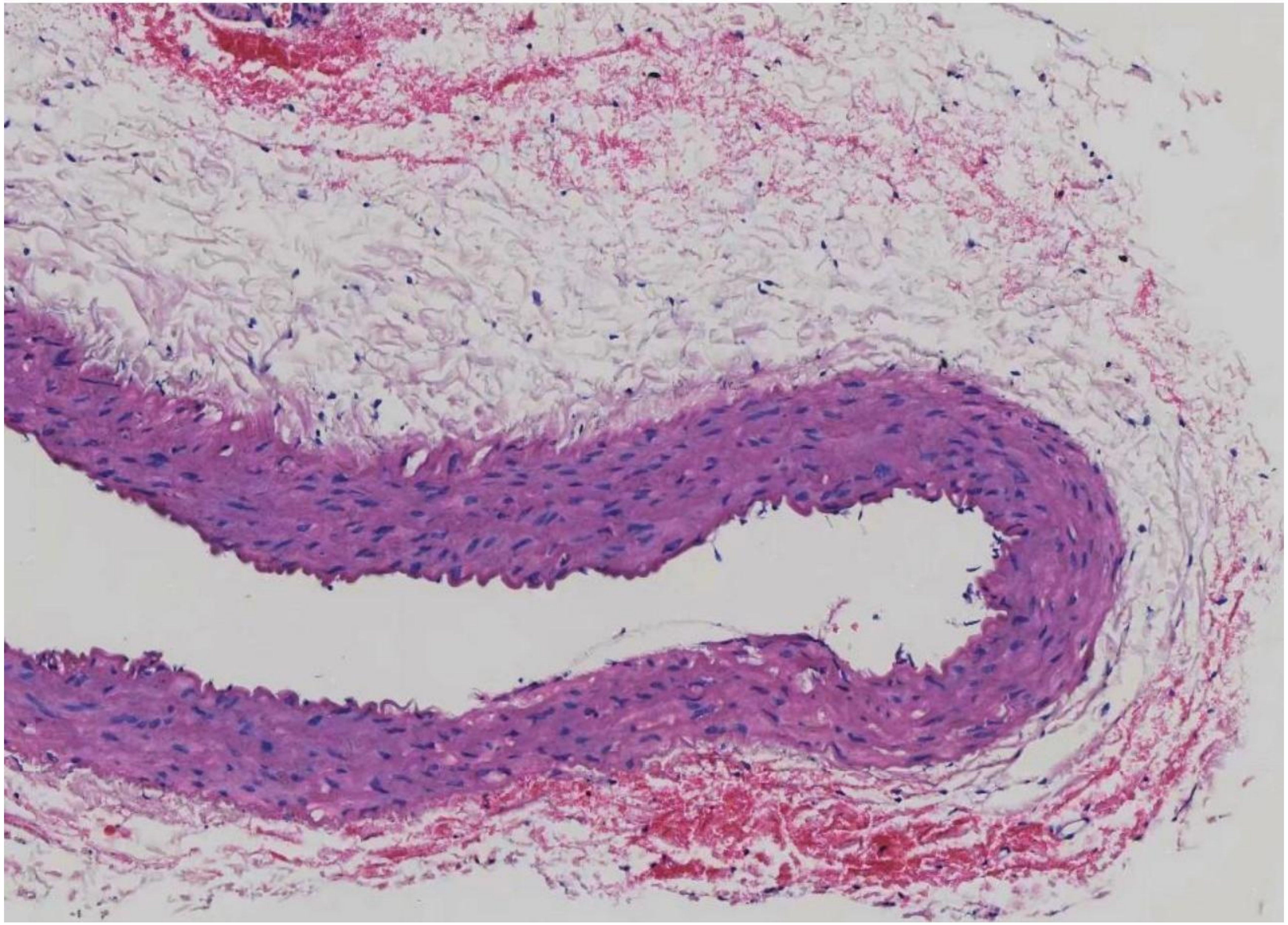

250μm

JZ_20.00X

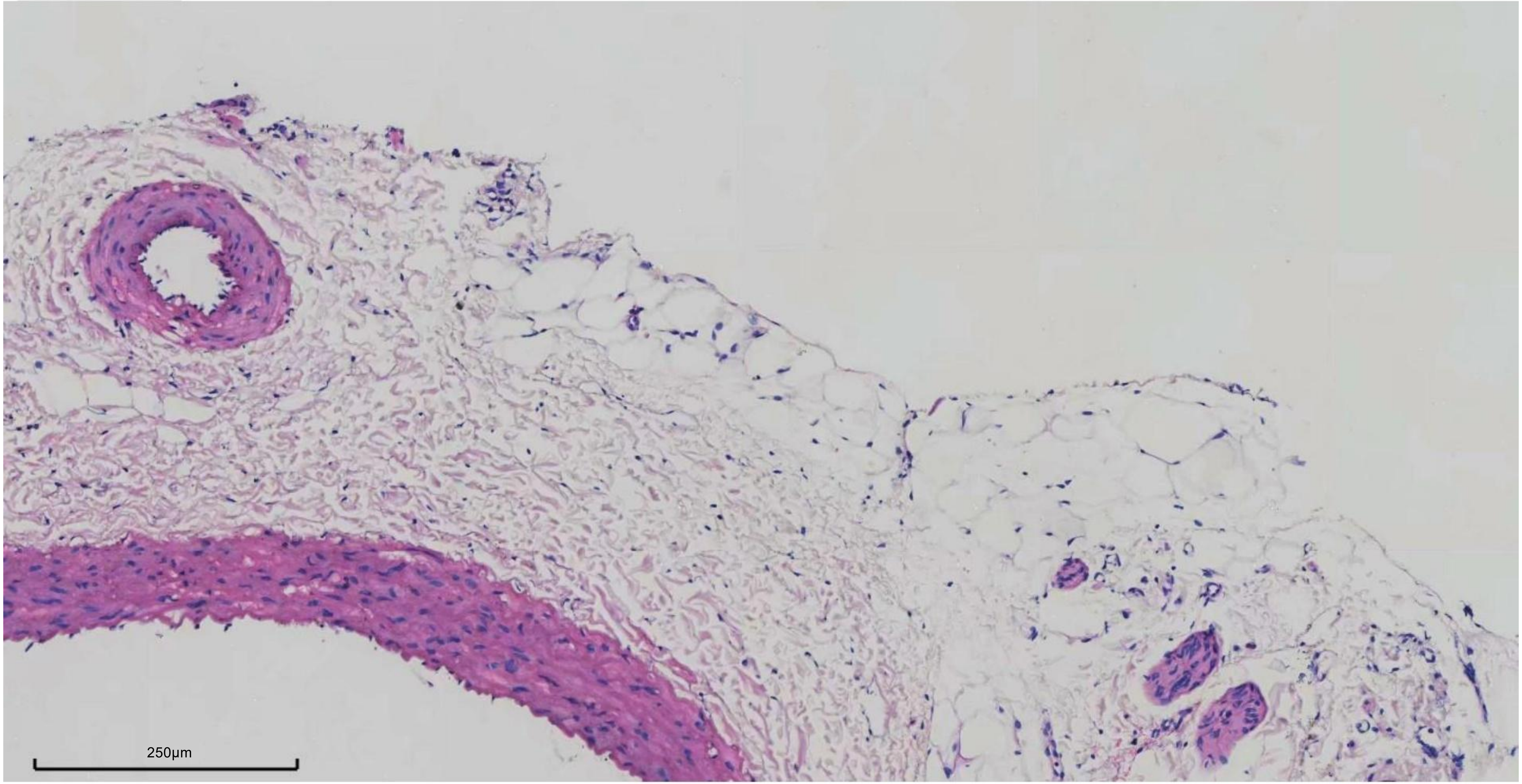

N_20.00X

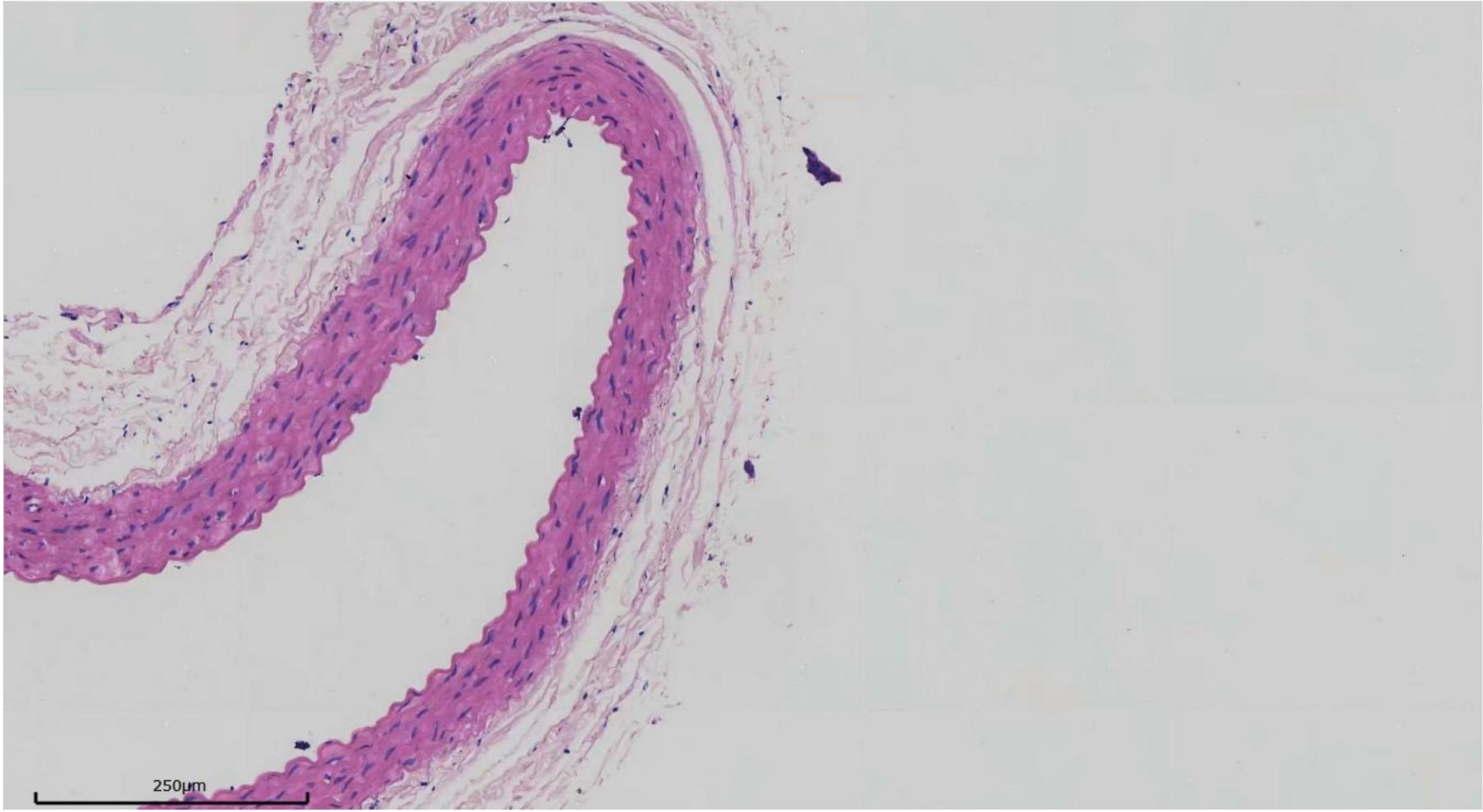

NZ_20.00X

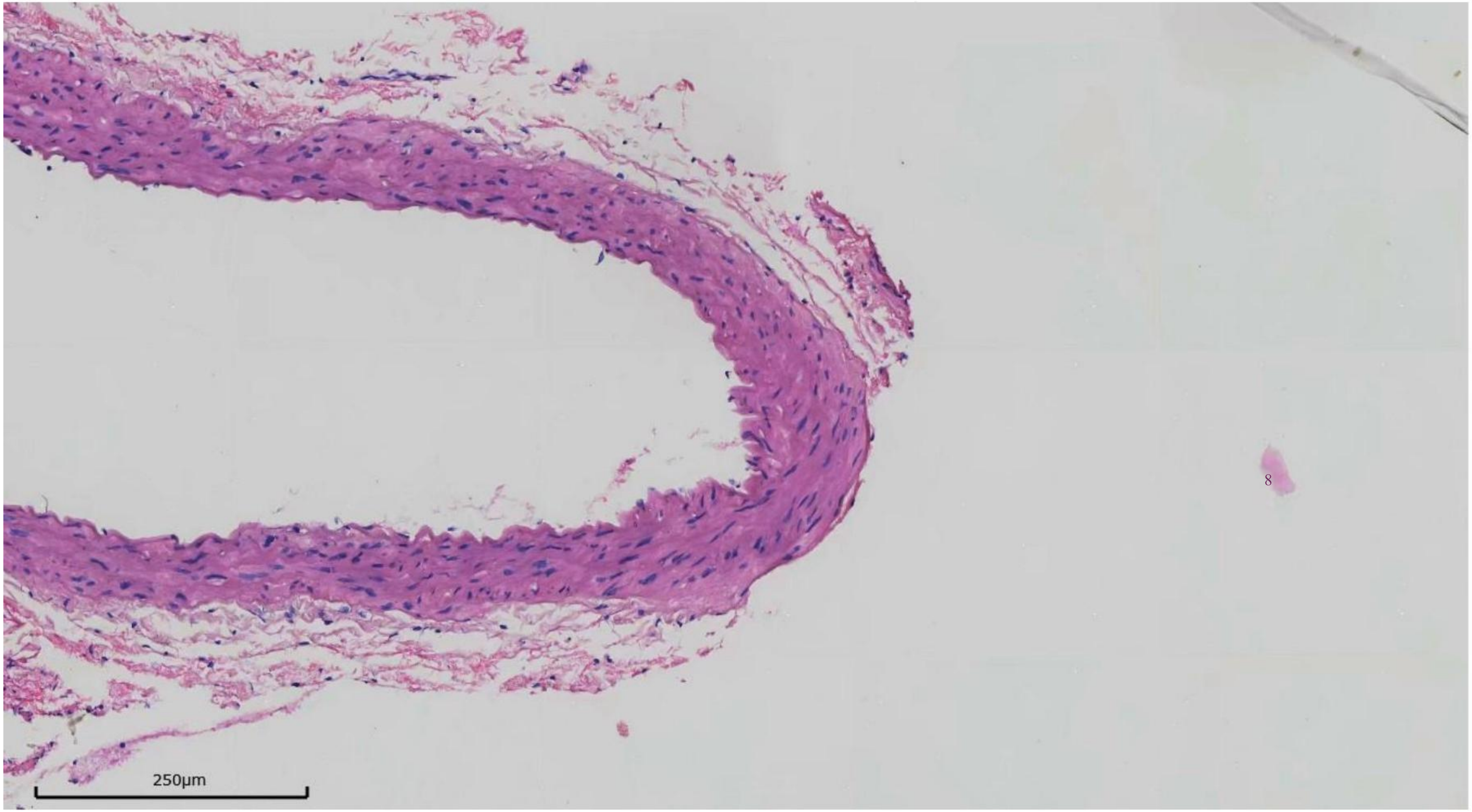

Figure 3 i

CIA 3_20.00X

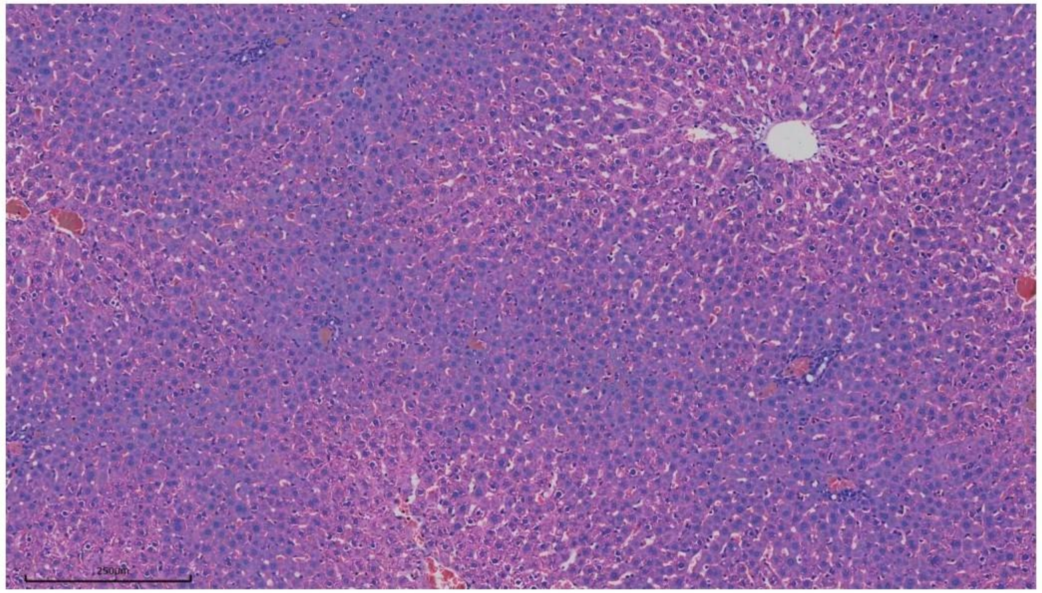

Figure 3 i

HFD +IR+CIA 2_20.00X

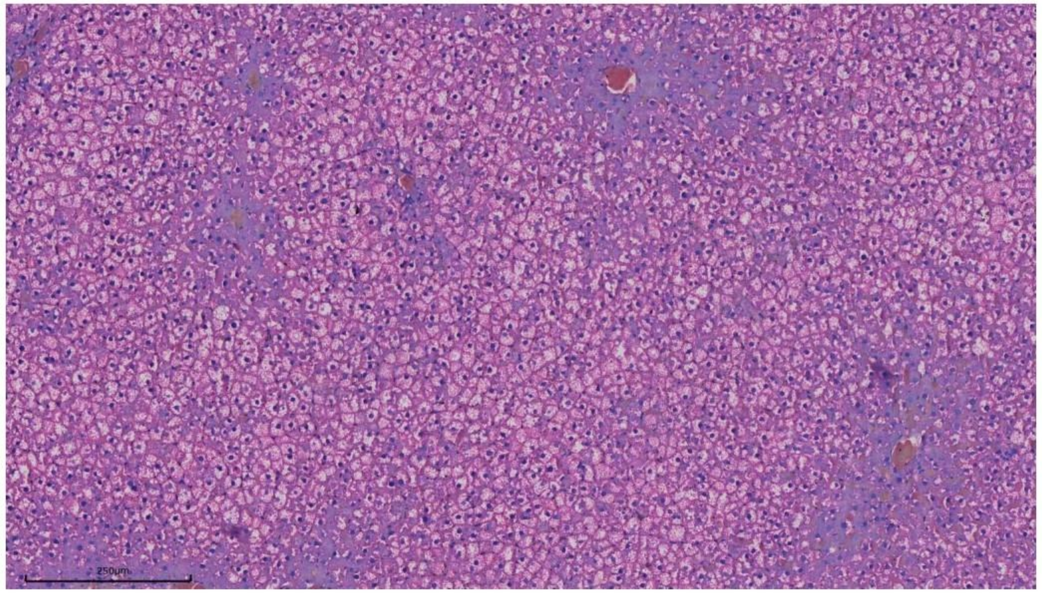

Figure 3 i

HFD 3_20.00X

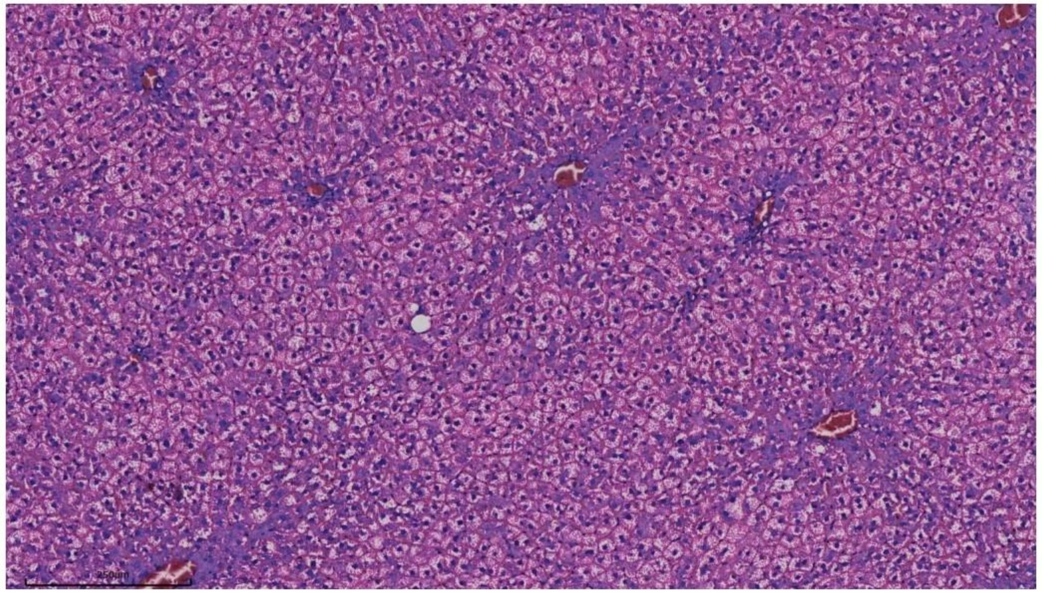

Figure 3 i

IR+CIA 3_20.00X

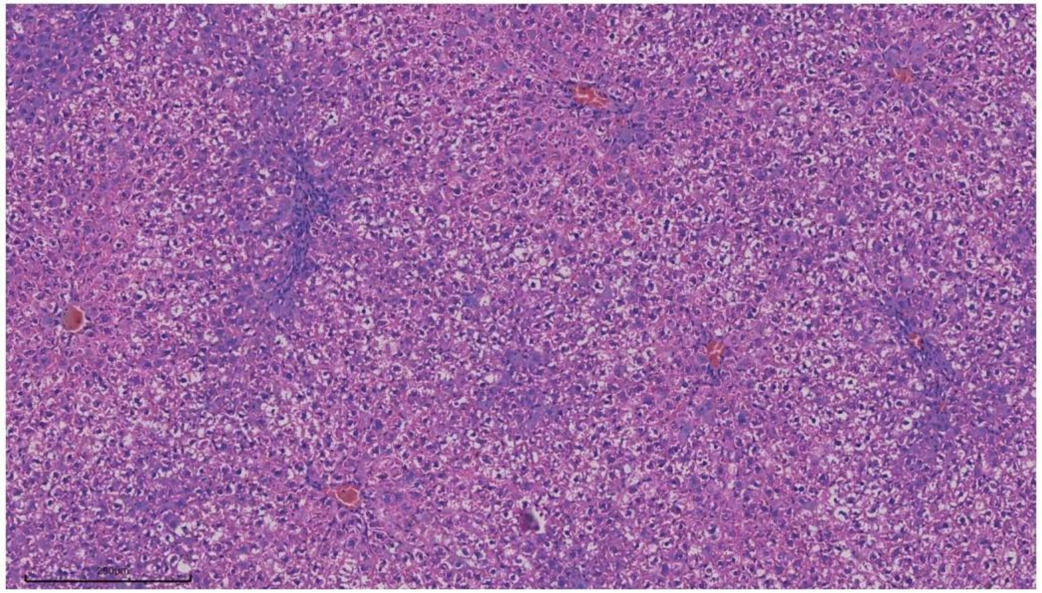

Figure 3 i cardiac_20.00X

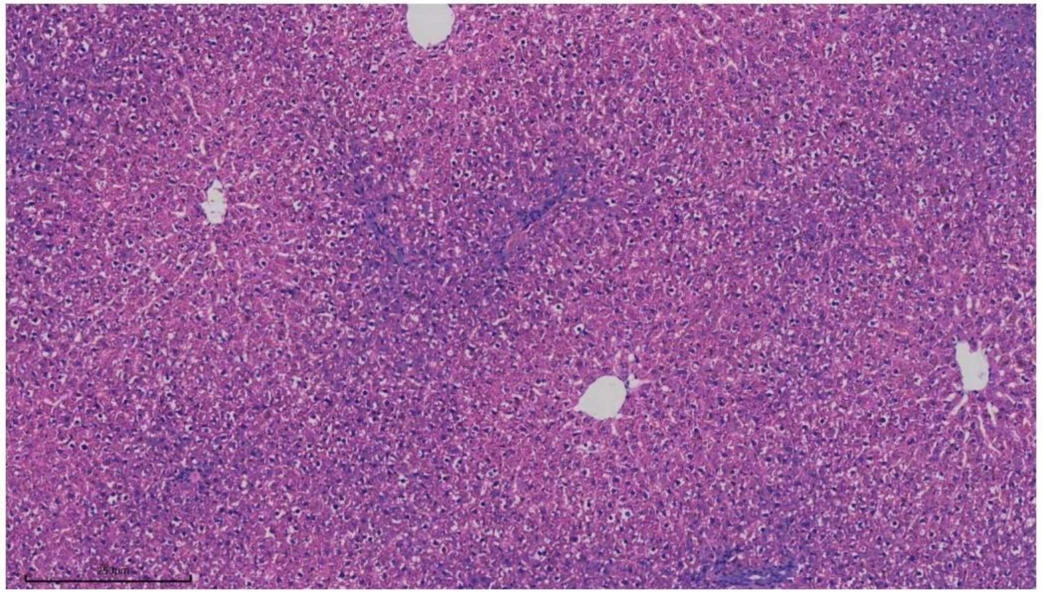

Figure 3 i

CIA+HFD 4_20.00X

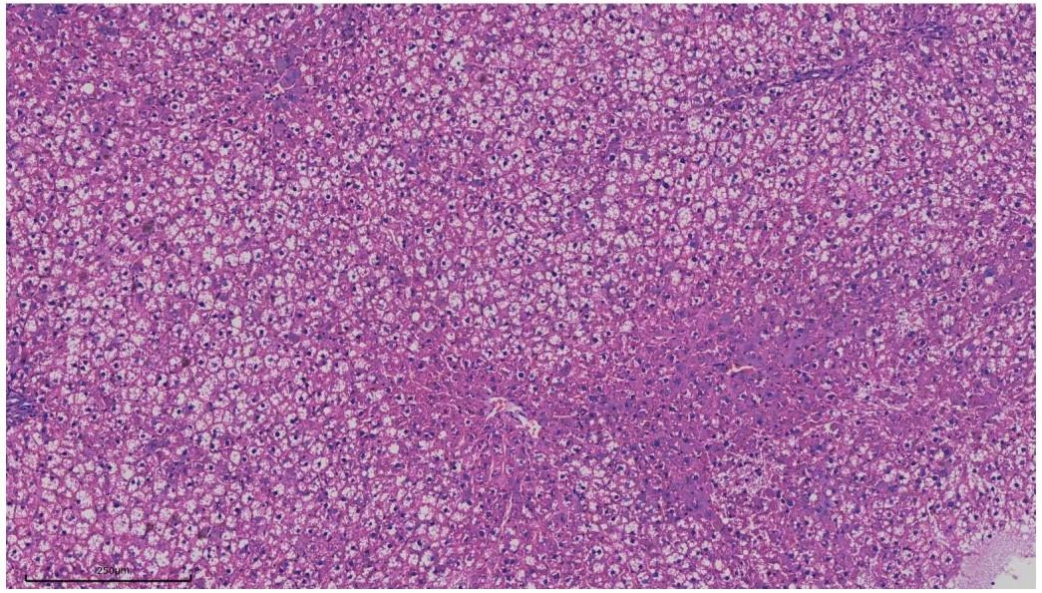

Figure 3 i

HFD+IR 3_20.00X

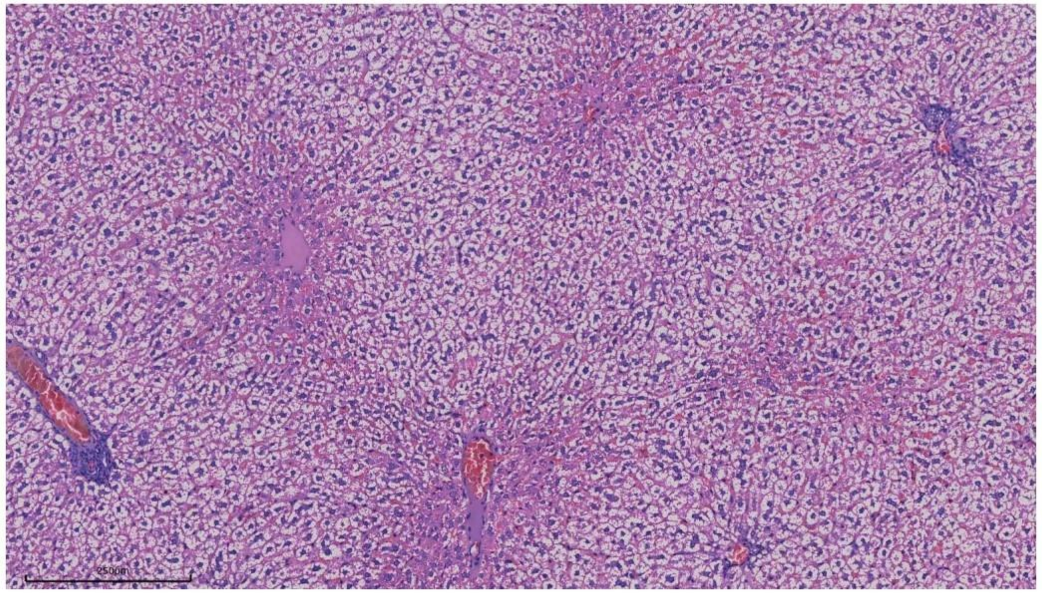

Figure 3 i

Normal_20.00X

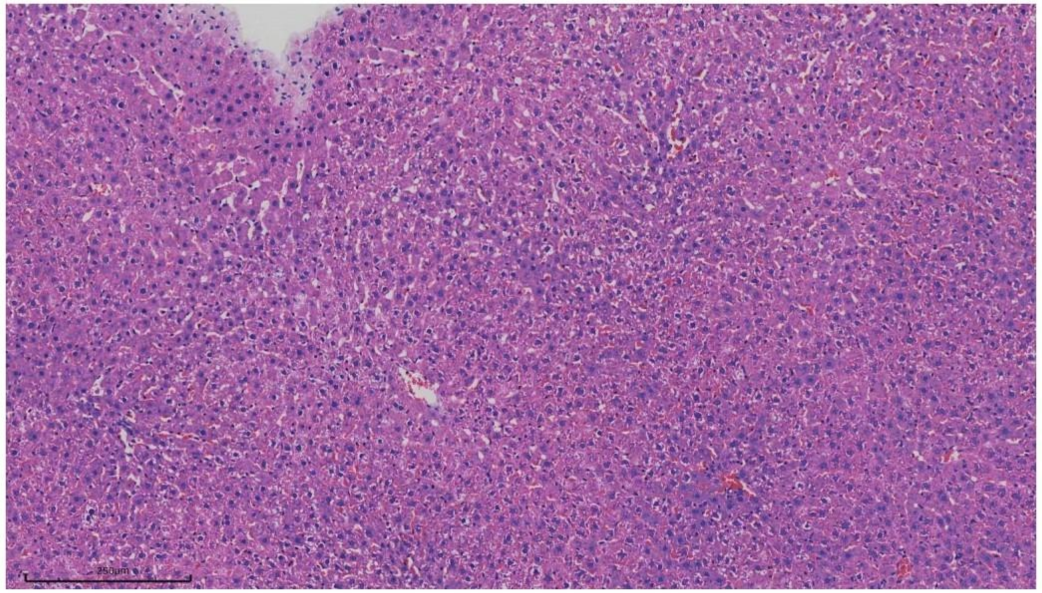

Figure 4m CIA2_10.00X

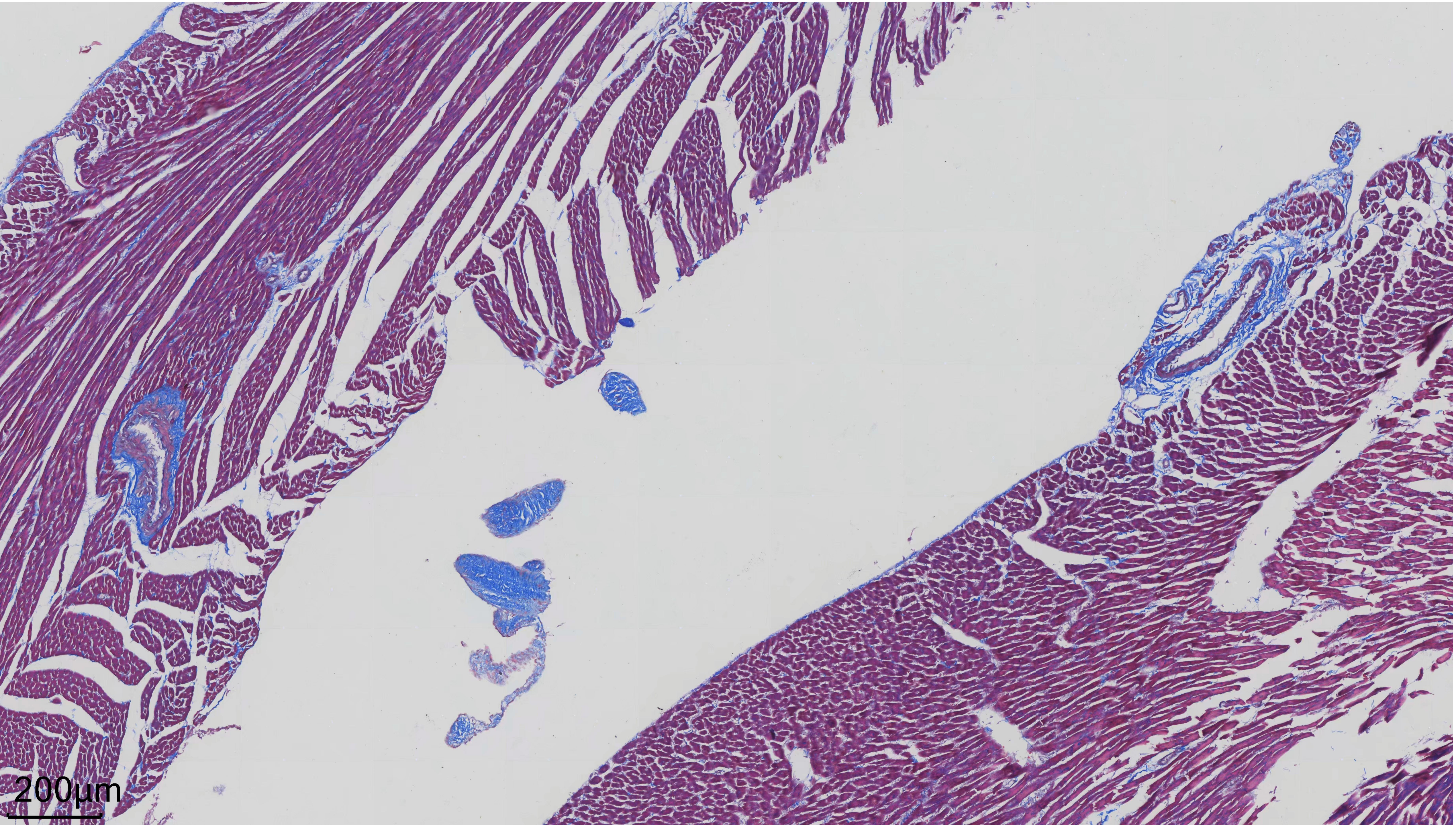

CIA+HFD+IR 4_10.00X

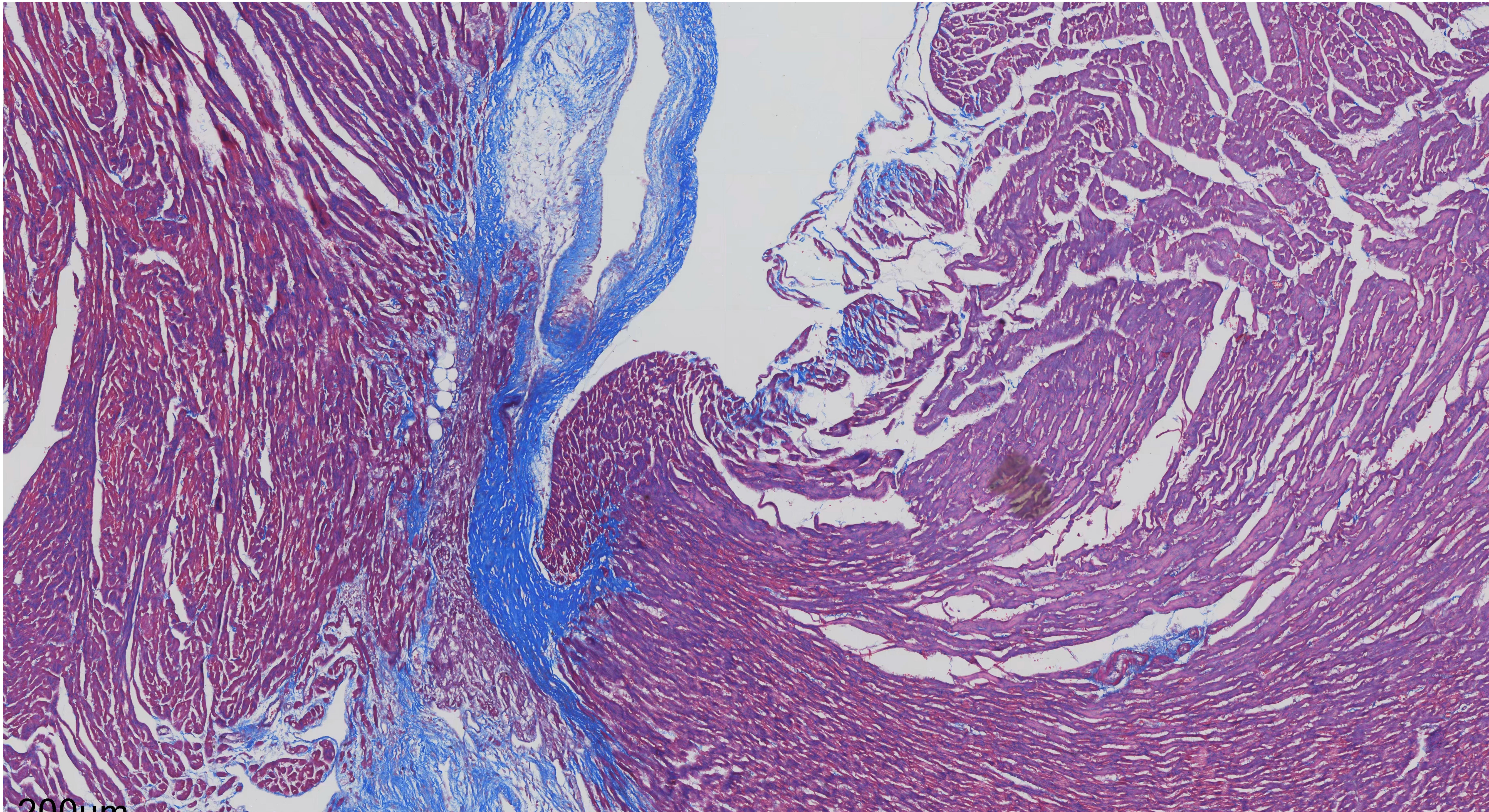

CIA+HFD 1 10.00X

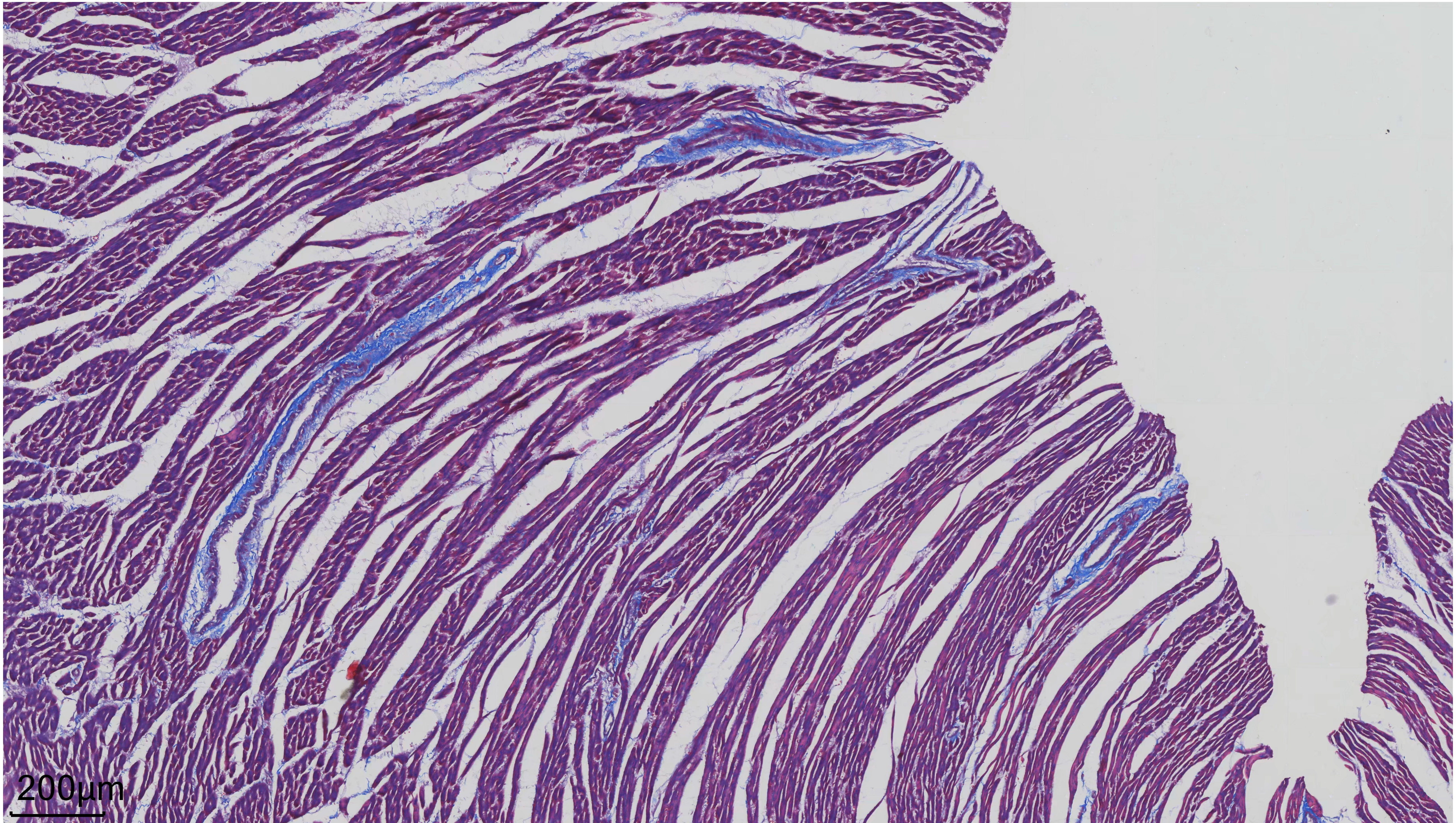

CIA+IR 4_10.00X

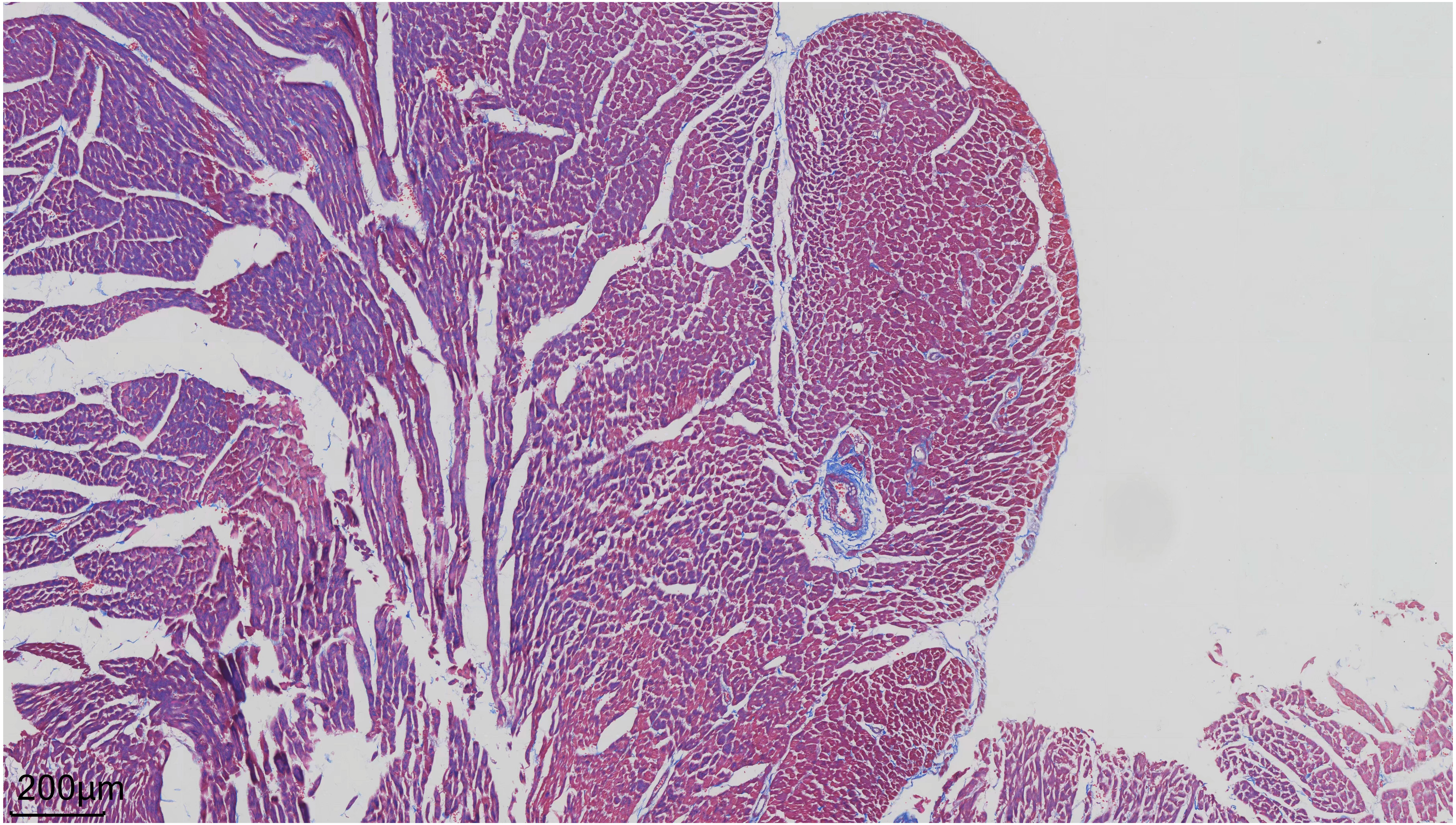

HFD 3_10.00X

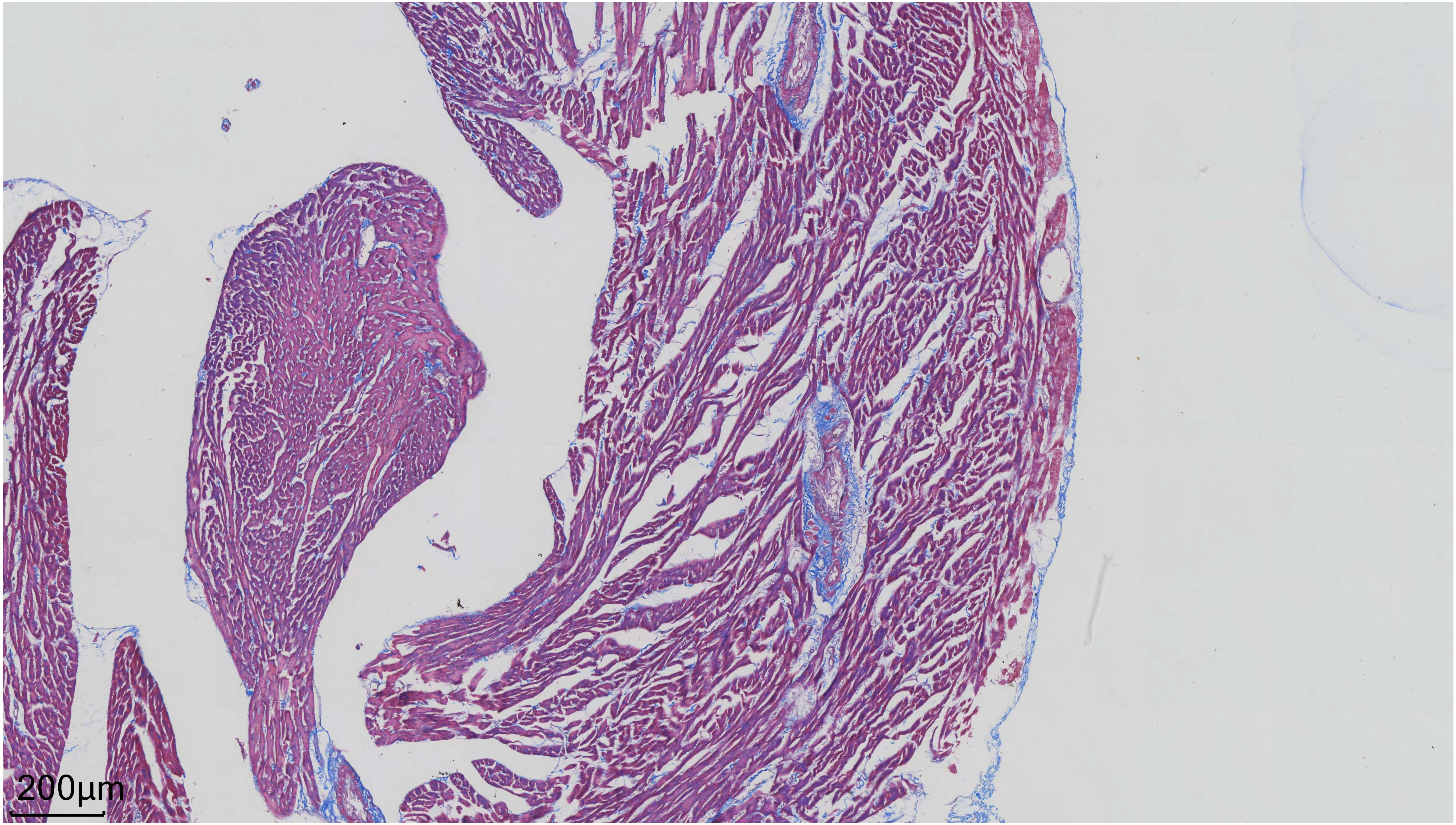

HFD+IR 3_10.00X

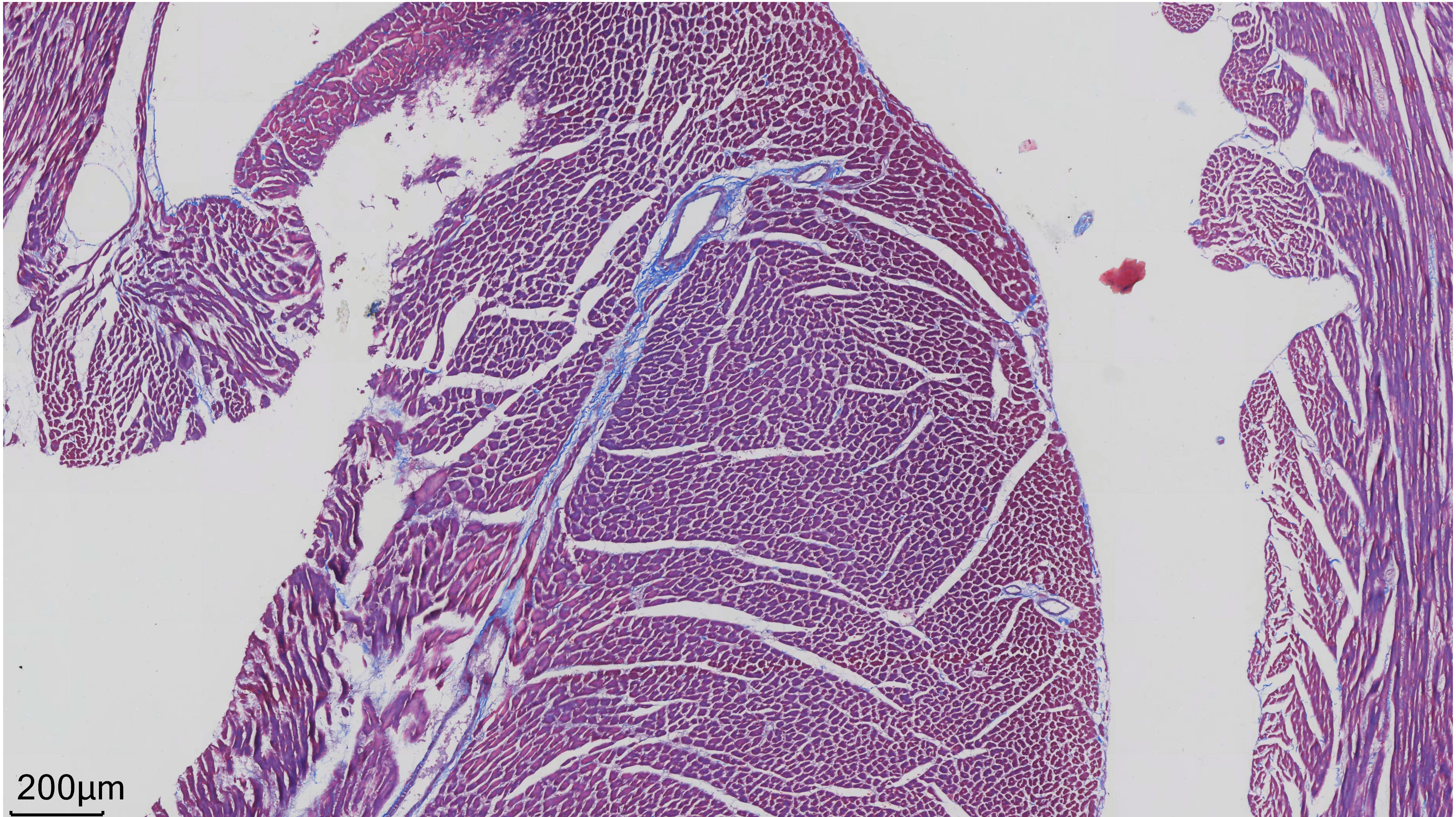

I/R 10.00X

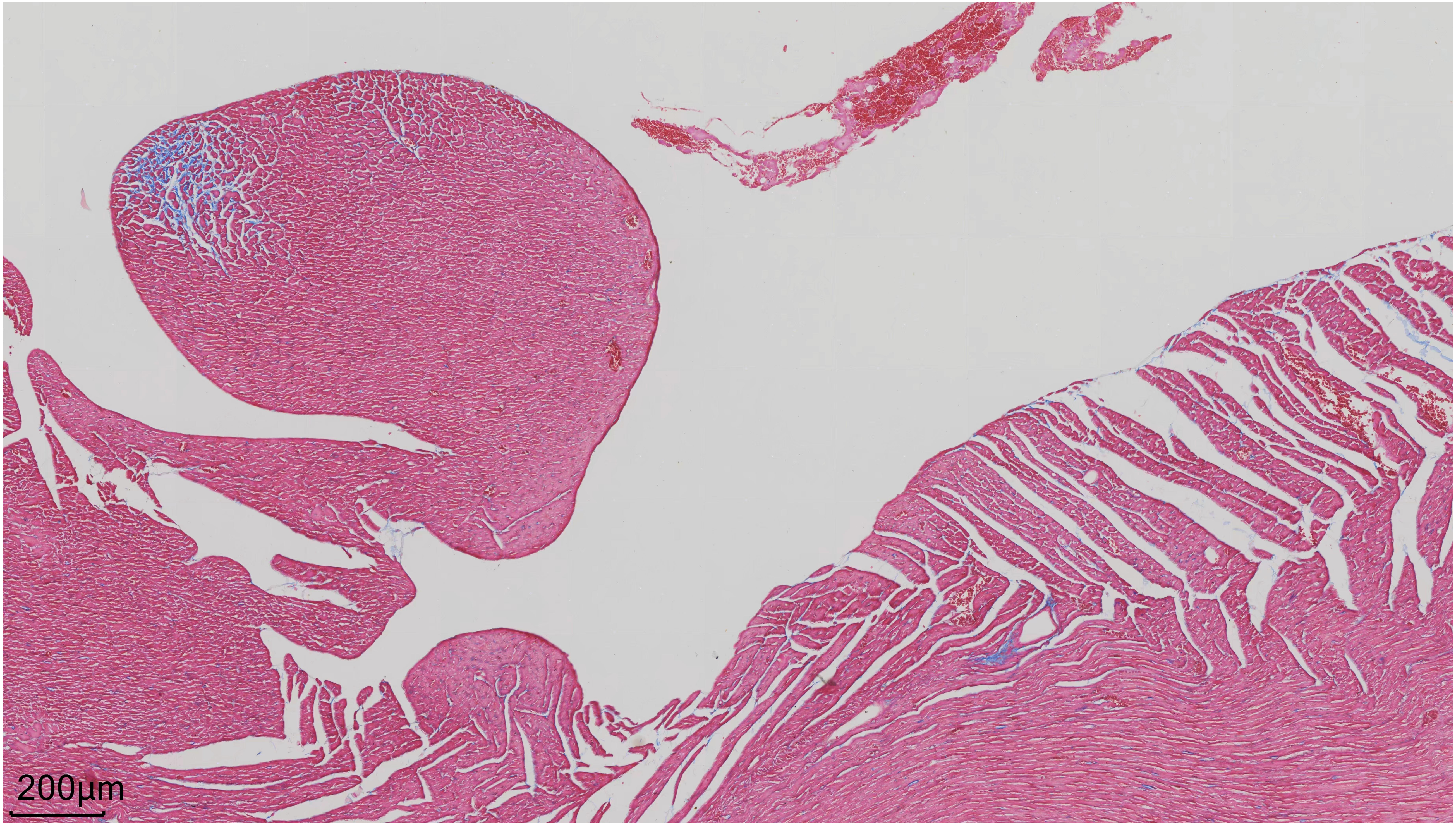

normal_10.00X

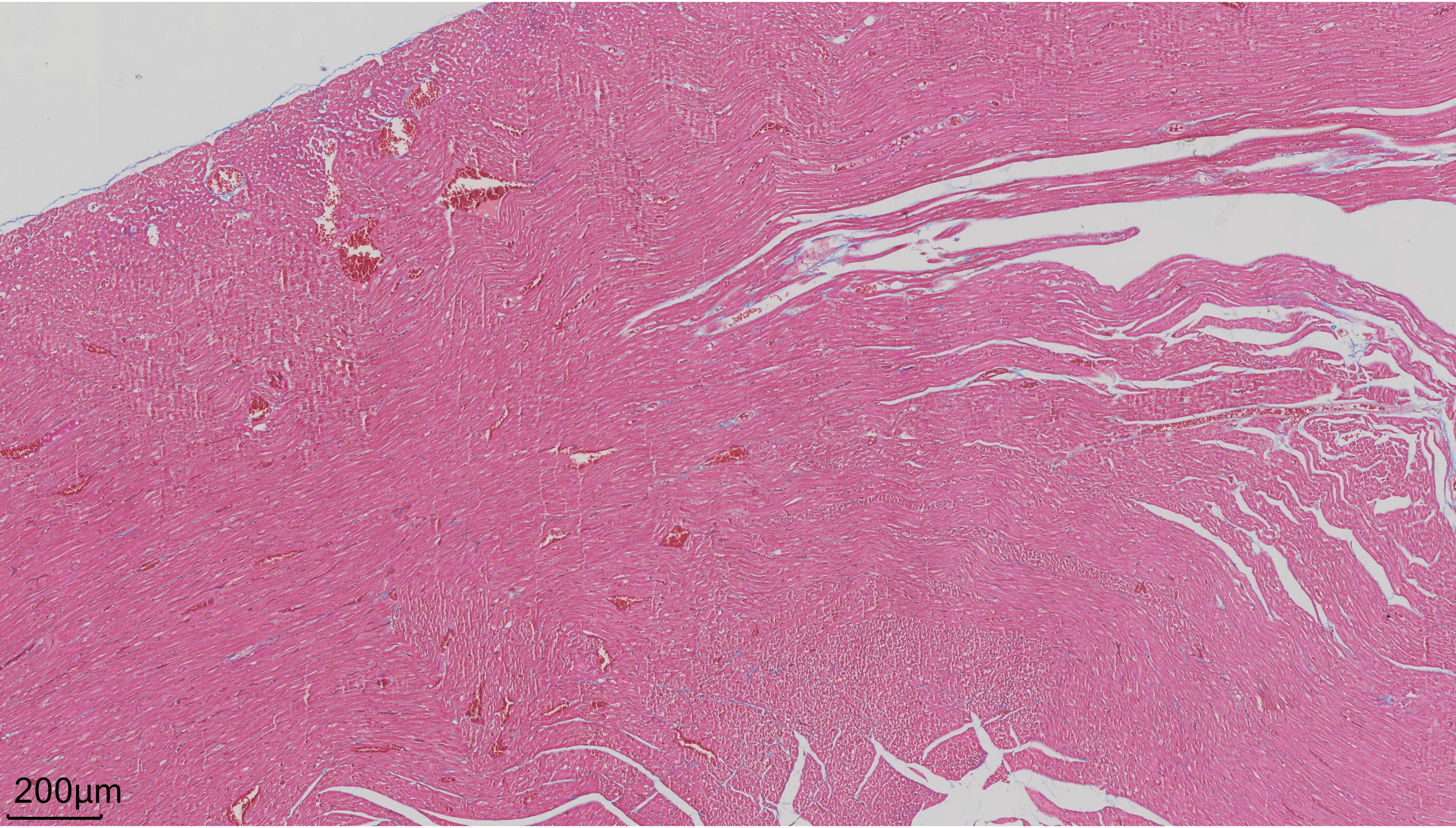

Figure 4 I

CIA1_80.00X

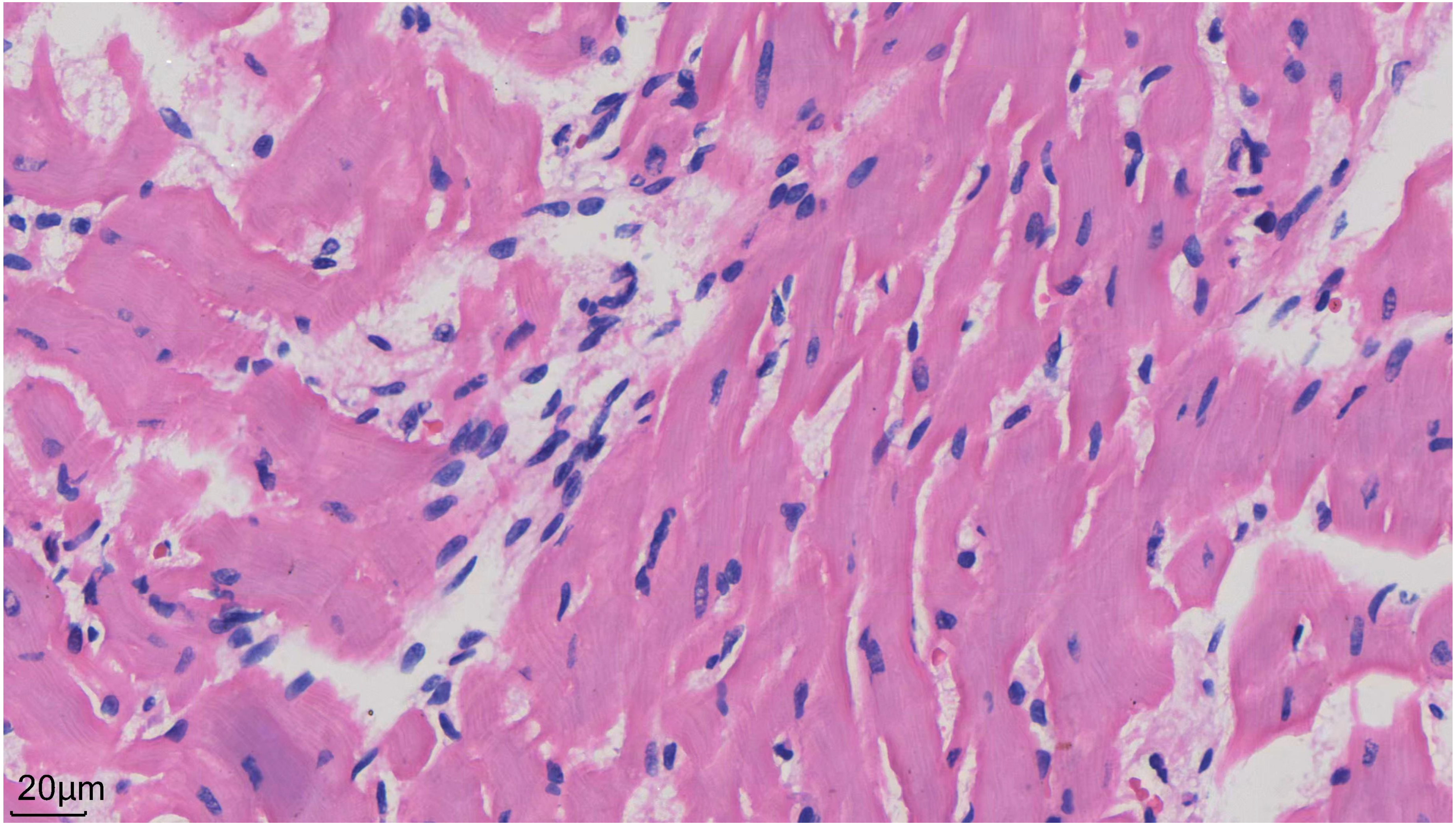

CIA+HFD 1_80.00X

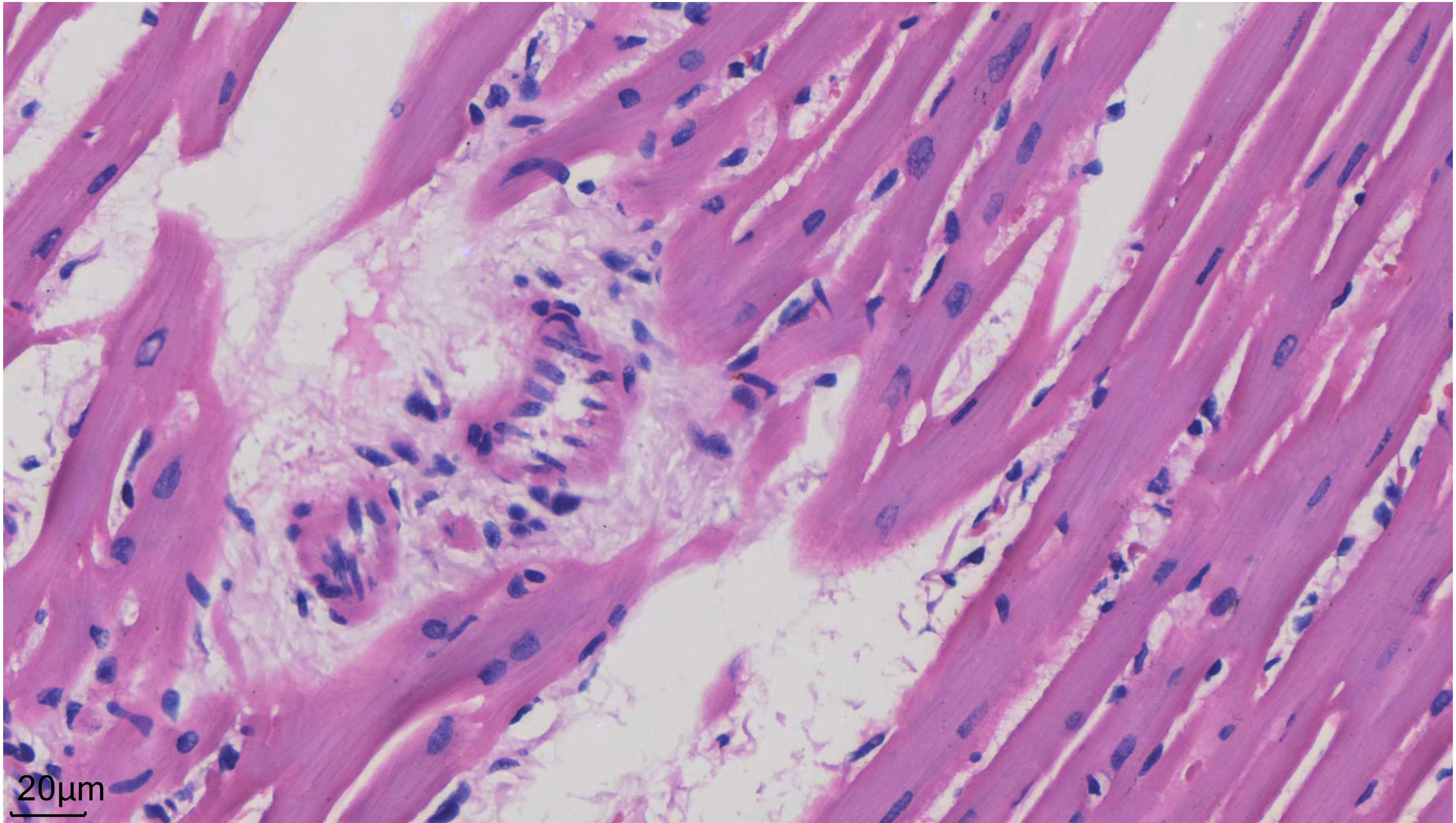

CIA+HFD+IR 2_80.00X

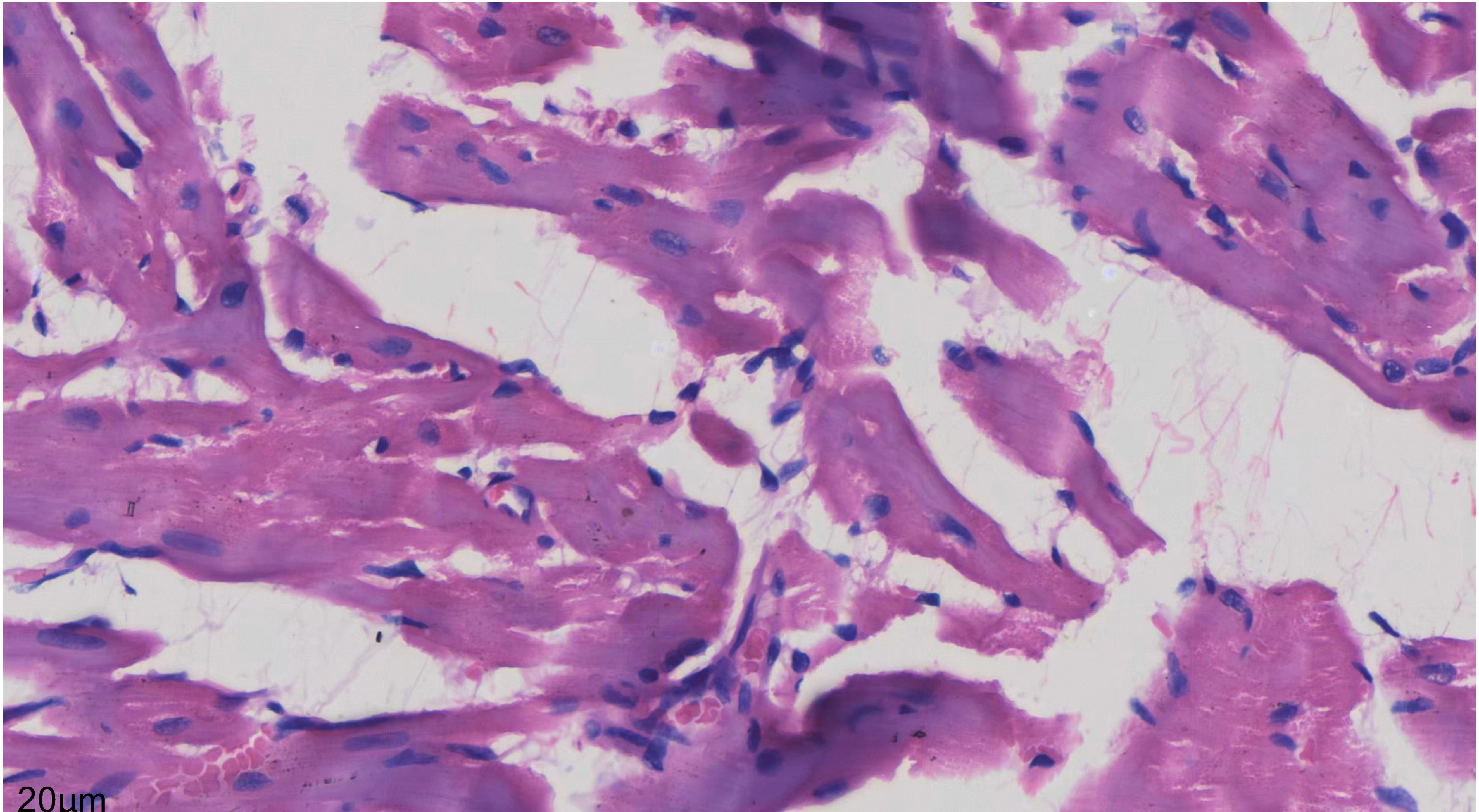

CIA+IR 1_40.00X

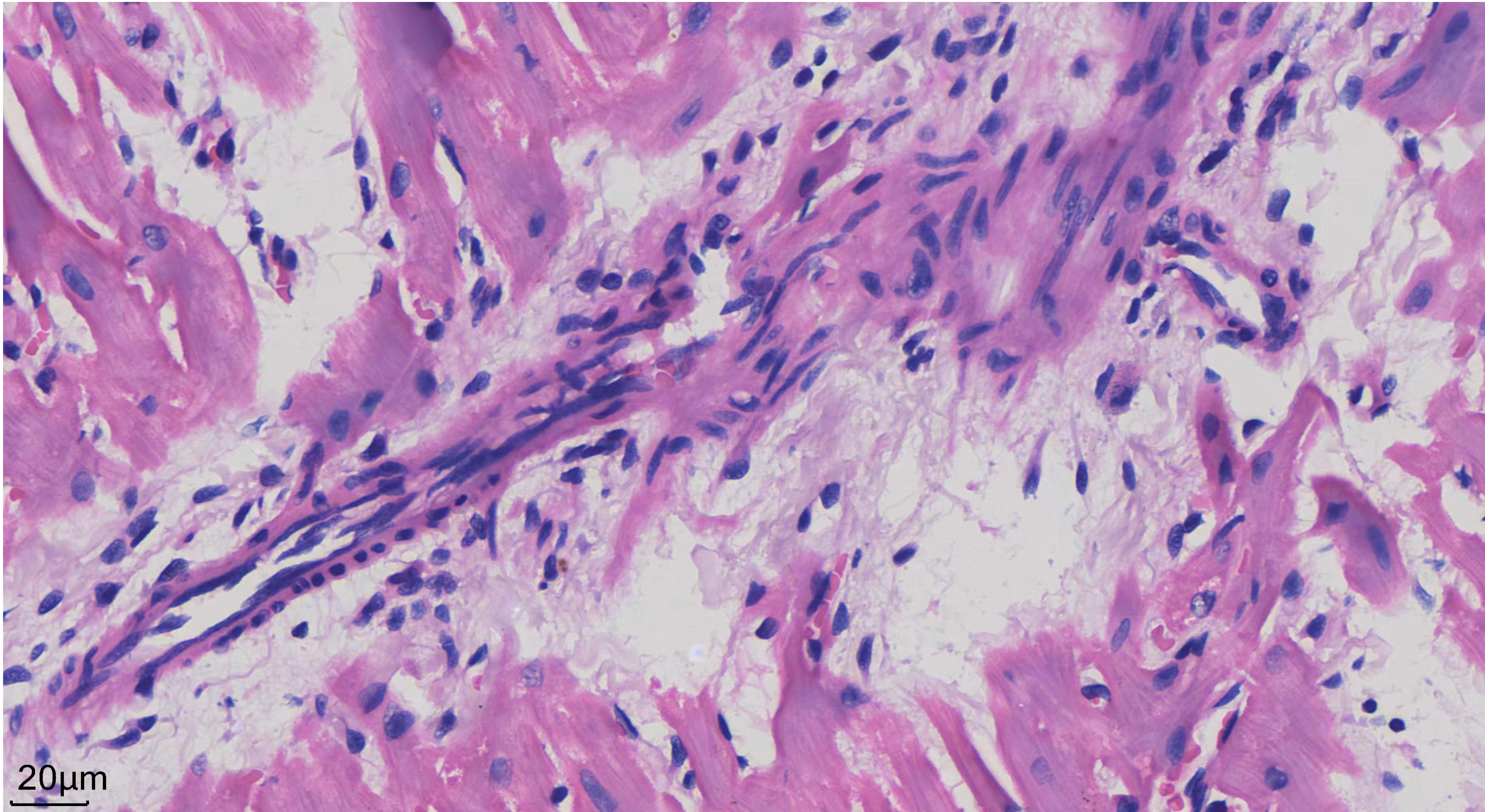

HFD 1_80.00X

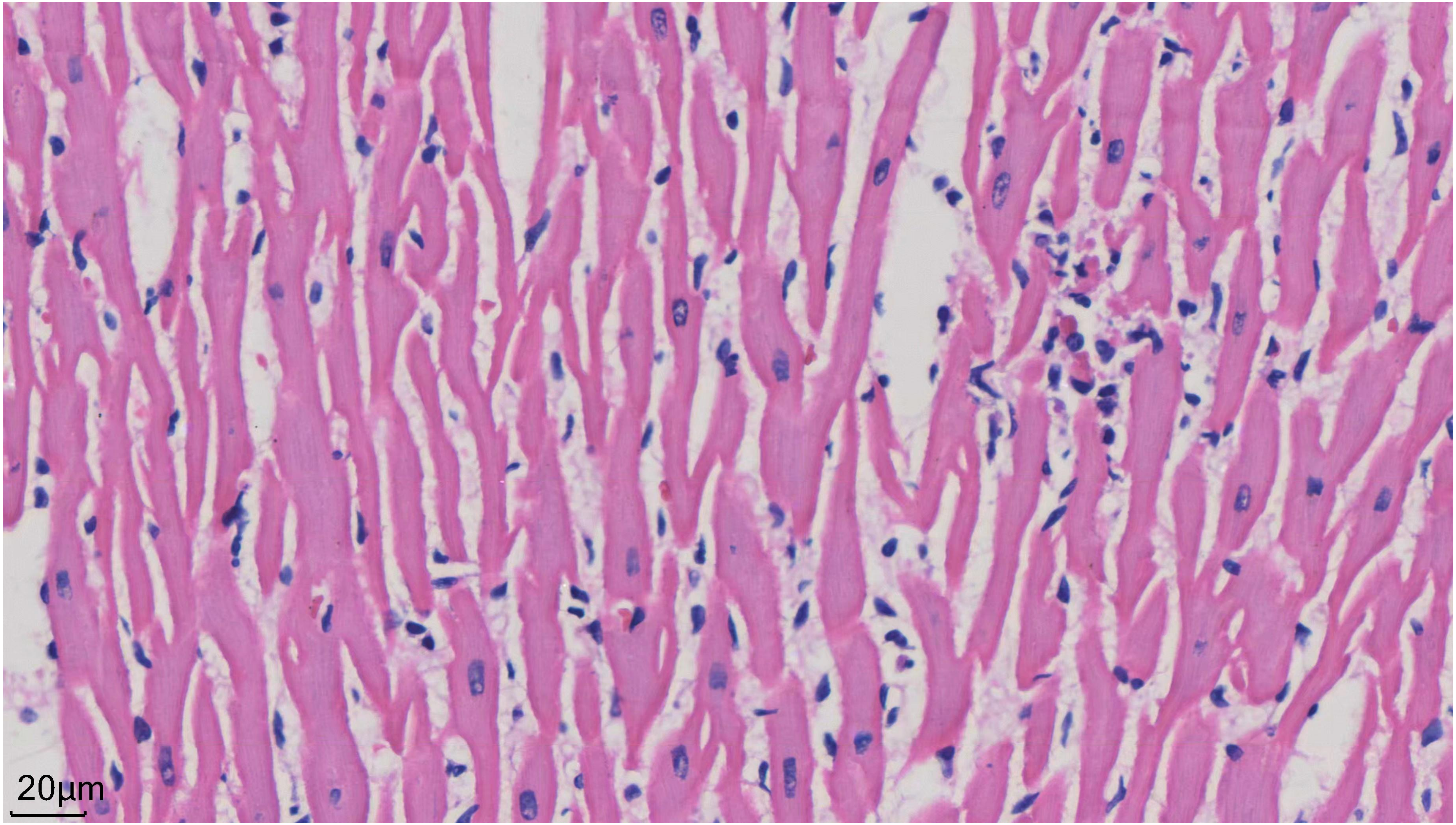

HFD+IR 1_80.00X

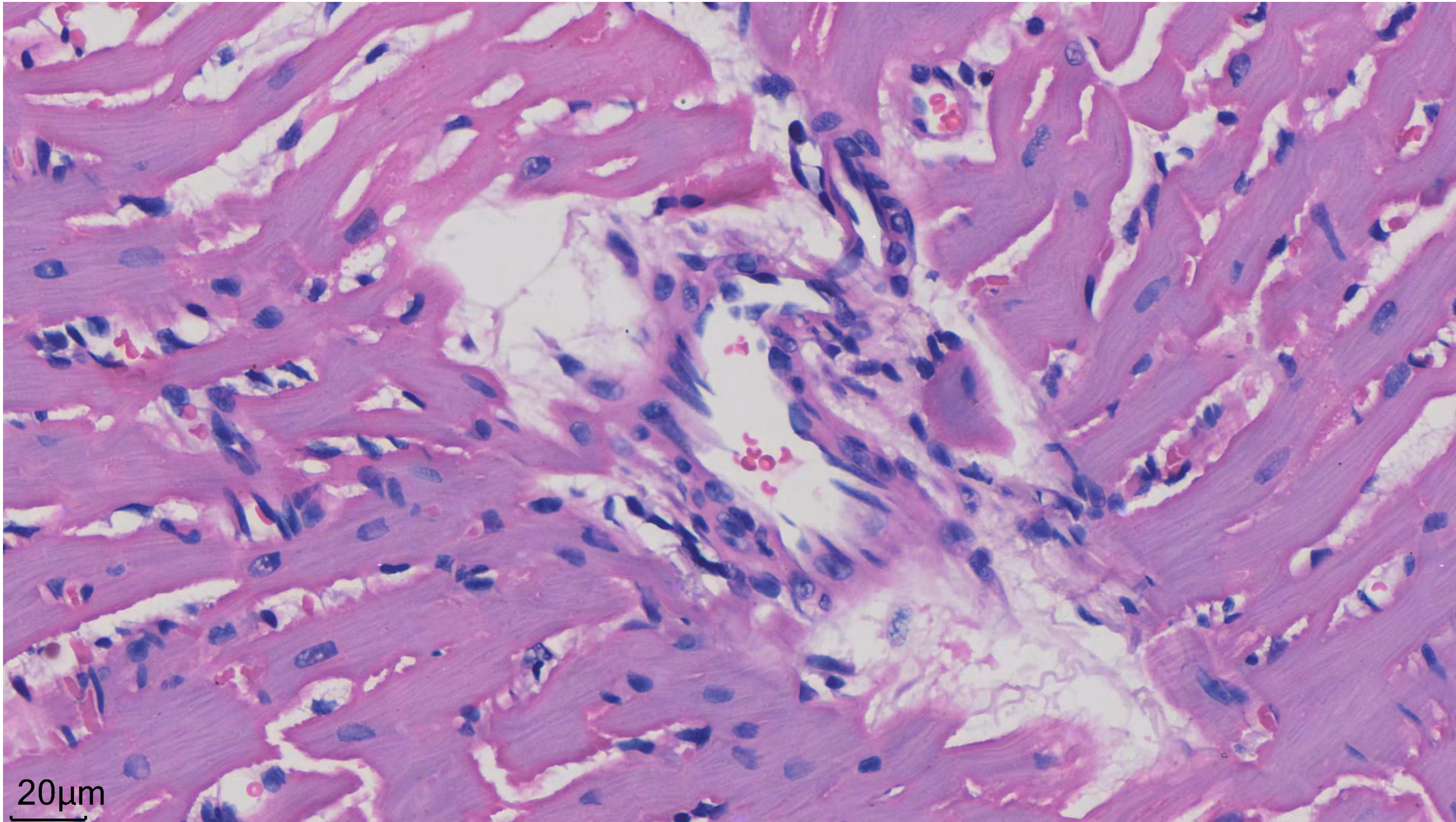

I/R_80.00X

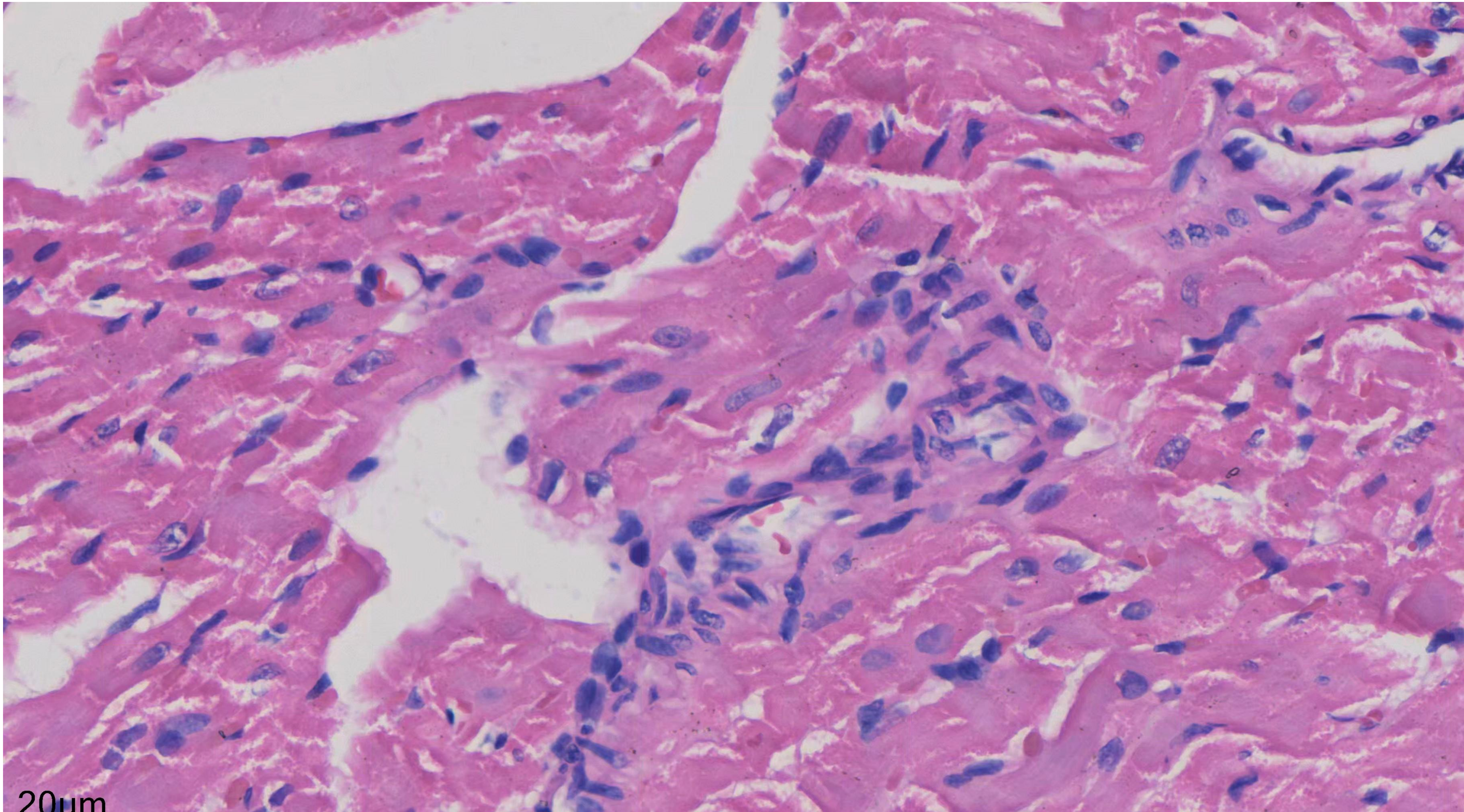

normal_40.00X

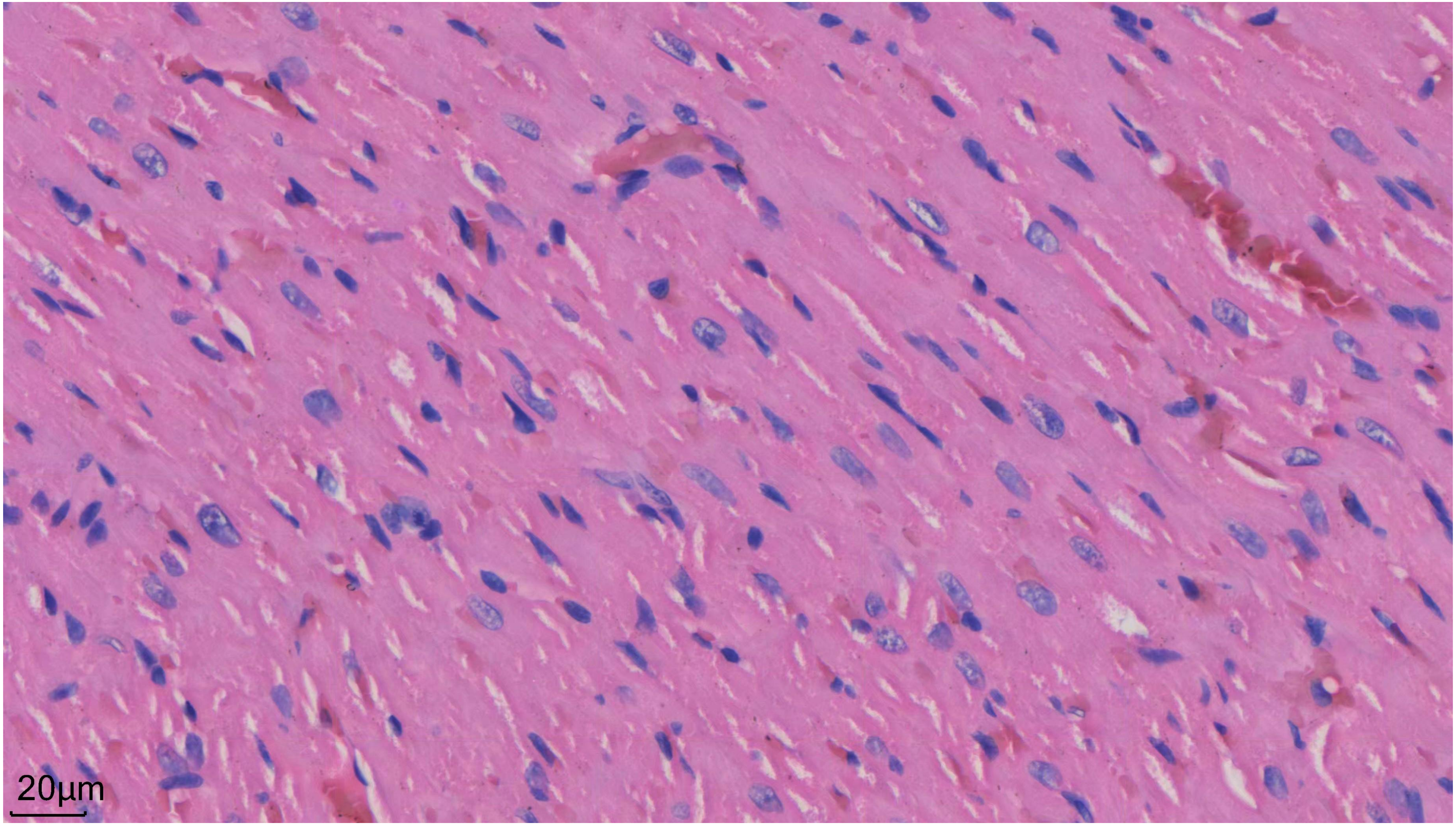

Figure 7 a
CTNT-PTRF

CIA+HFD+IR
2

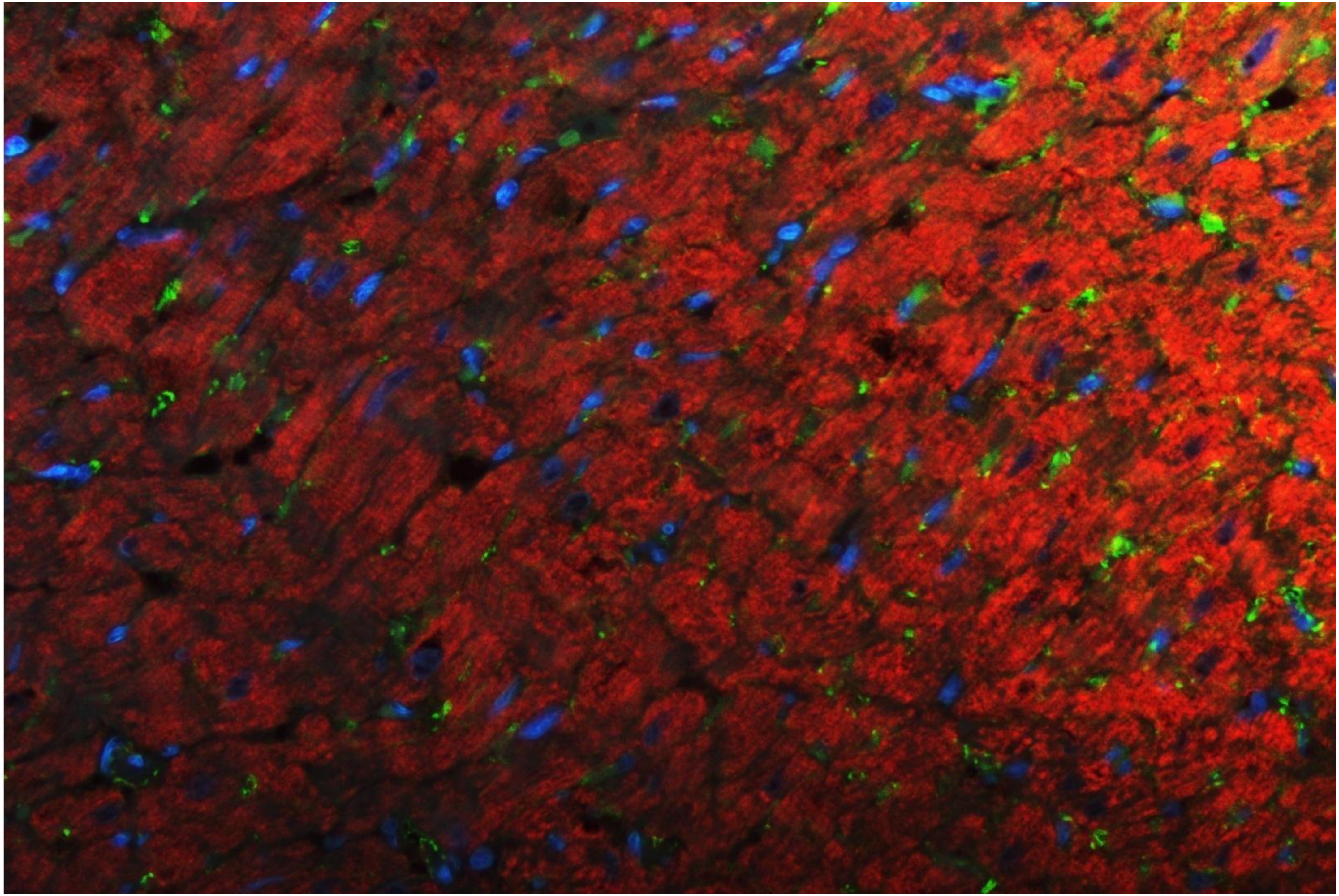

CIA+HFD1

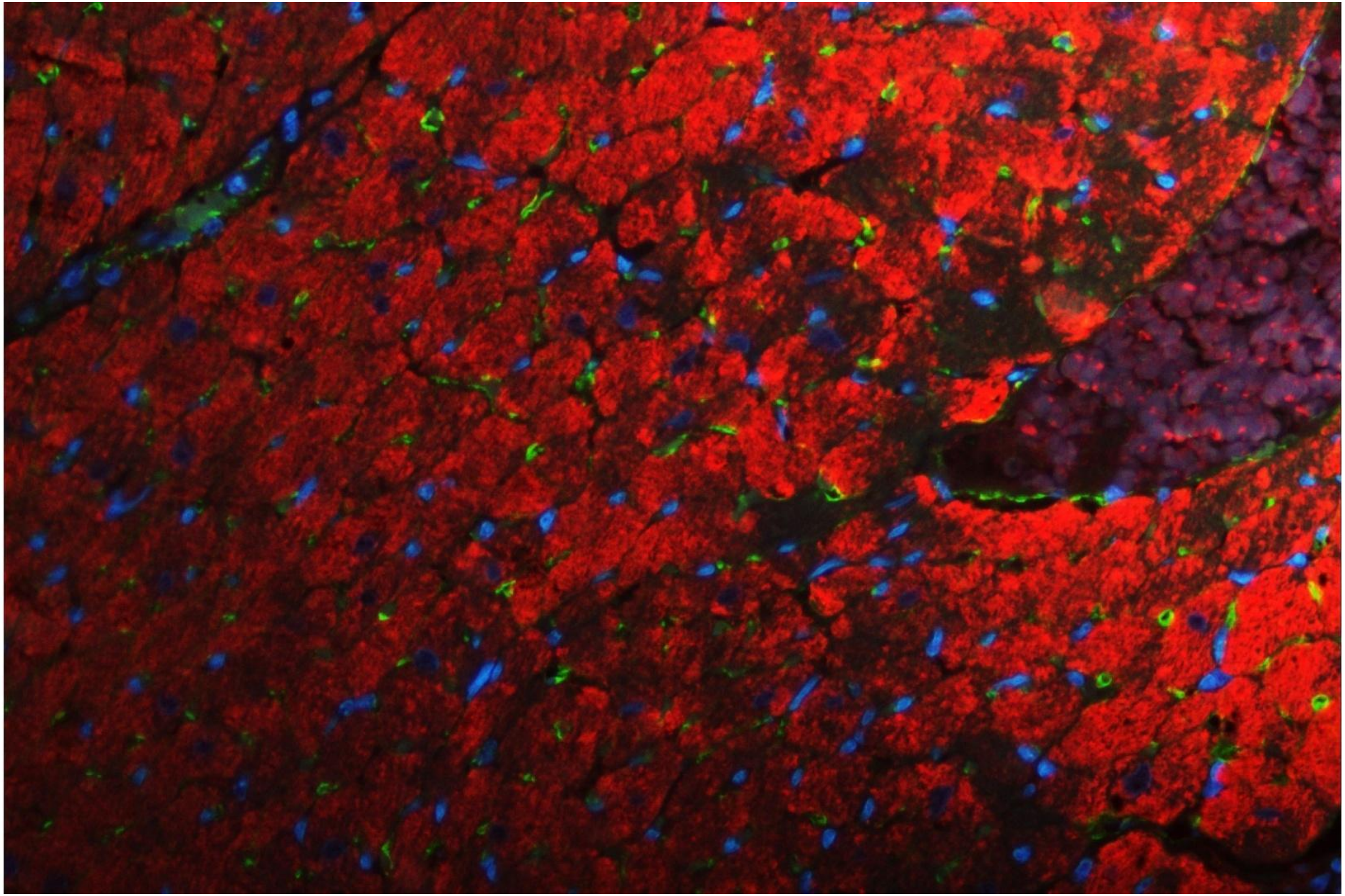

CIA+IR3

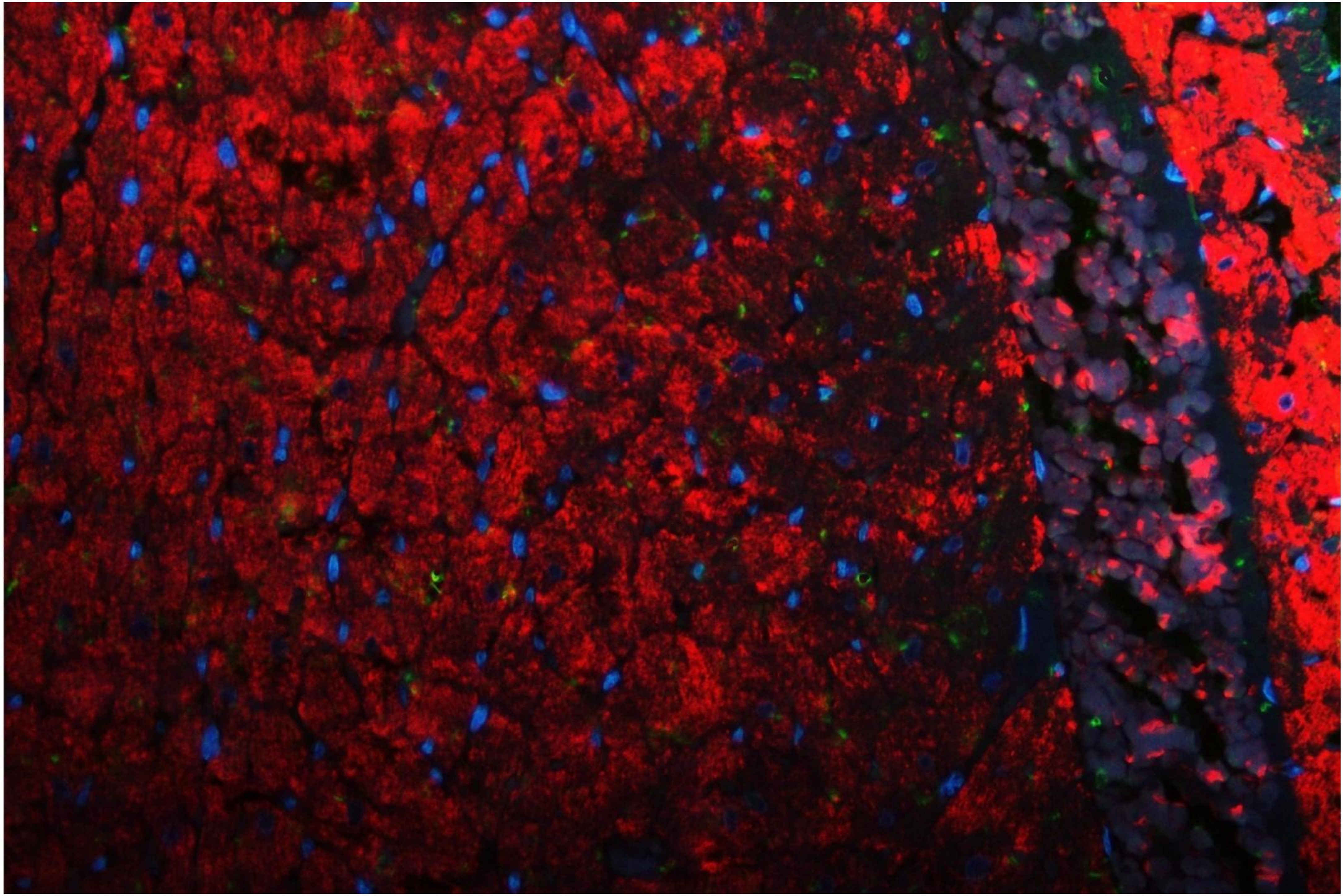

CIA1

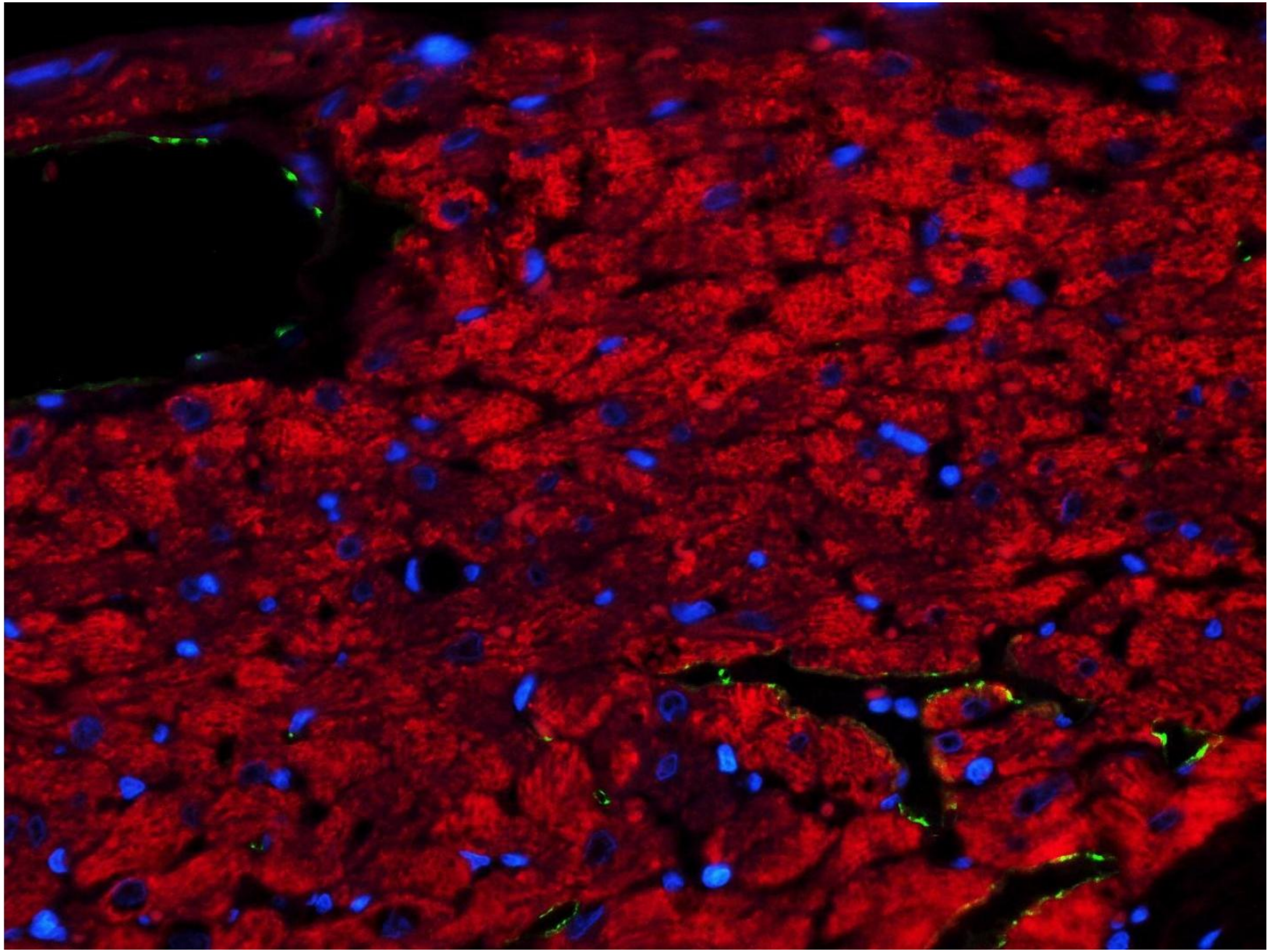

HFD+IR3

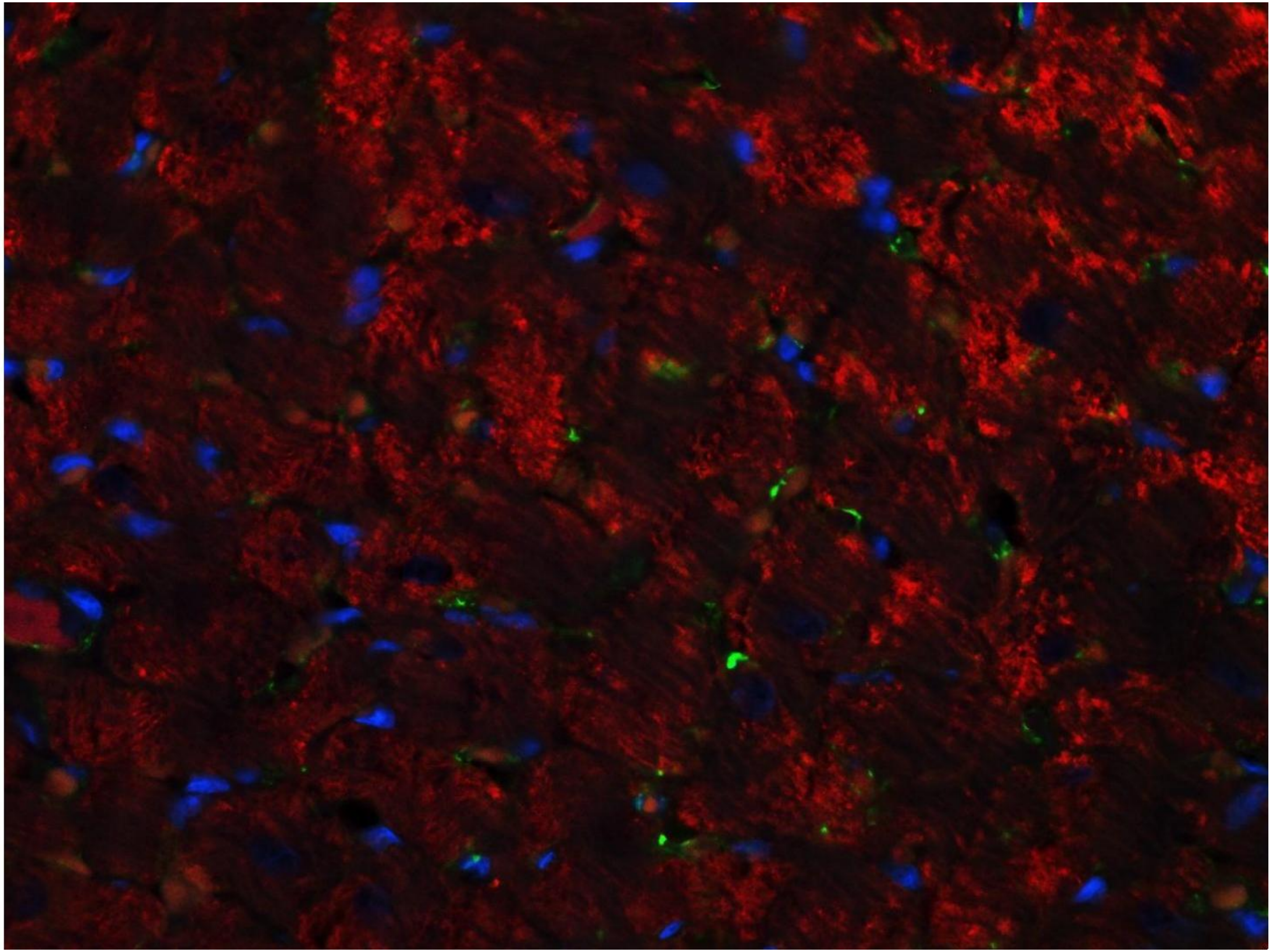

HFD1

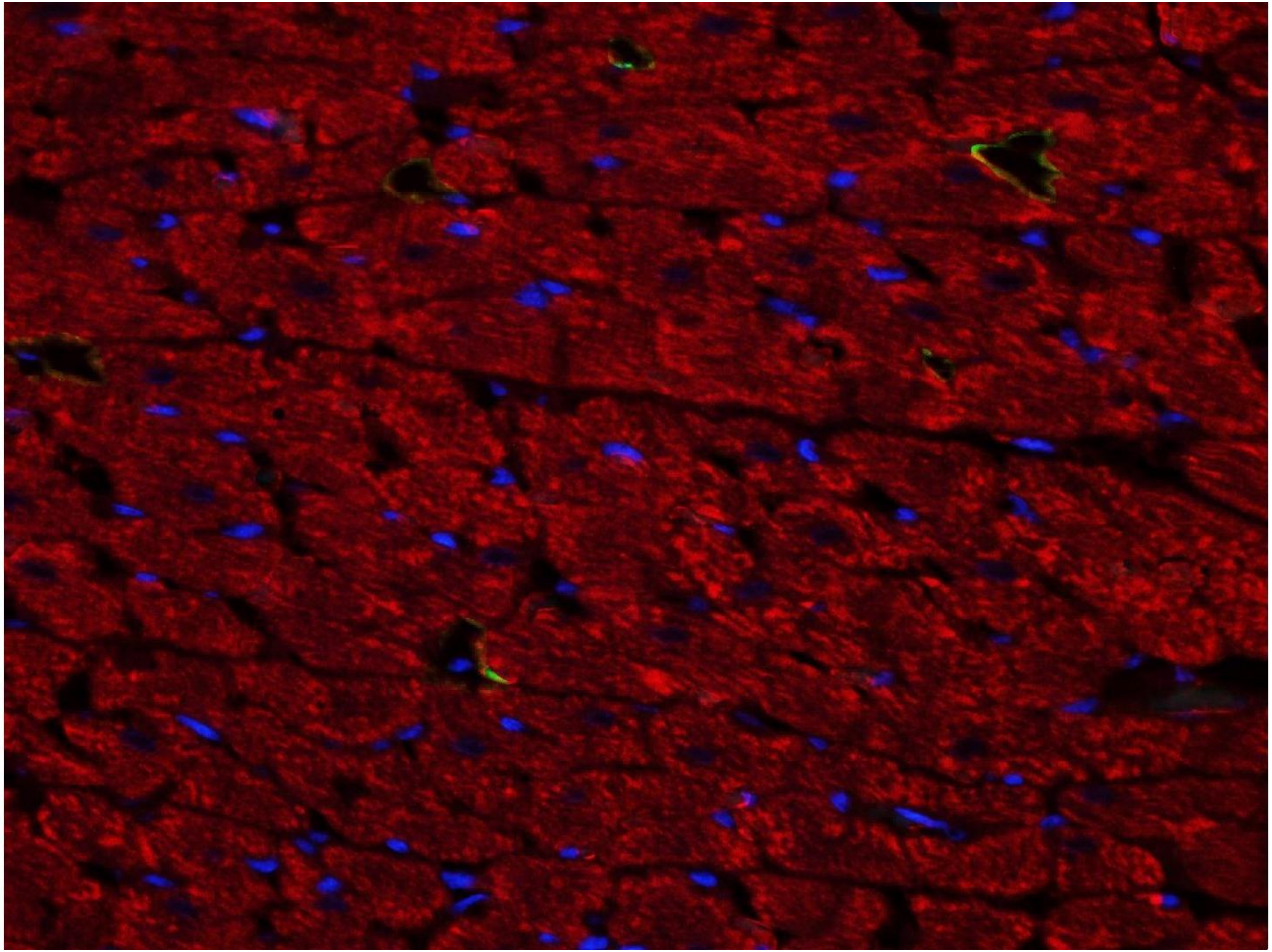

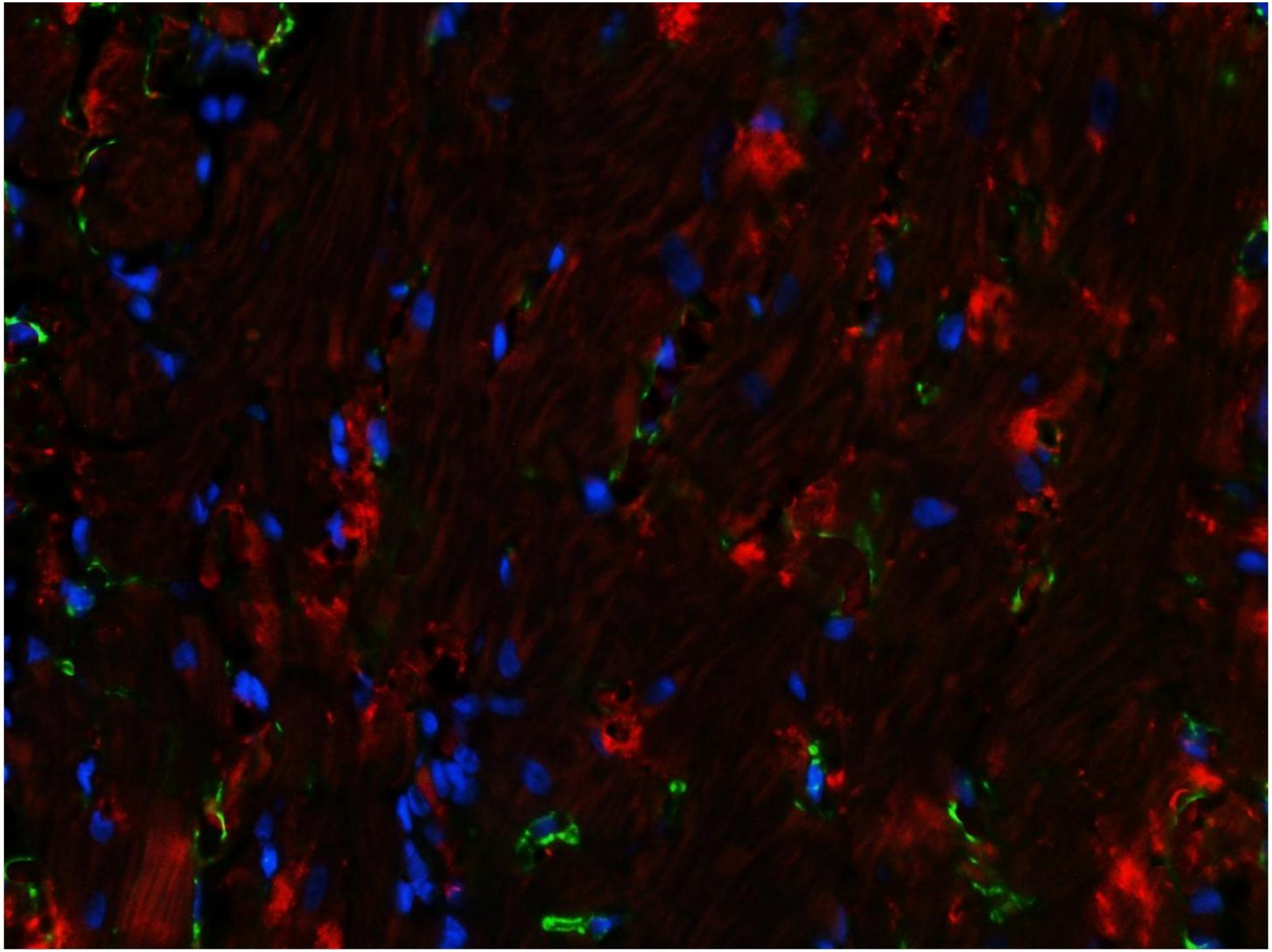

normal

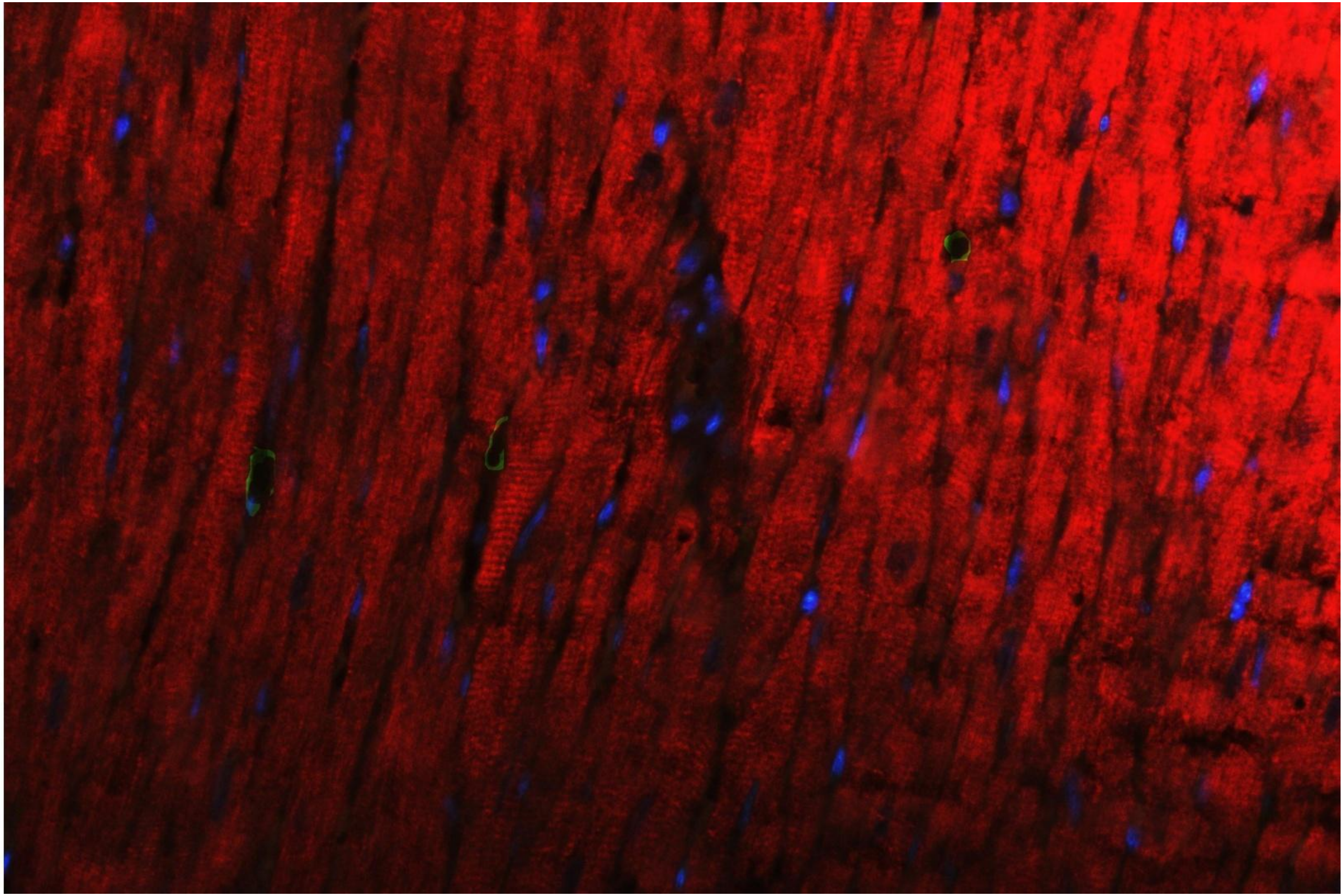

Figure 7 d
CD31-PTRF

CIA+HFD1

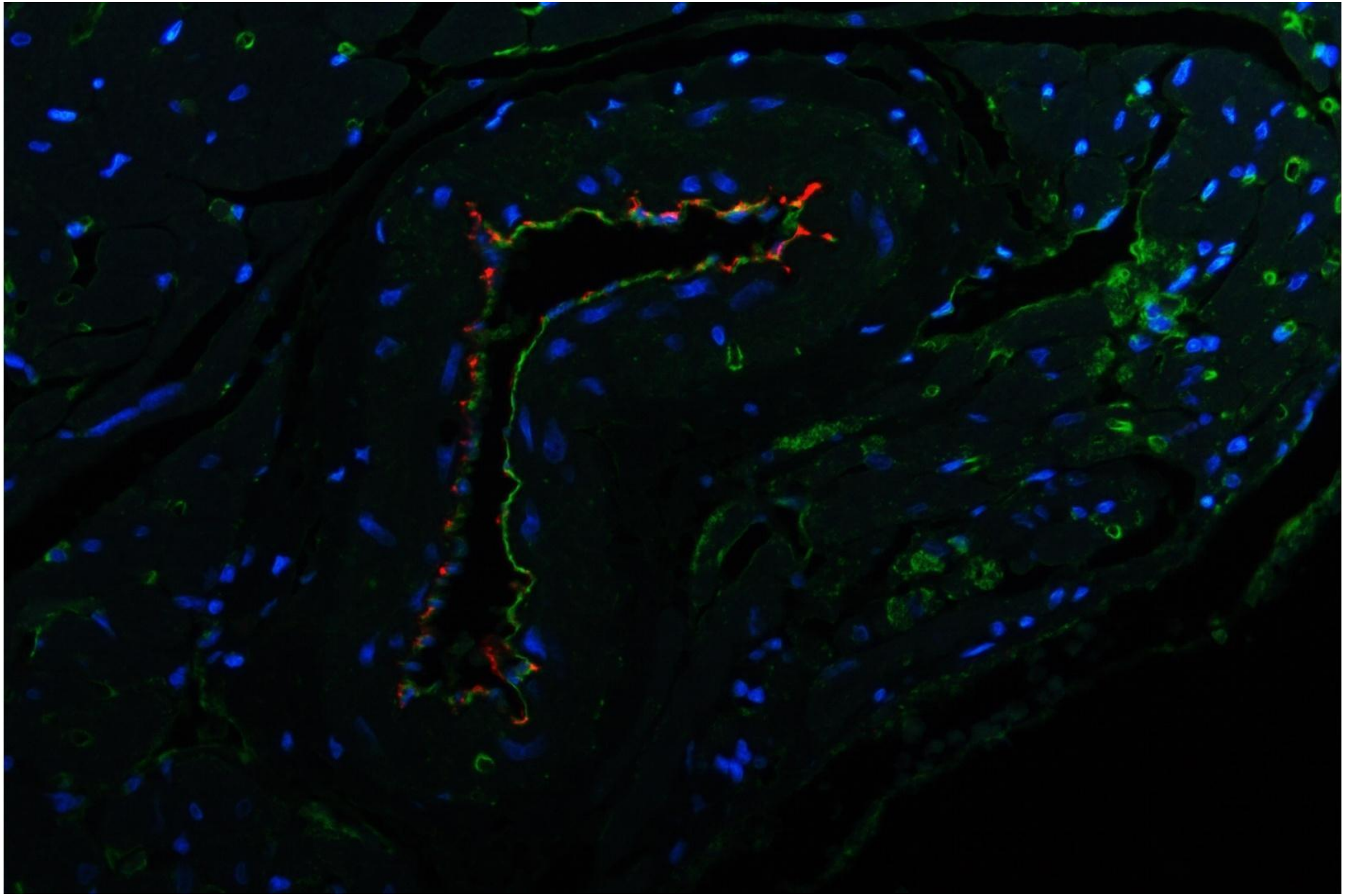

CIA+IR3

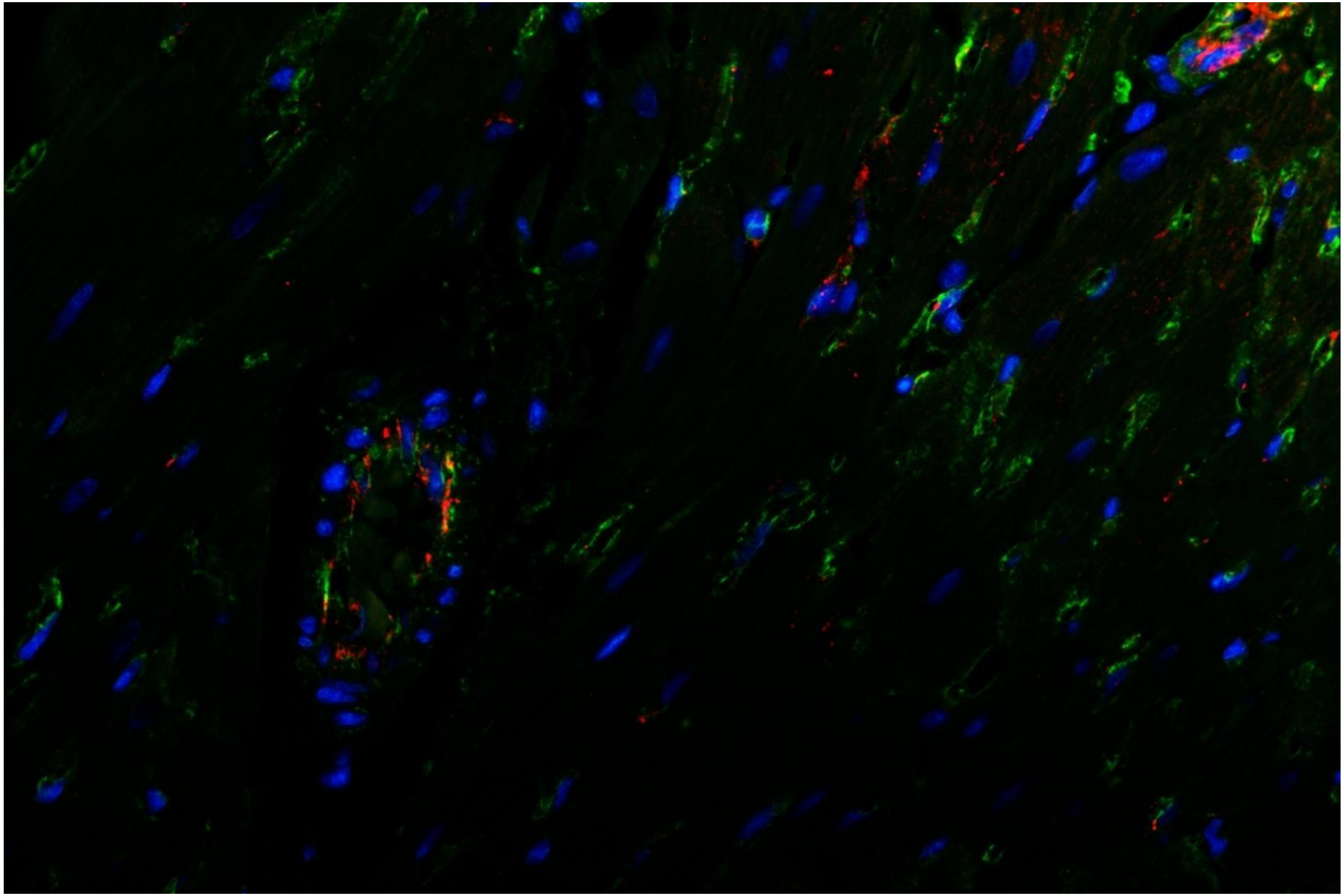

CIA1

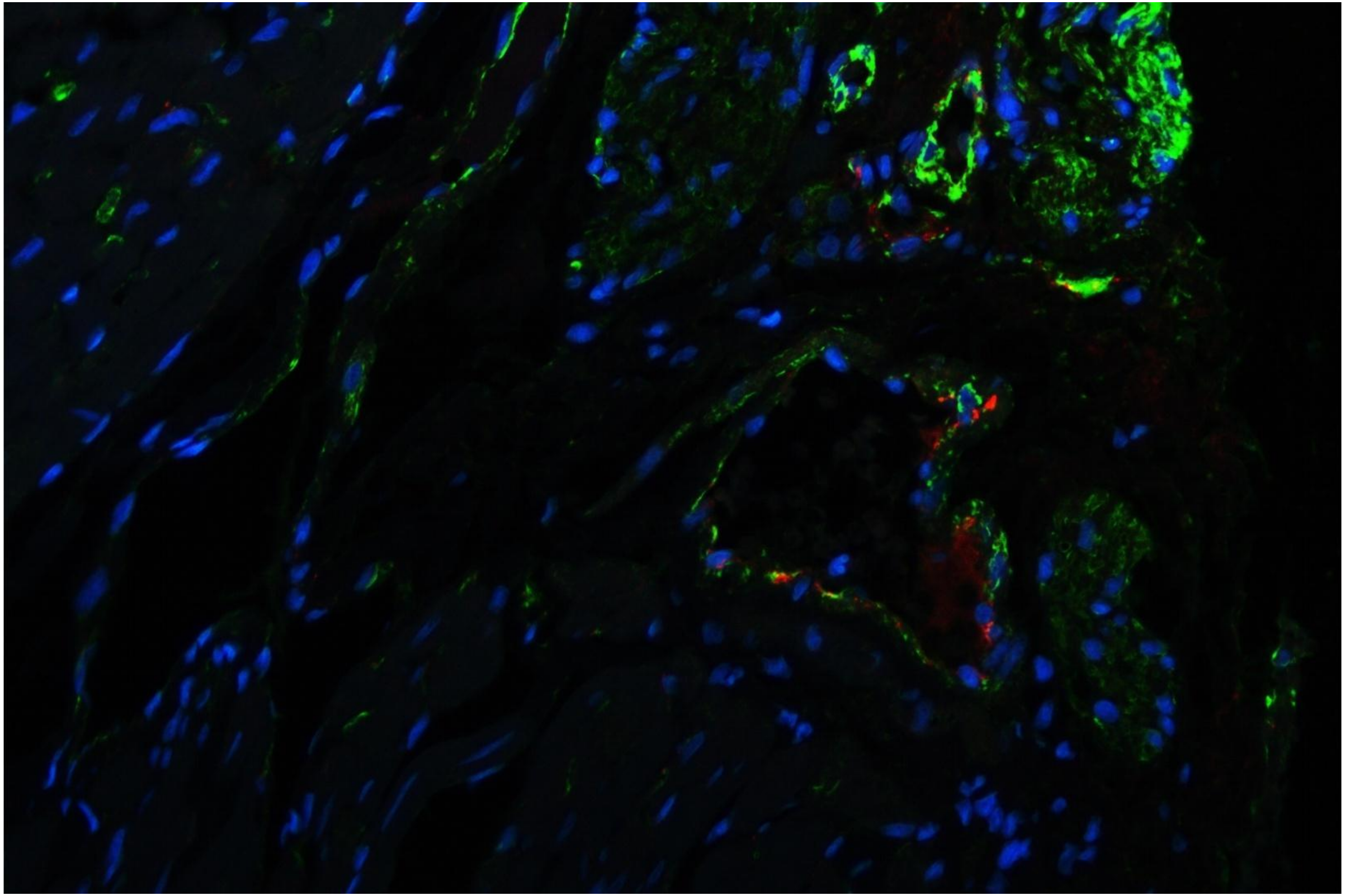

H+C+I

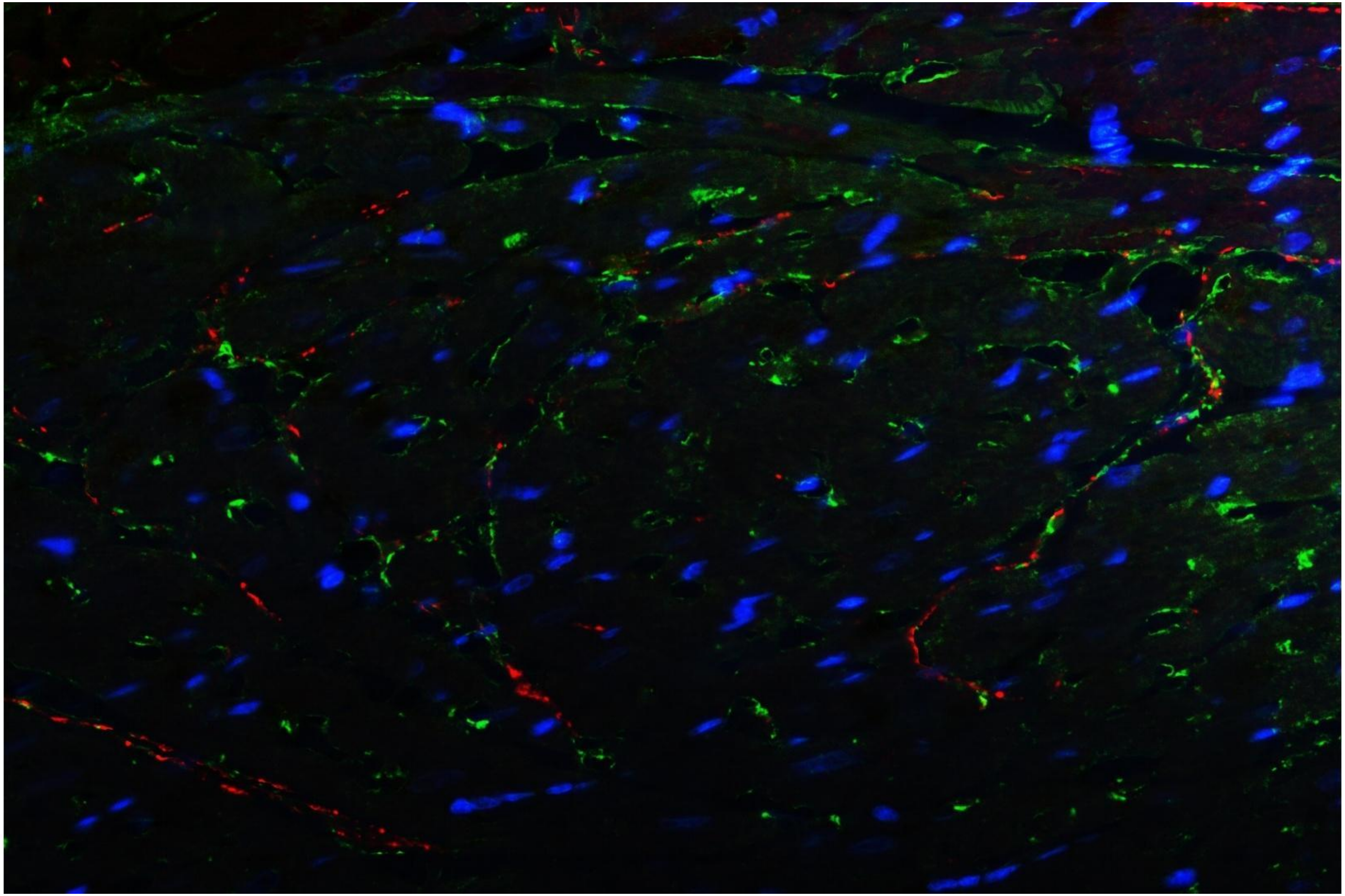

HFD2+Cardiac

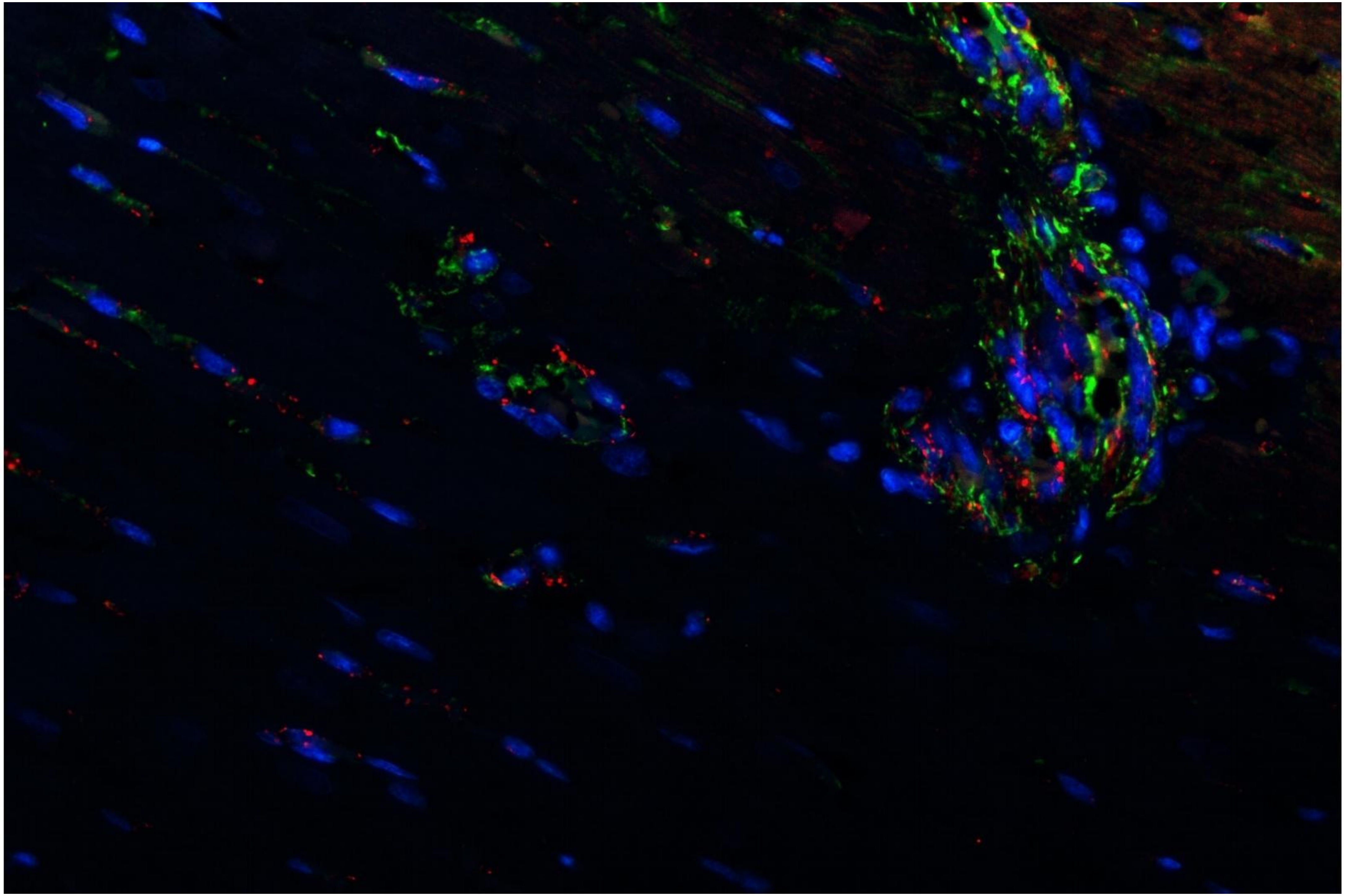

HFD3

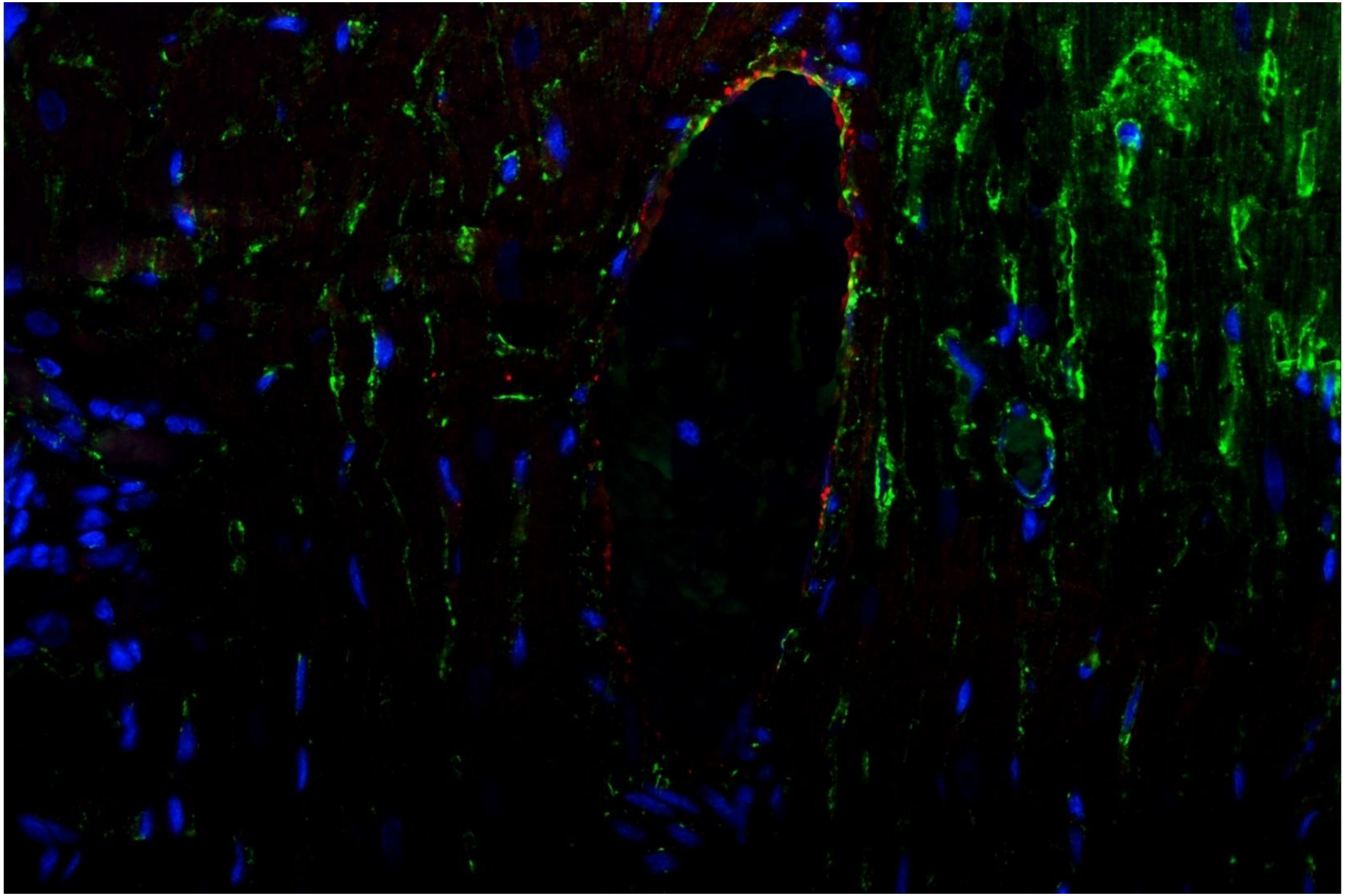

IR4

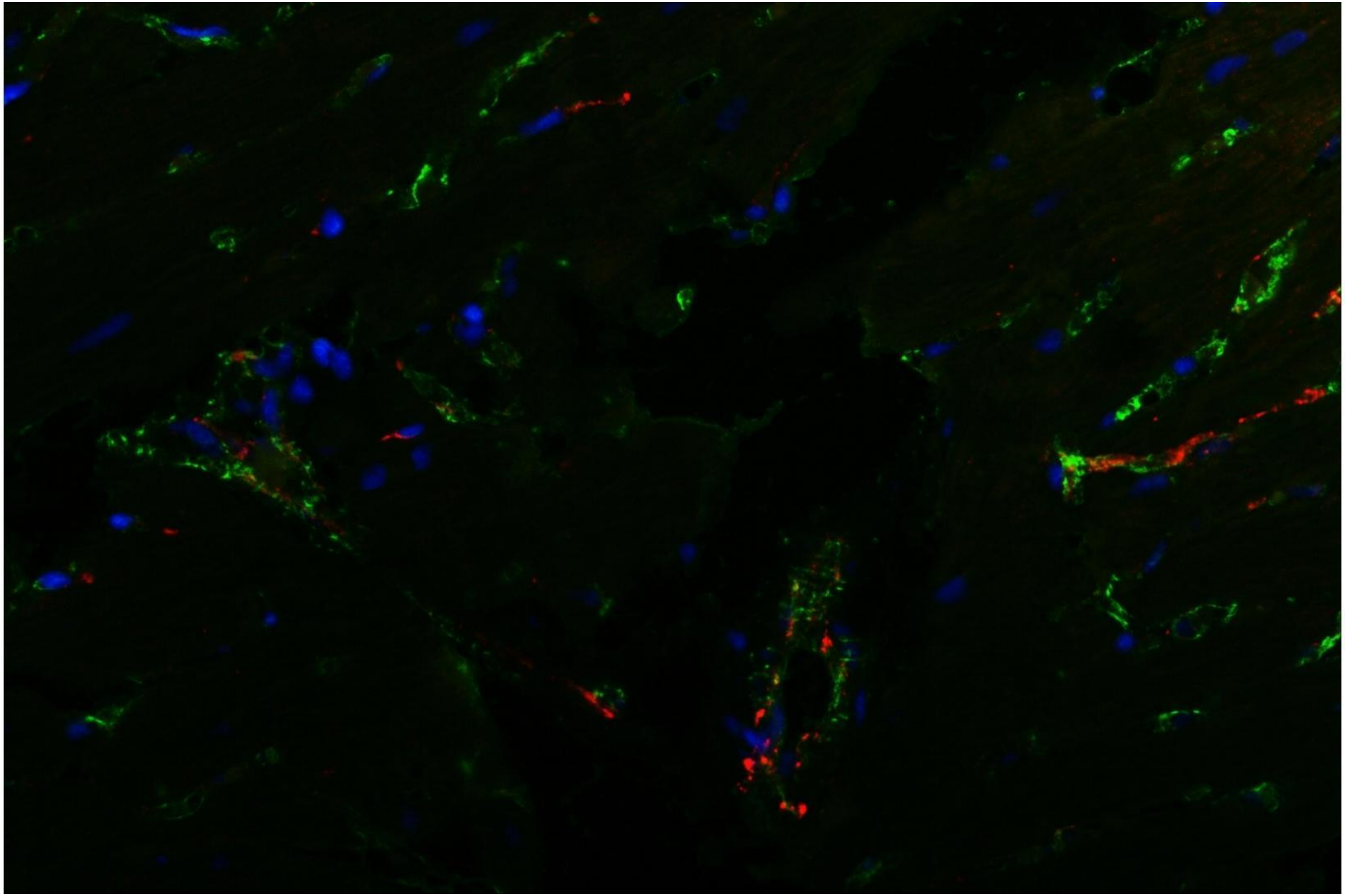

Normal 1

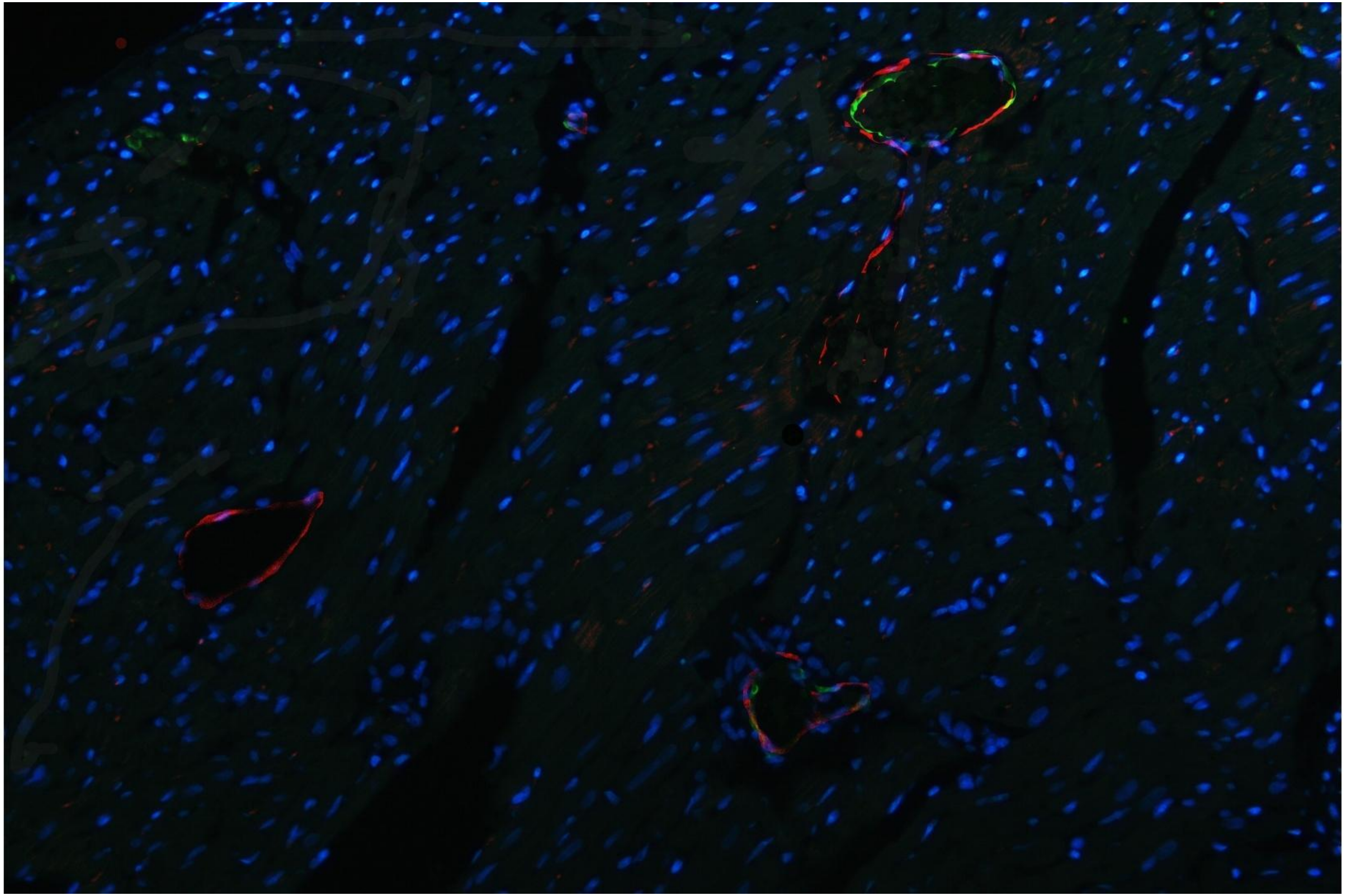

Figure 7
TLR4+PTRF

Cardiac+HFD

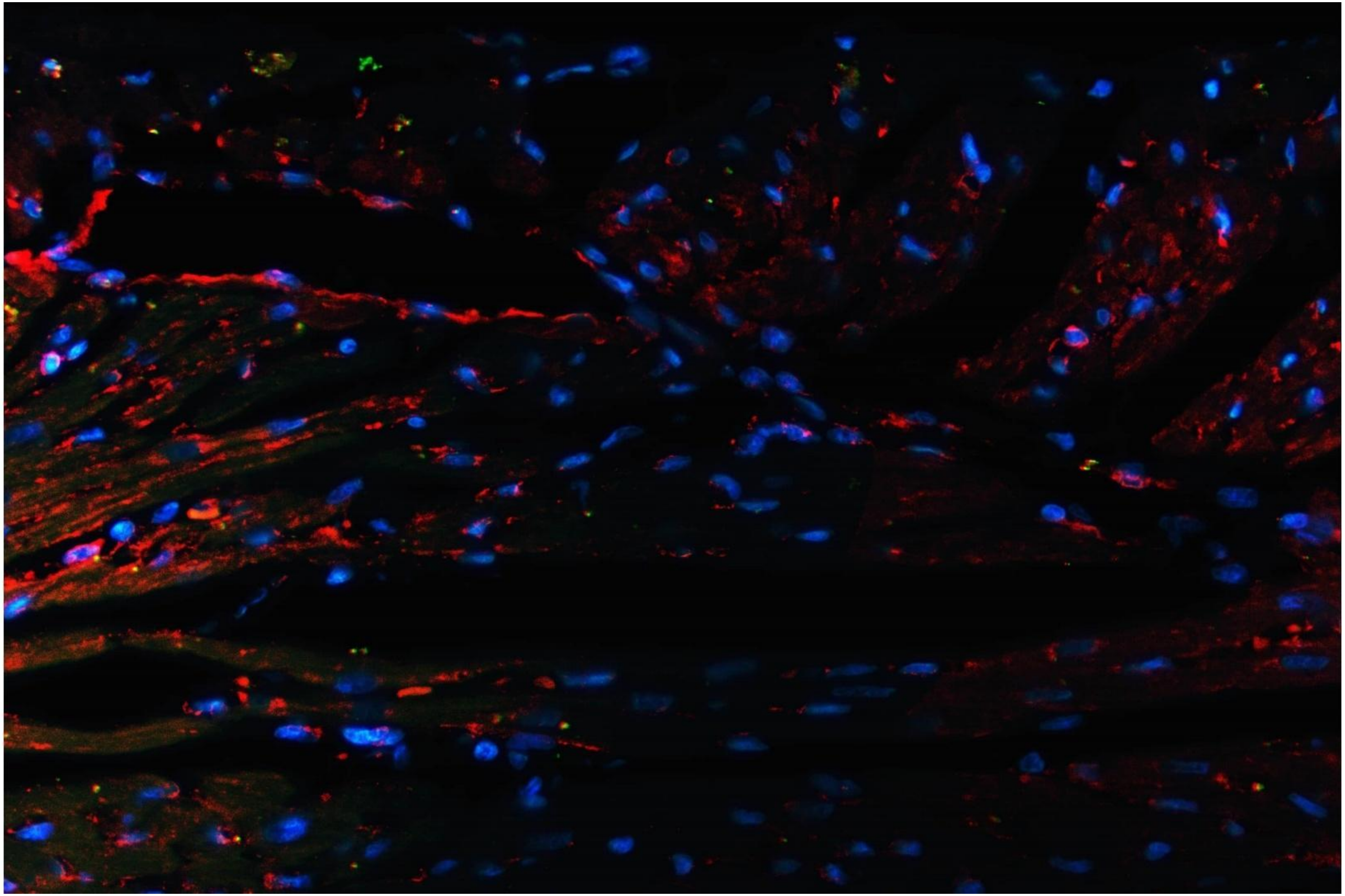

Cardiac IR+
CIA

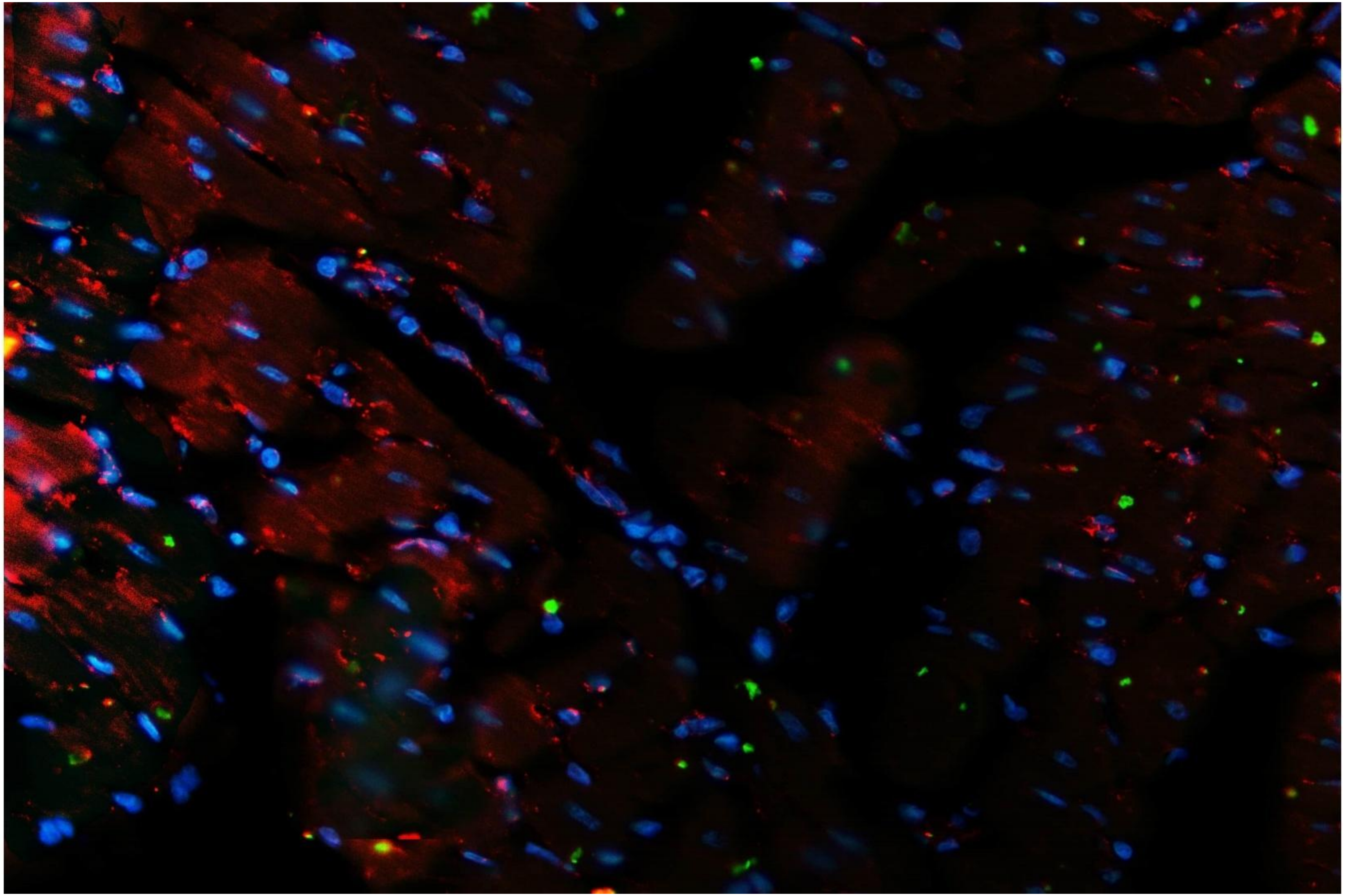

Cardiac IR

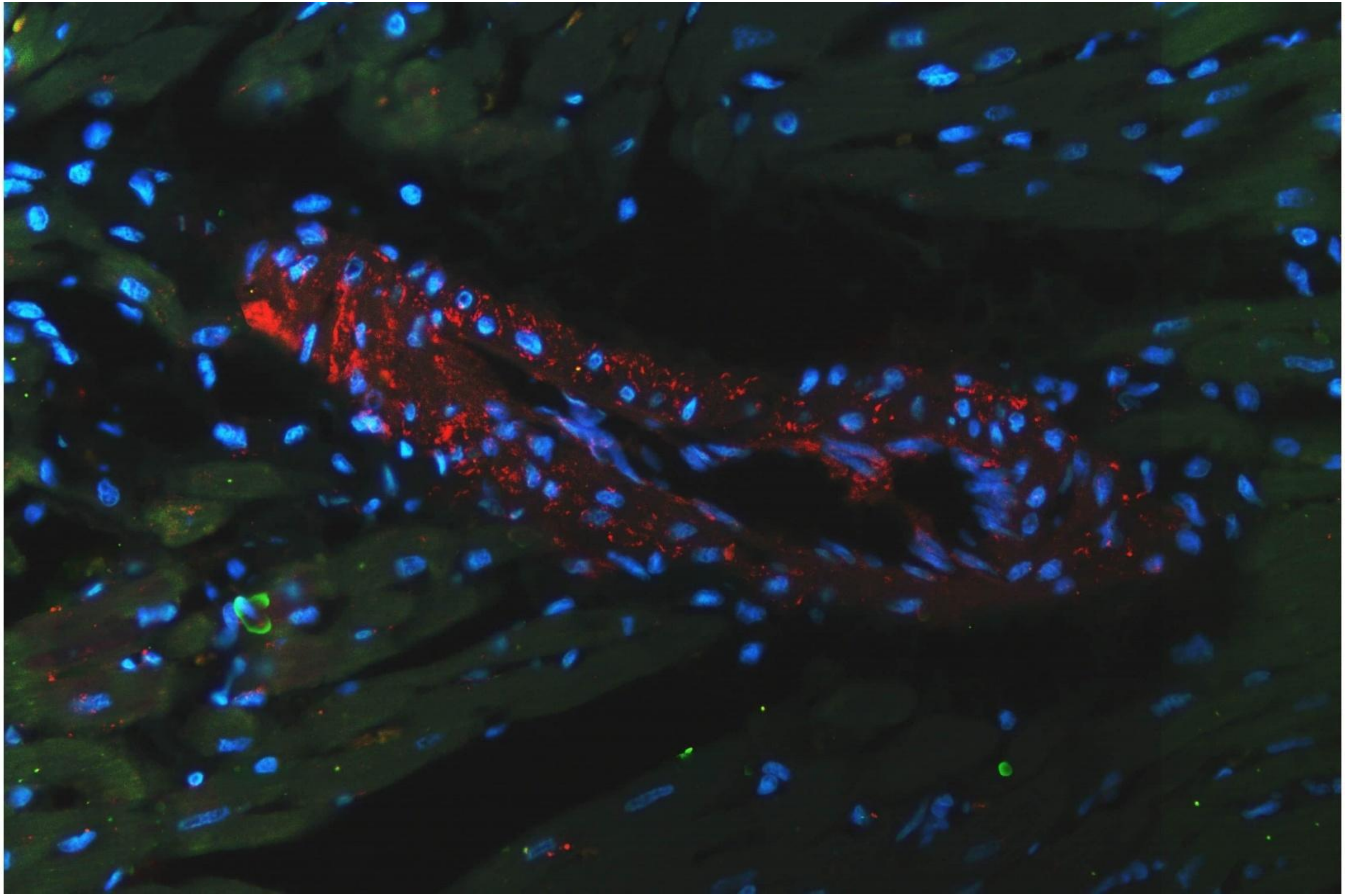

CIA+HFD+IR1

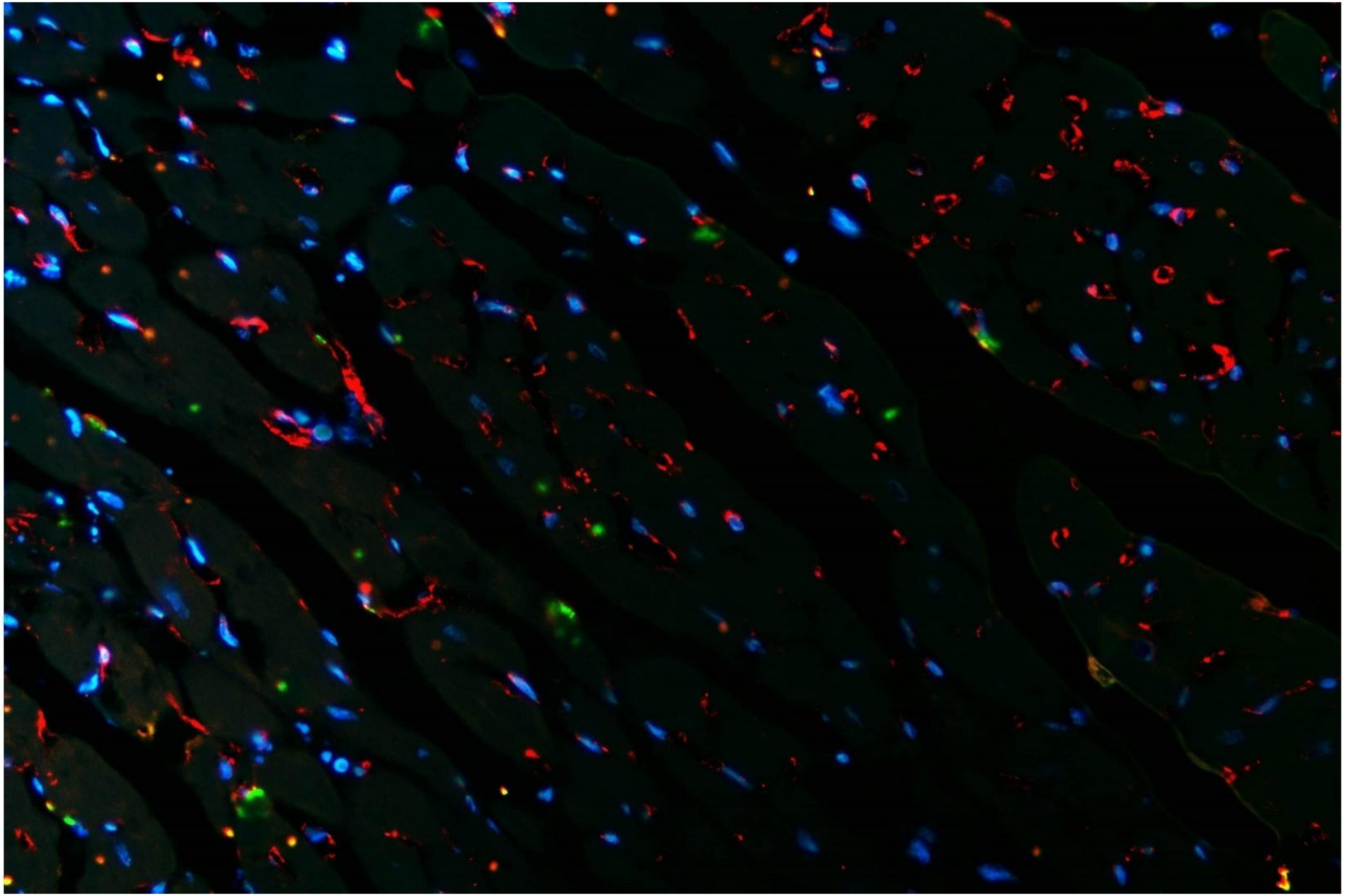

CIA3+HFD

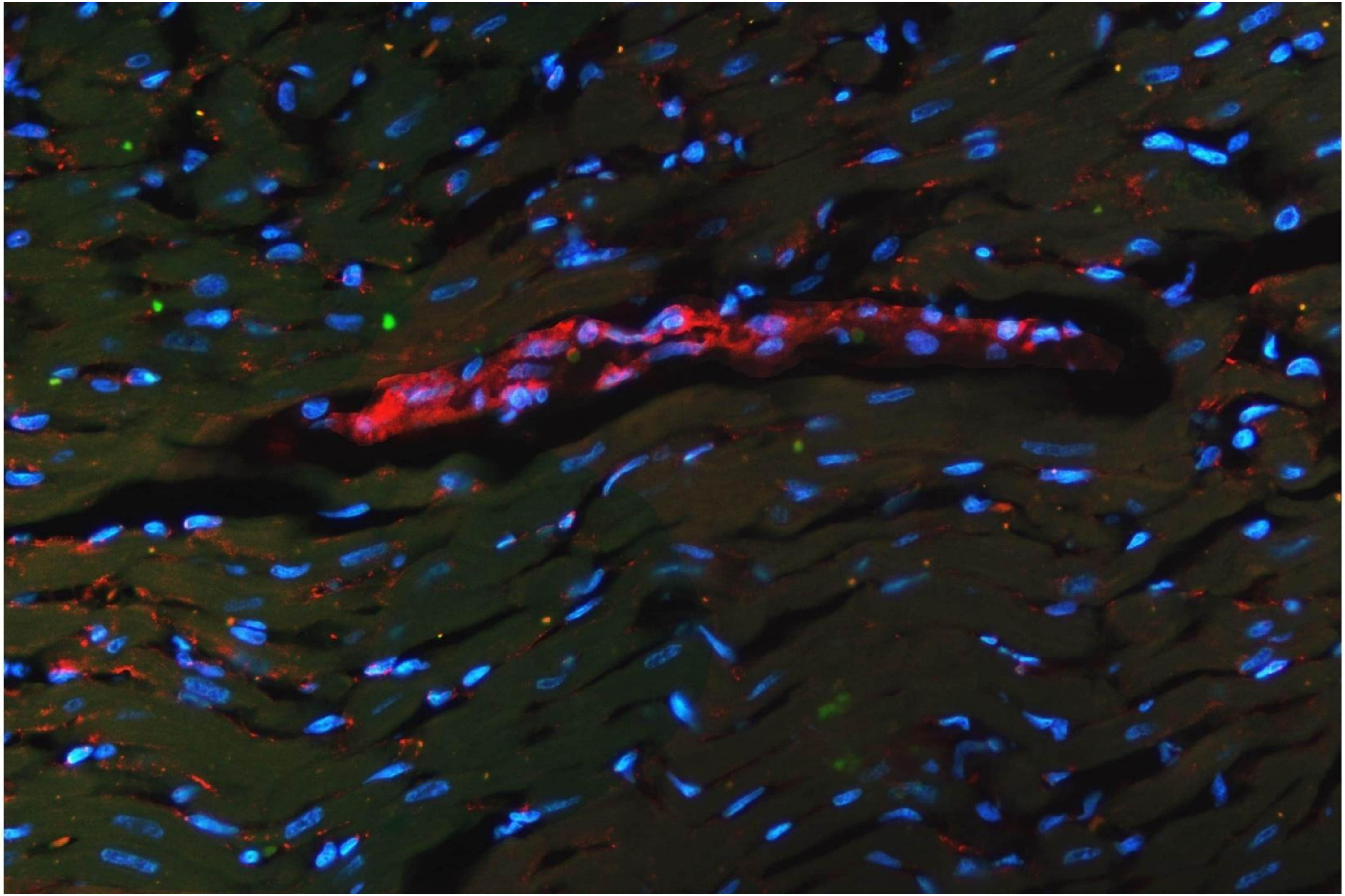

CIA3

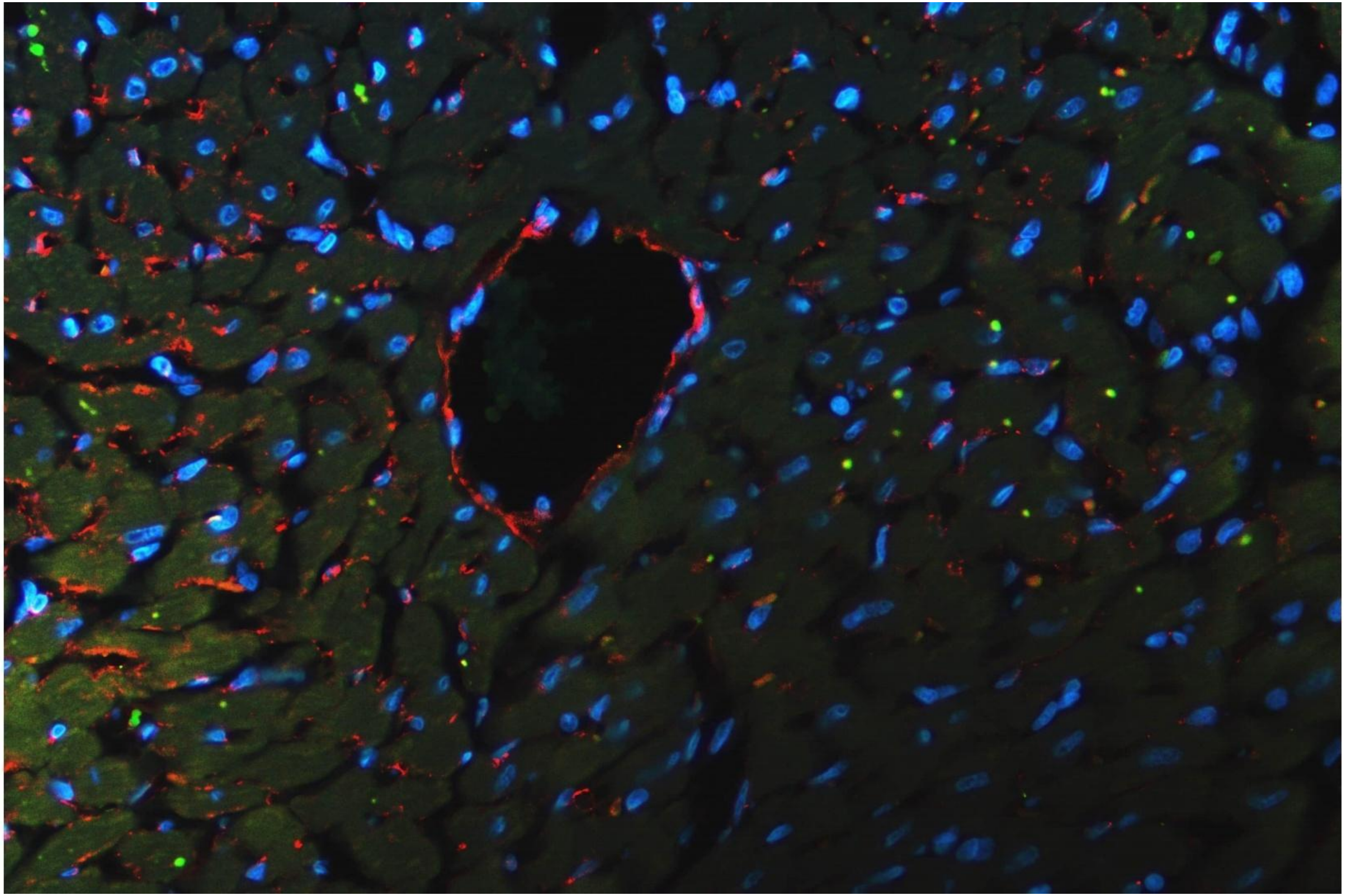

HFD1

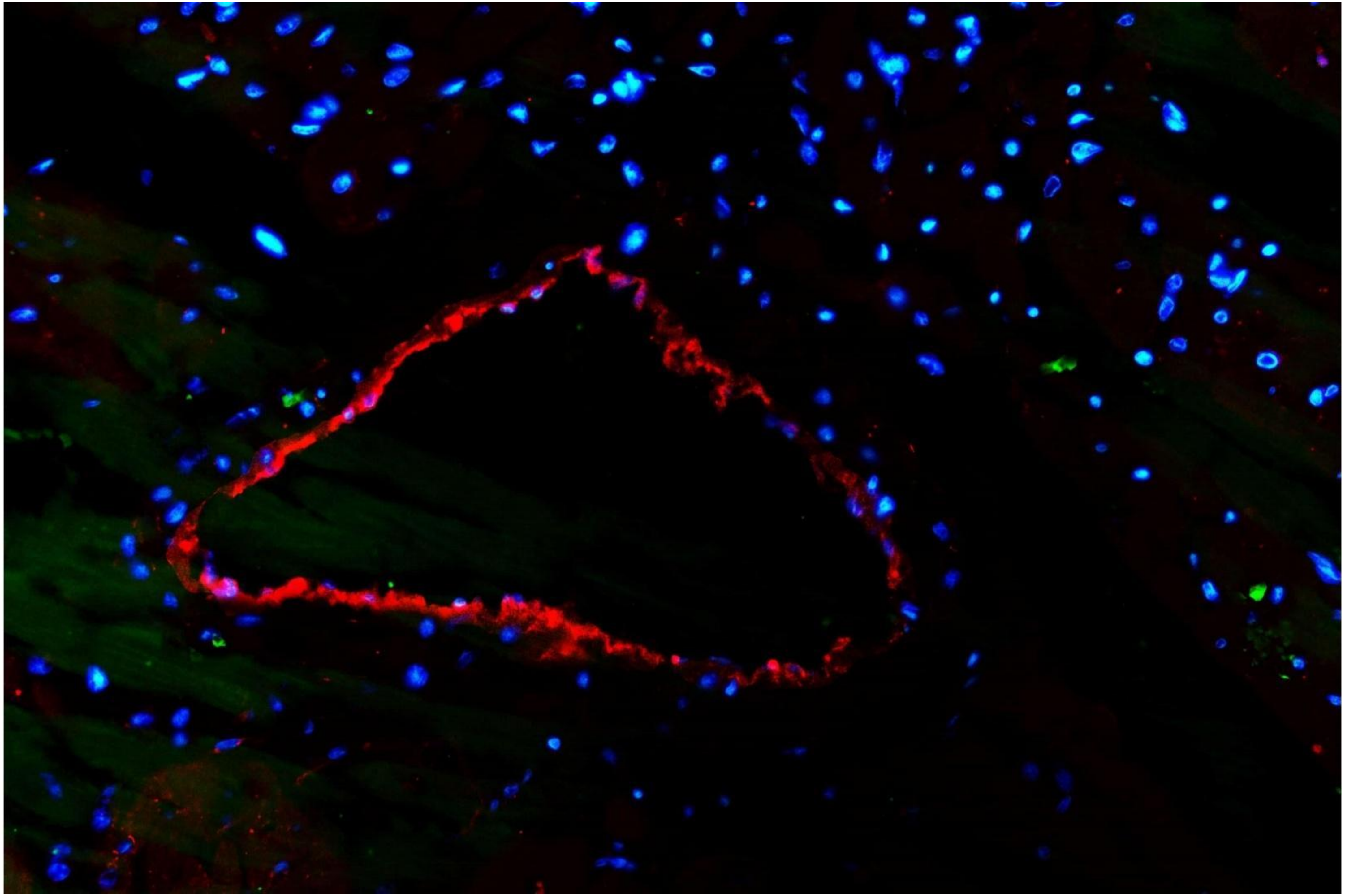

Normal

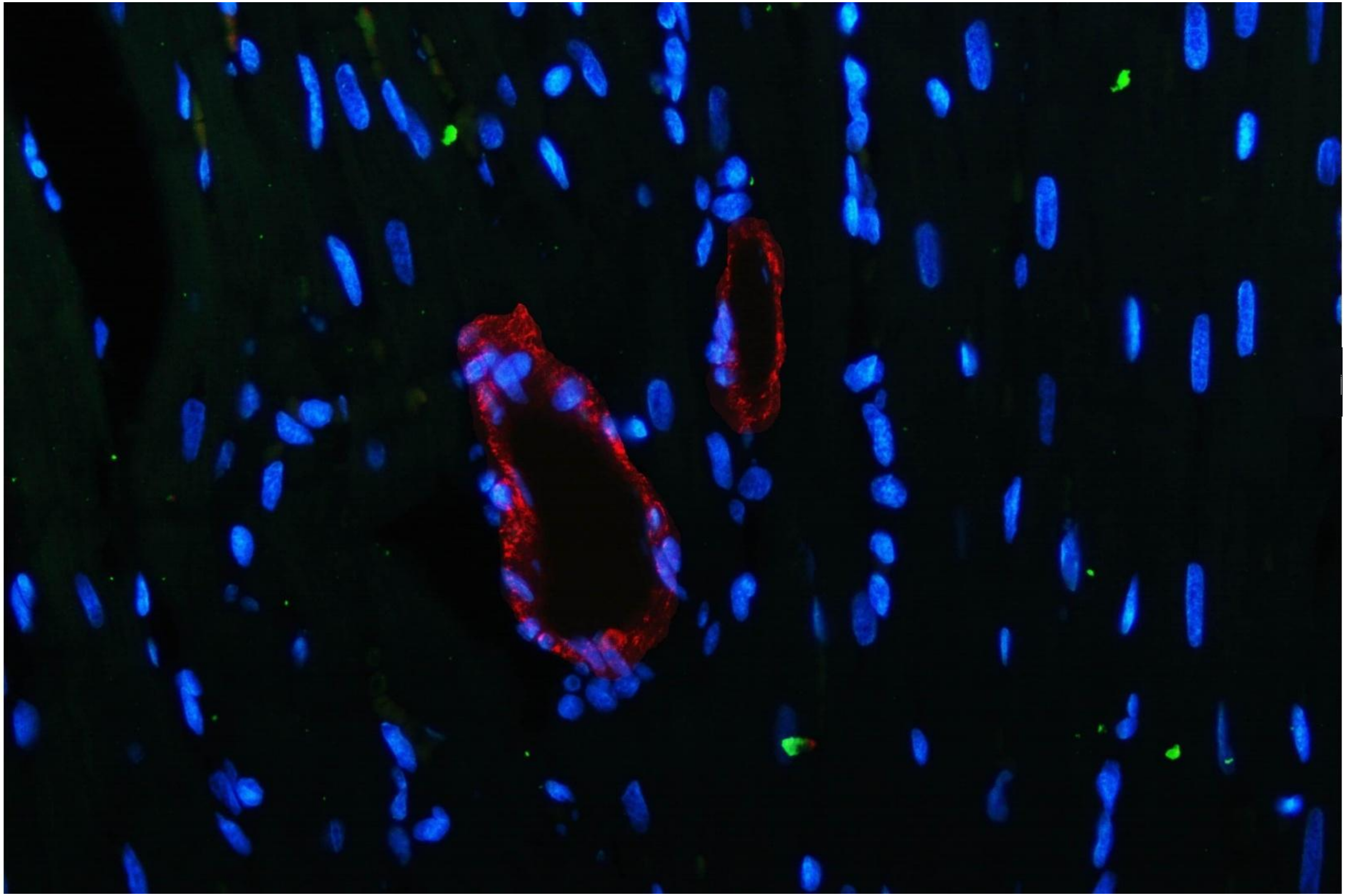

Figure 8b

Control+AAV-ANTI-PTRF

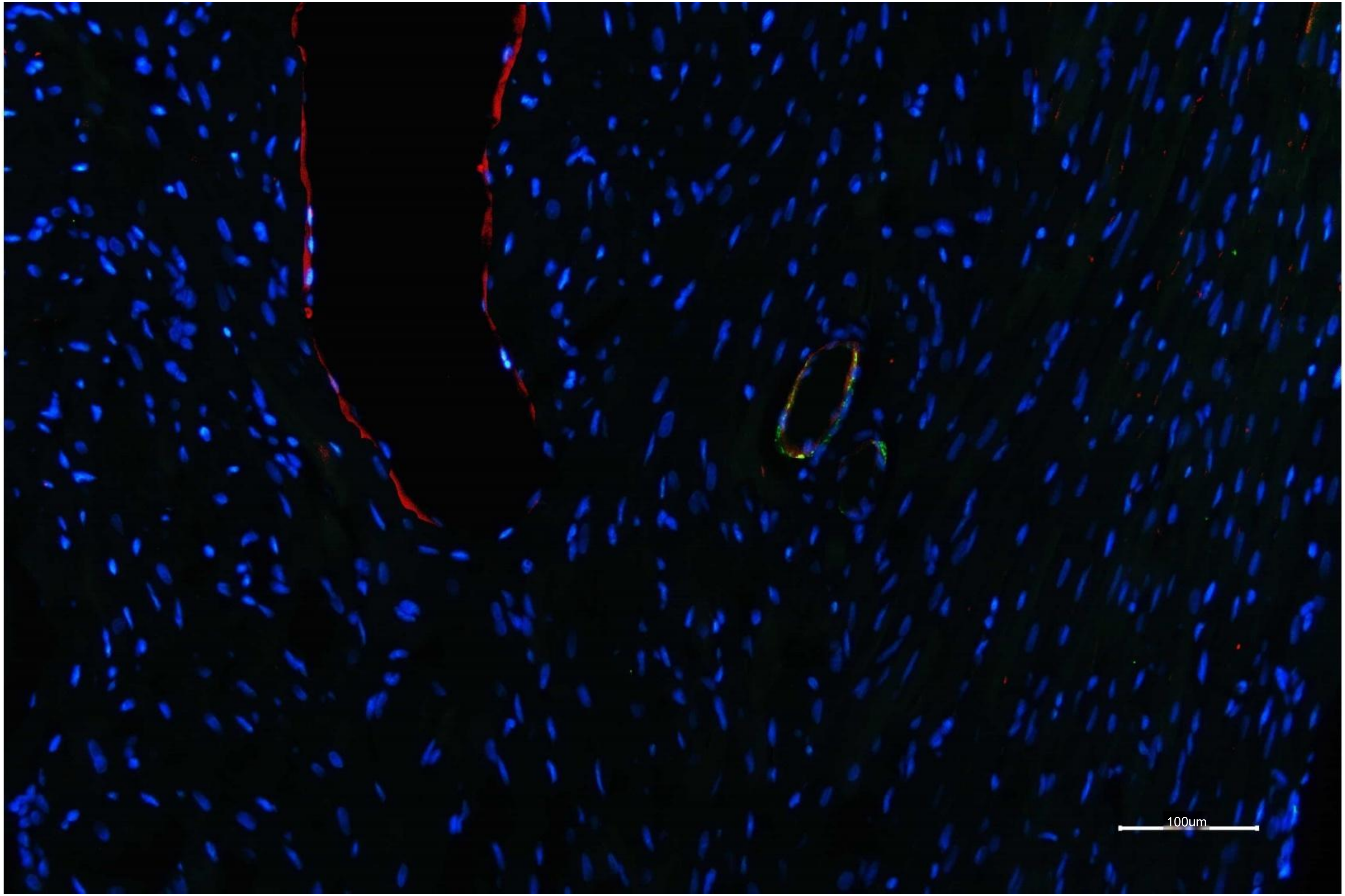

Control+AAV-NC

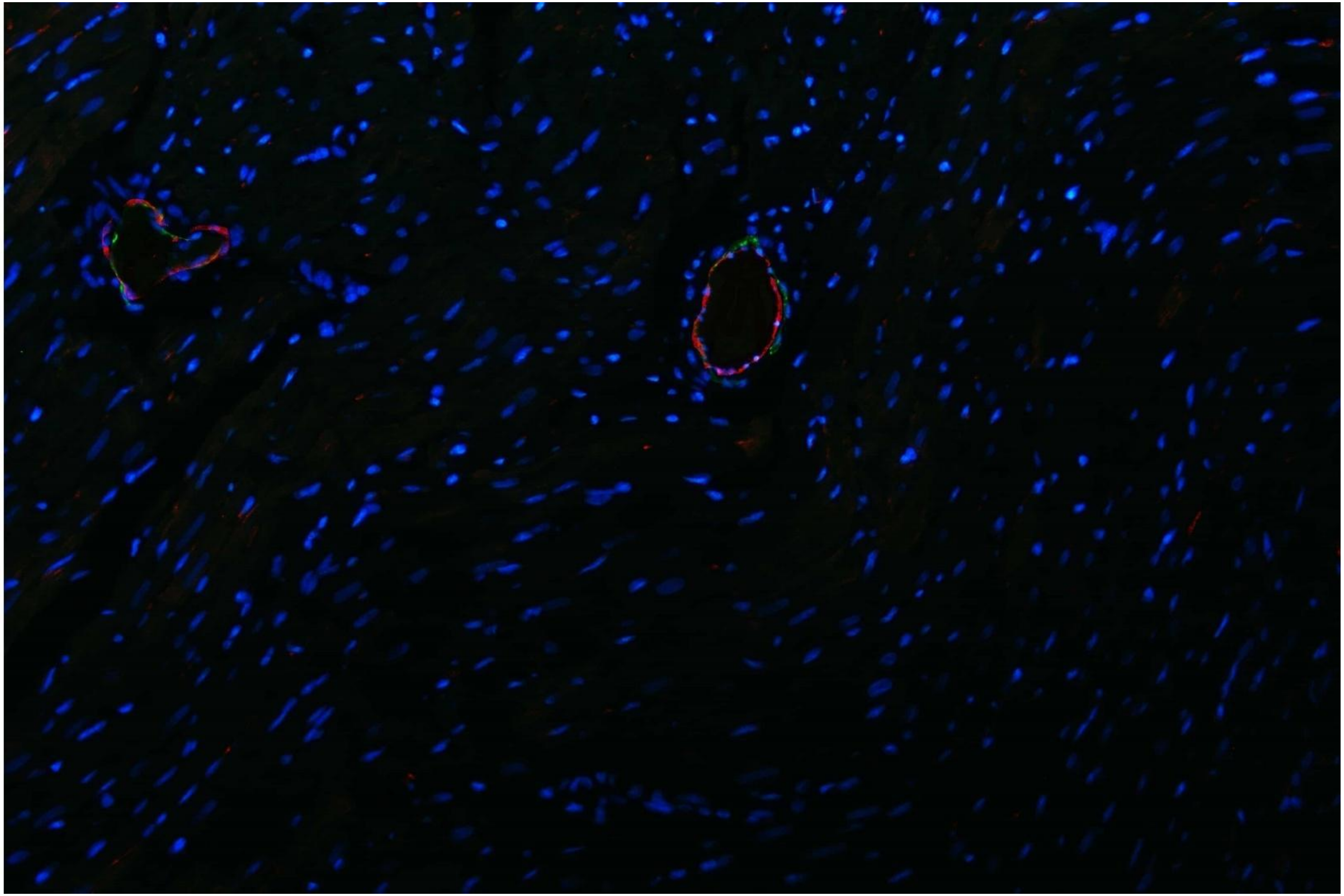

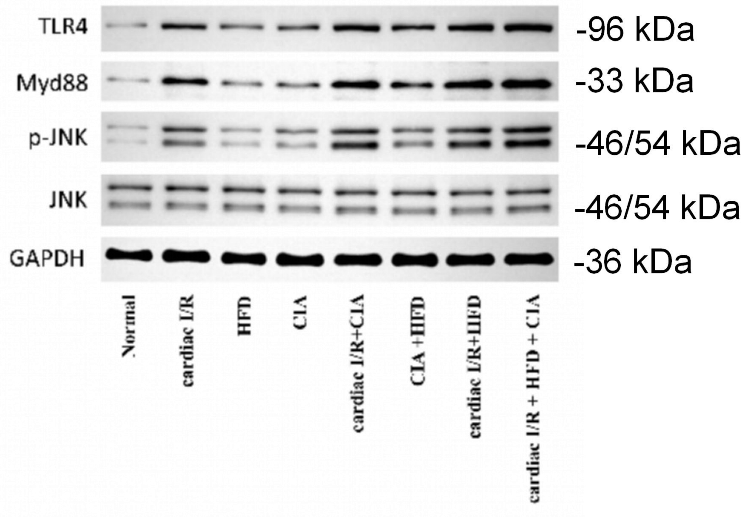

JNK

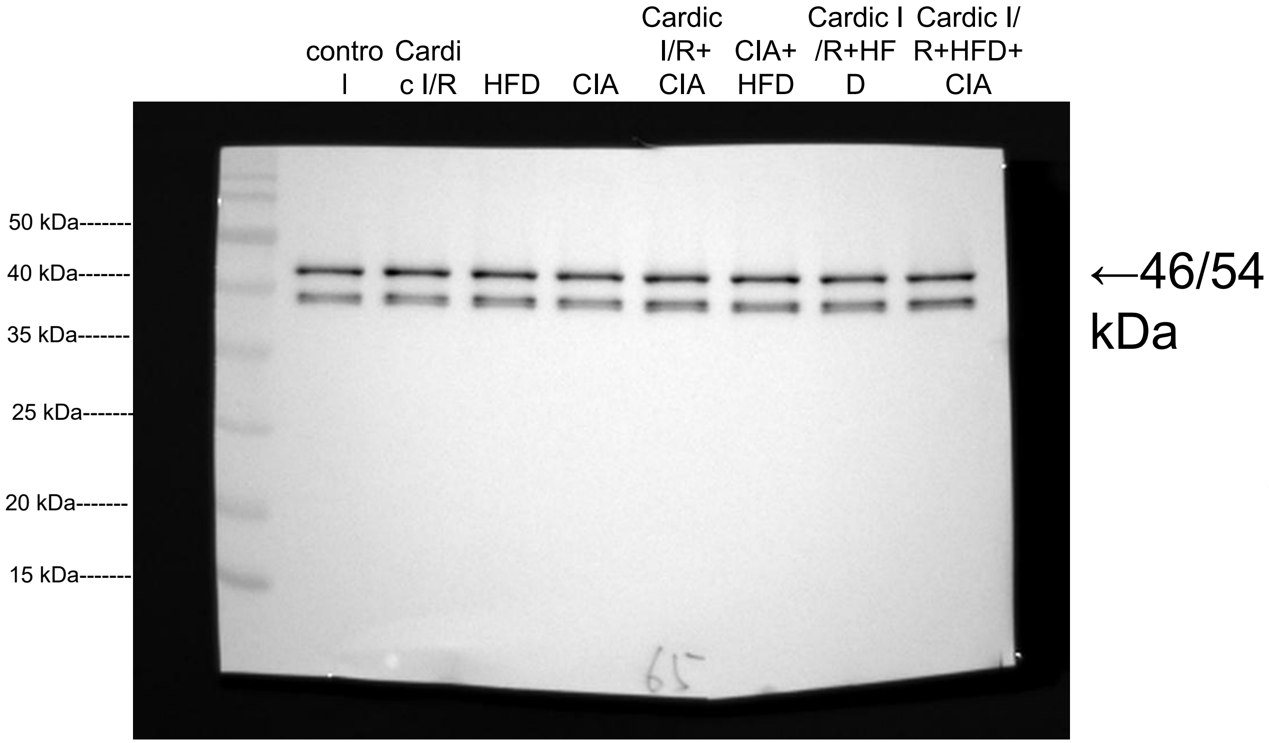

JNK-internal reference

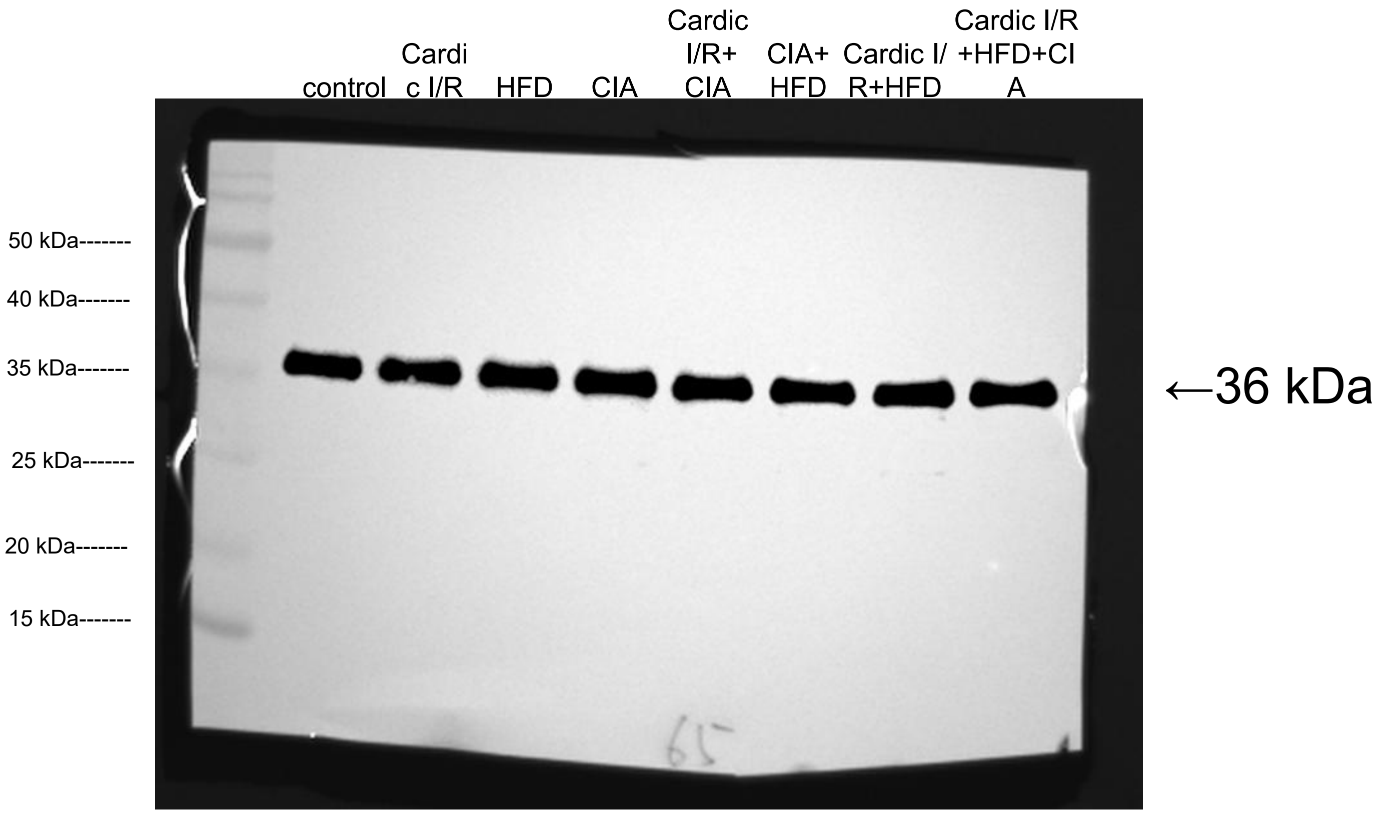

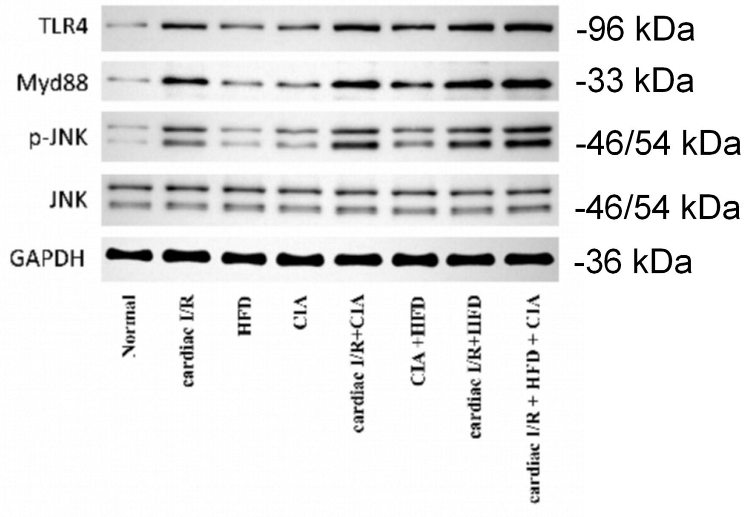

p-jnk

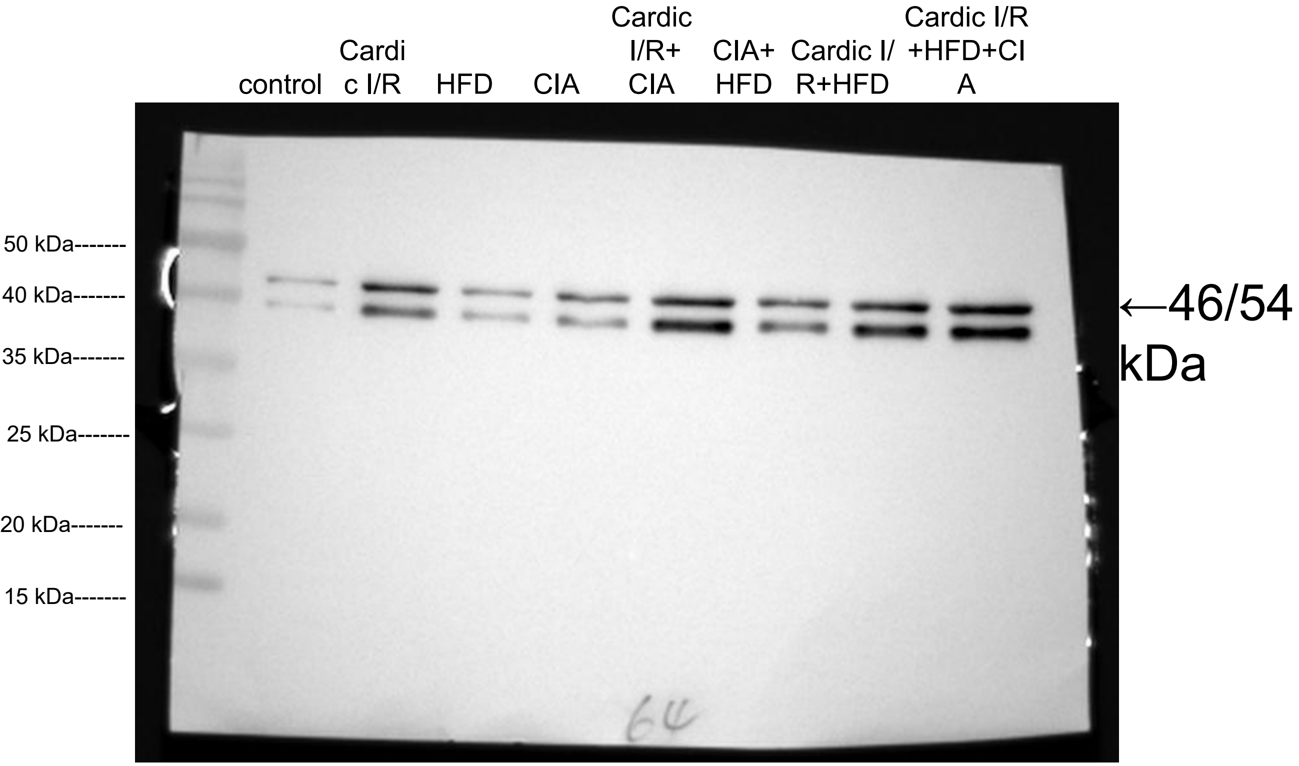

p-jnk-internal reference

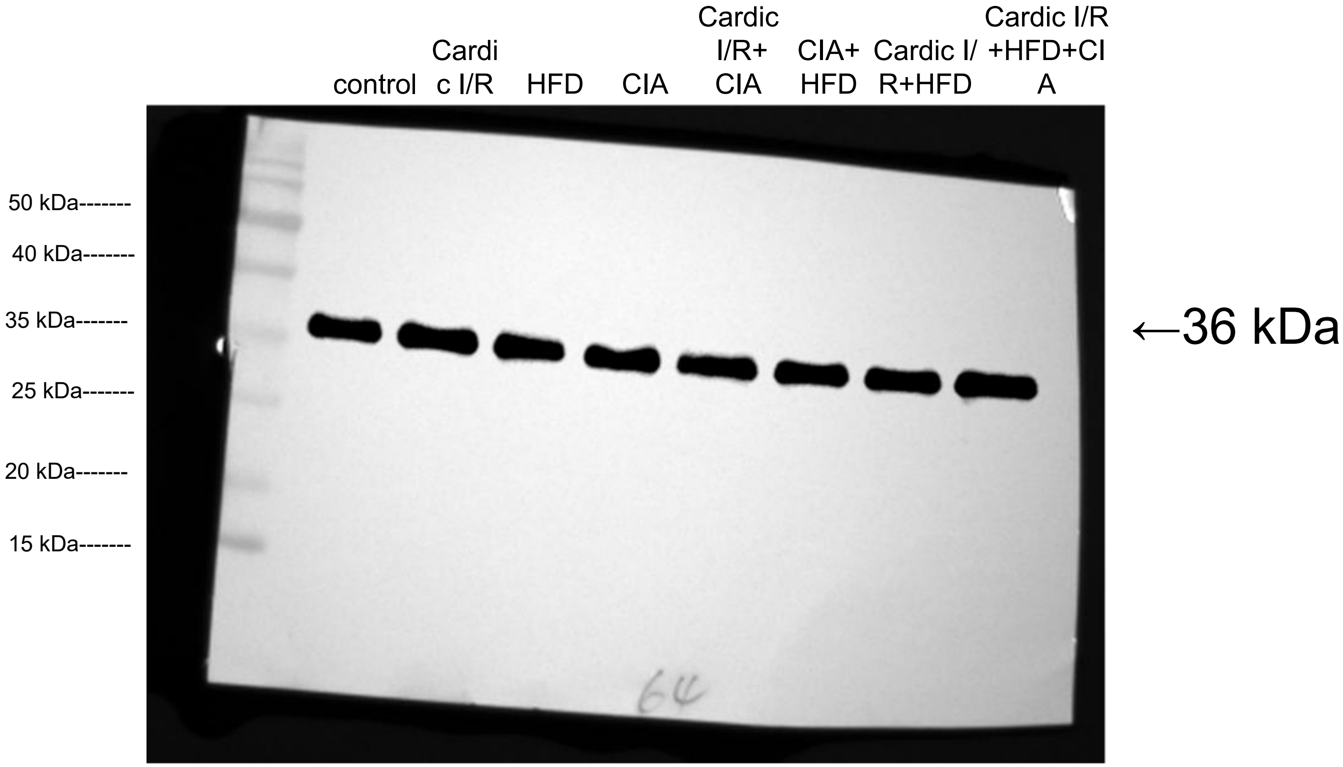

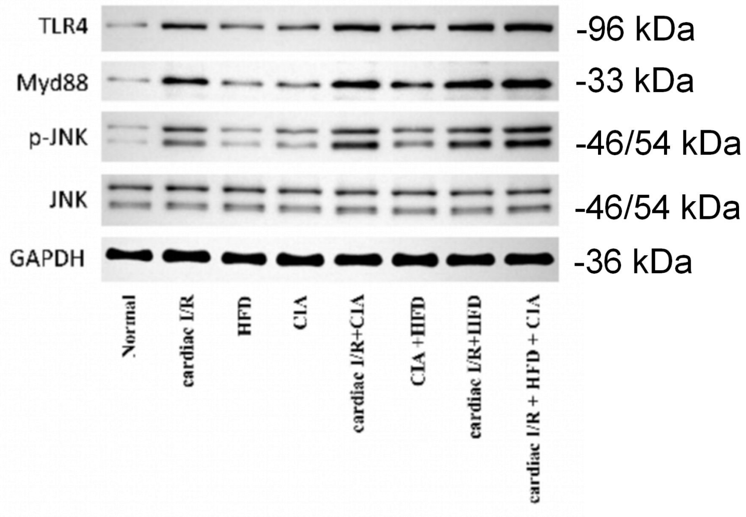

TLR4

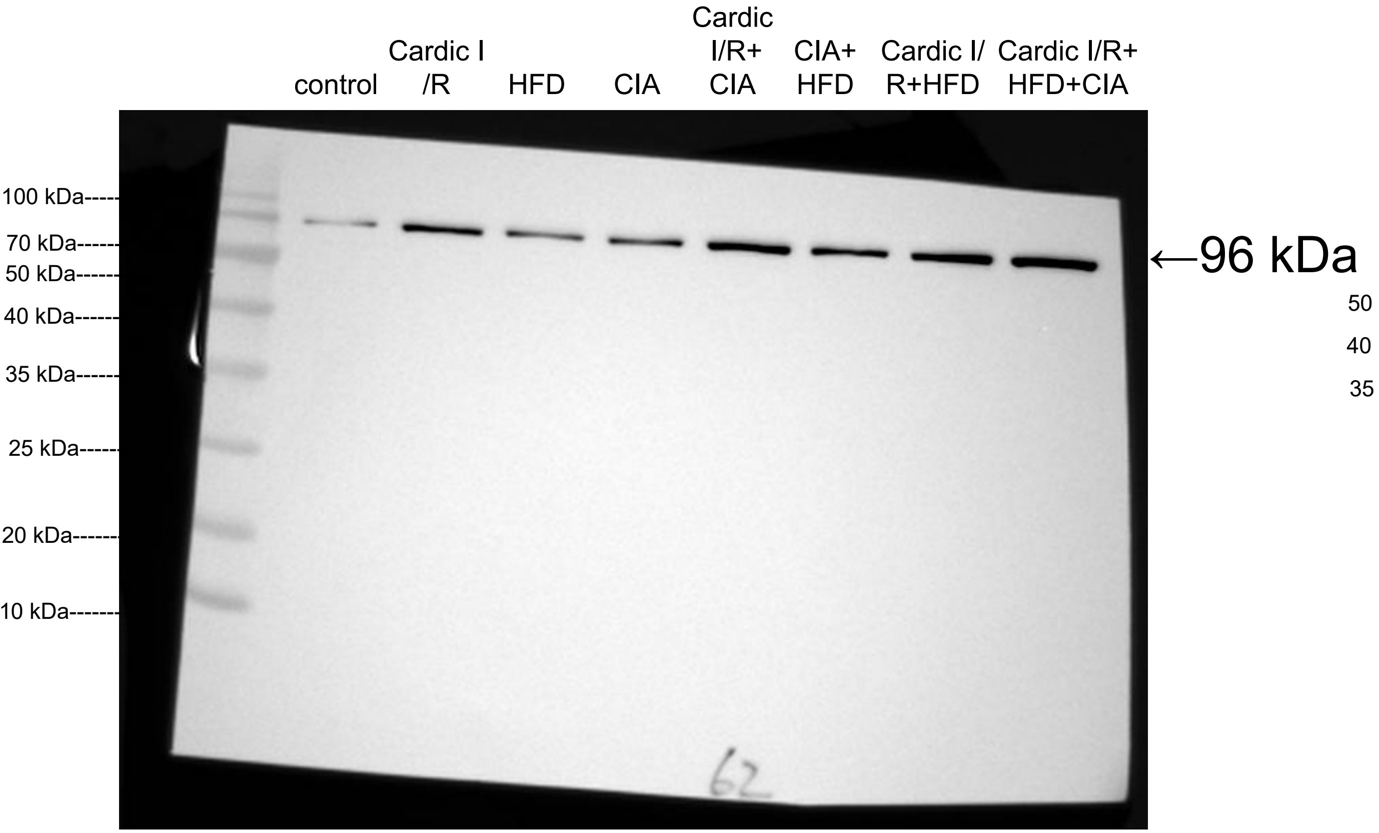

TLR4-internal reference

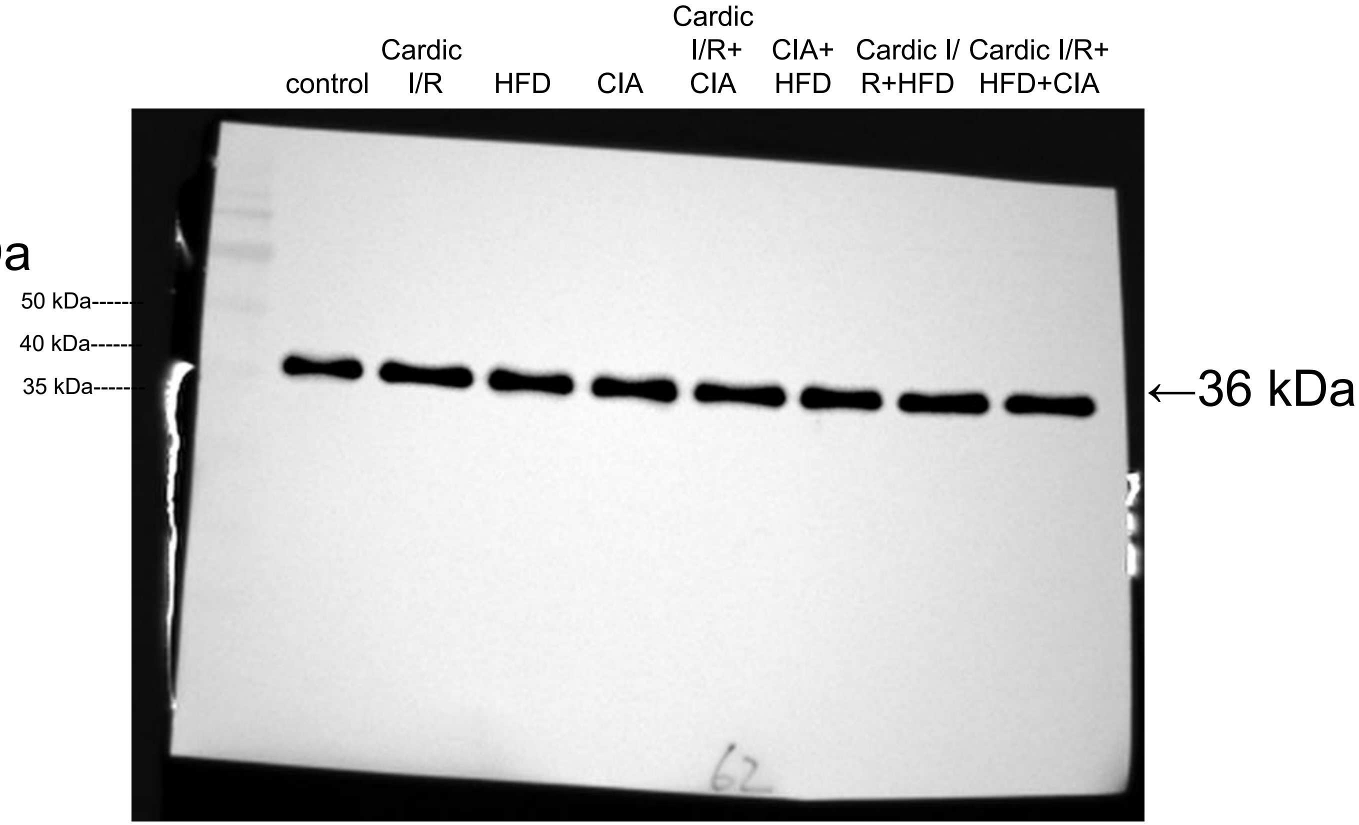

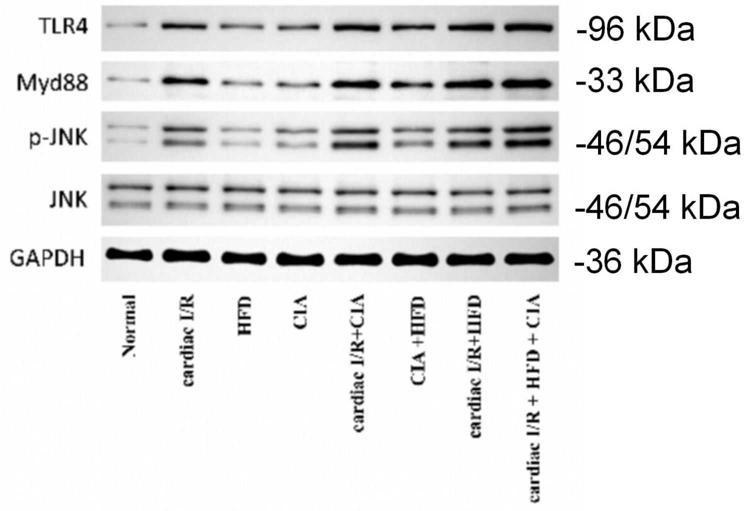

myd88

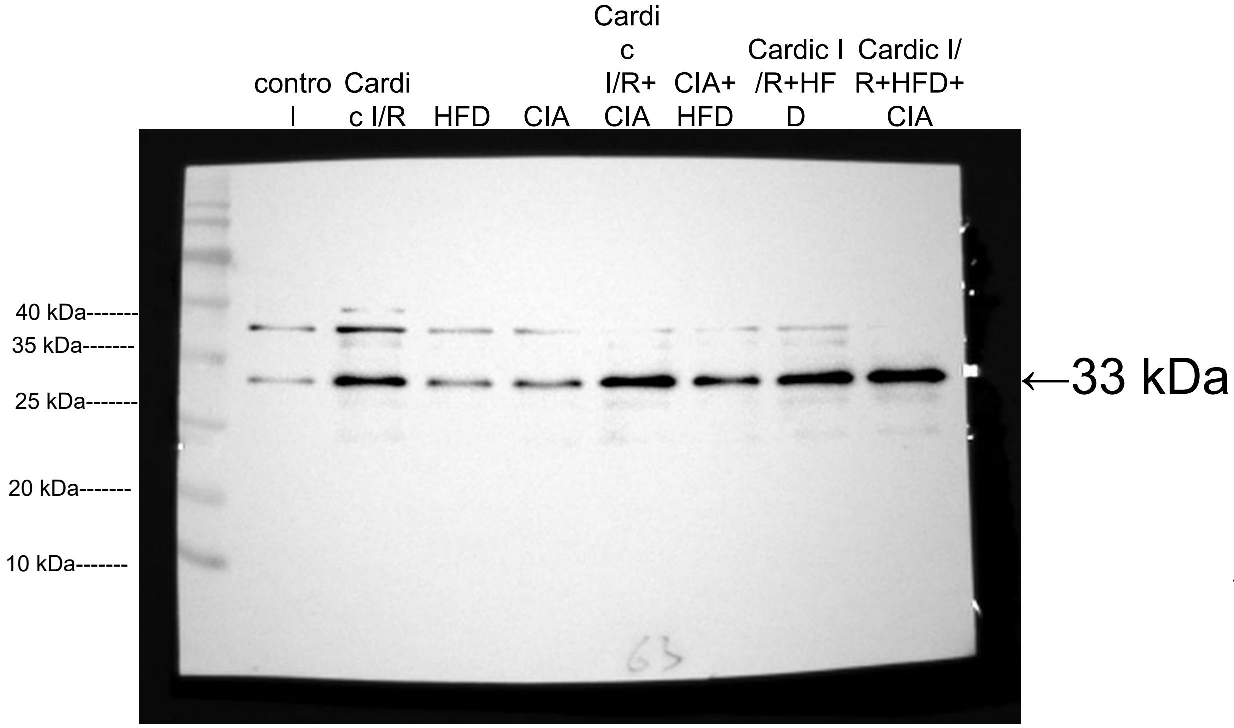

myd88-internal reference

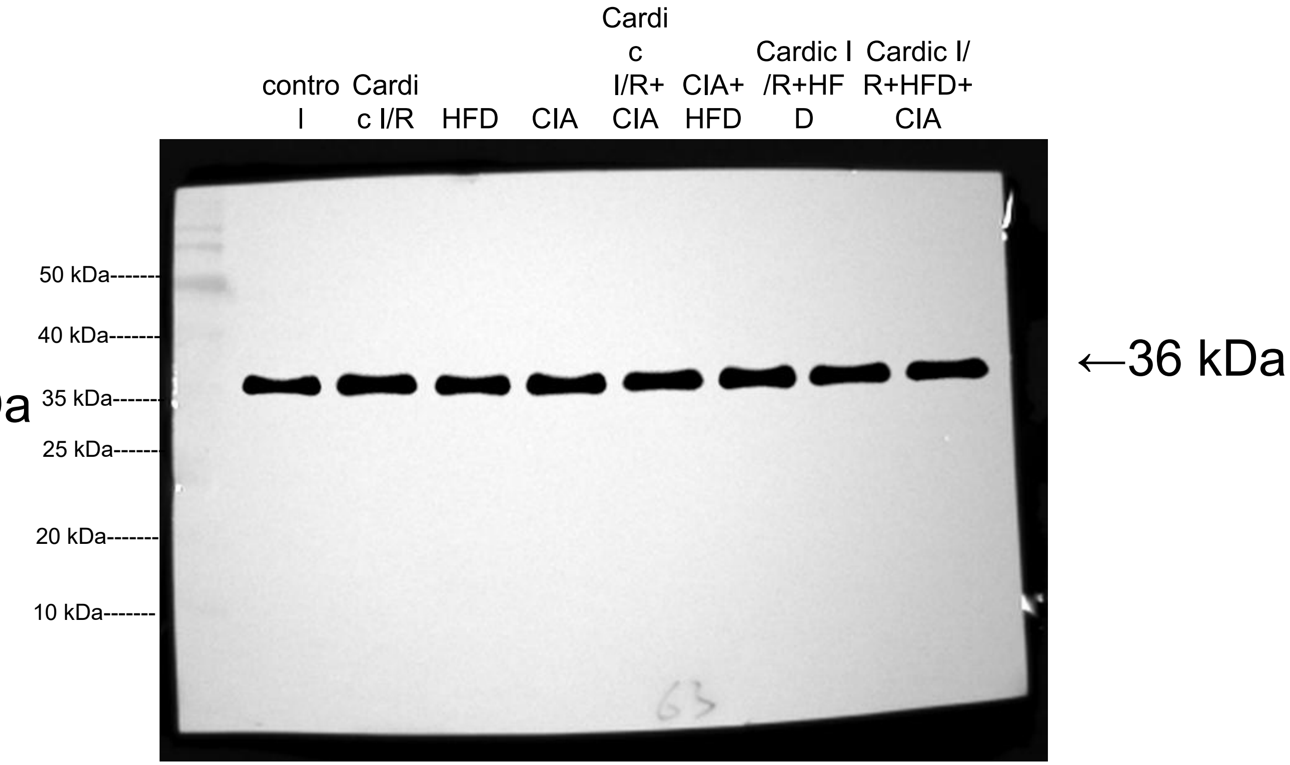

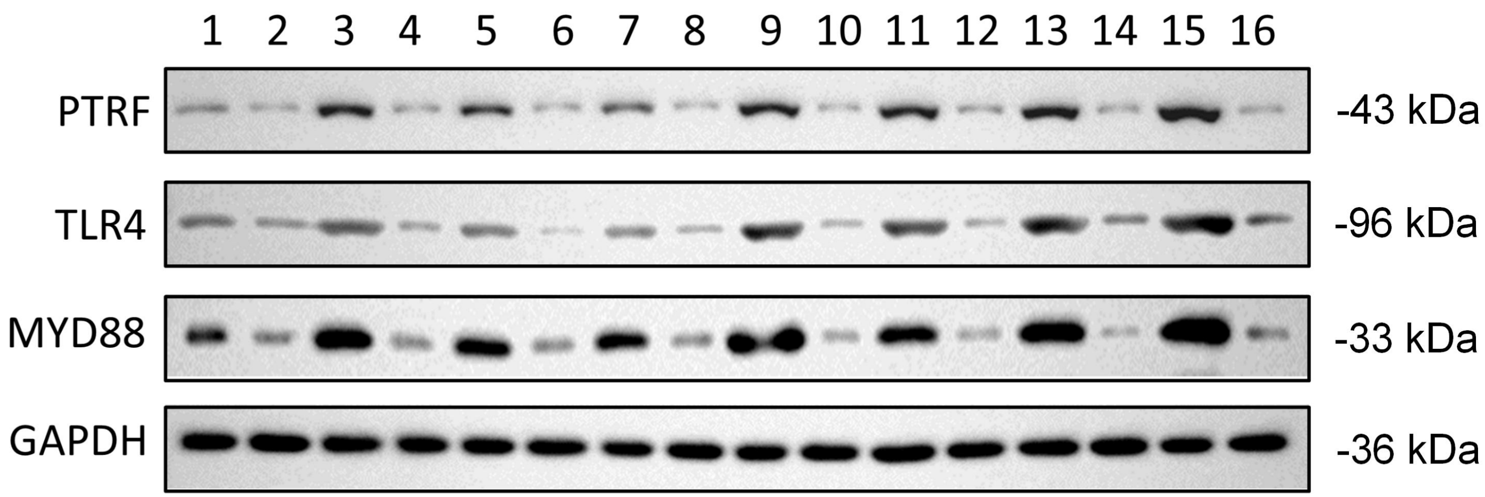
1 2 3 4 5 6 7 8 9 10 11 12 13 14 15 16
PTRF
-43 kDa
TLR4
-96 kDa
MYD88
-33 kDa
GAPDH
-36 kDa

PTRF

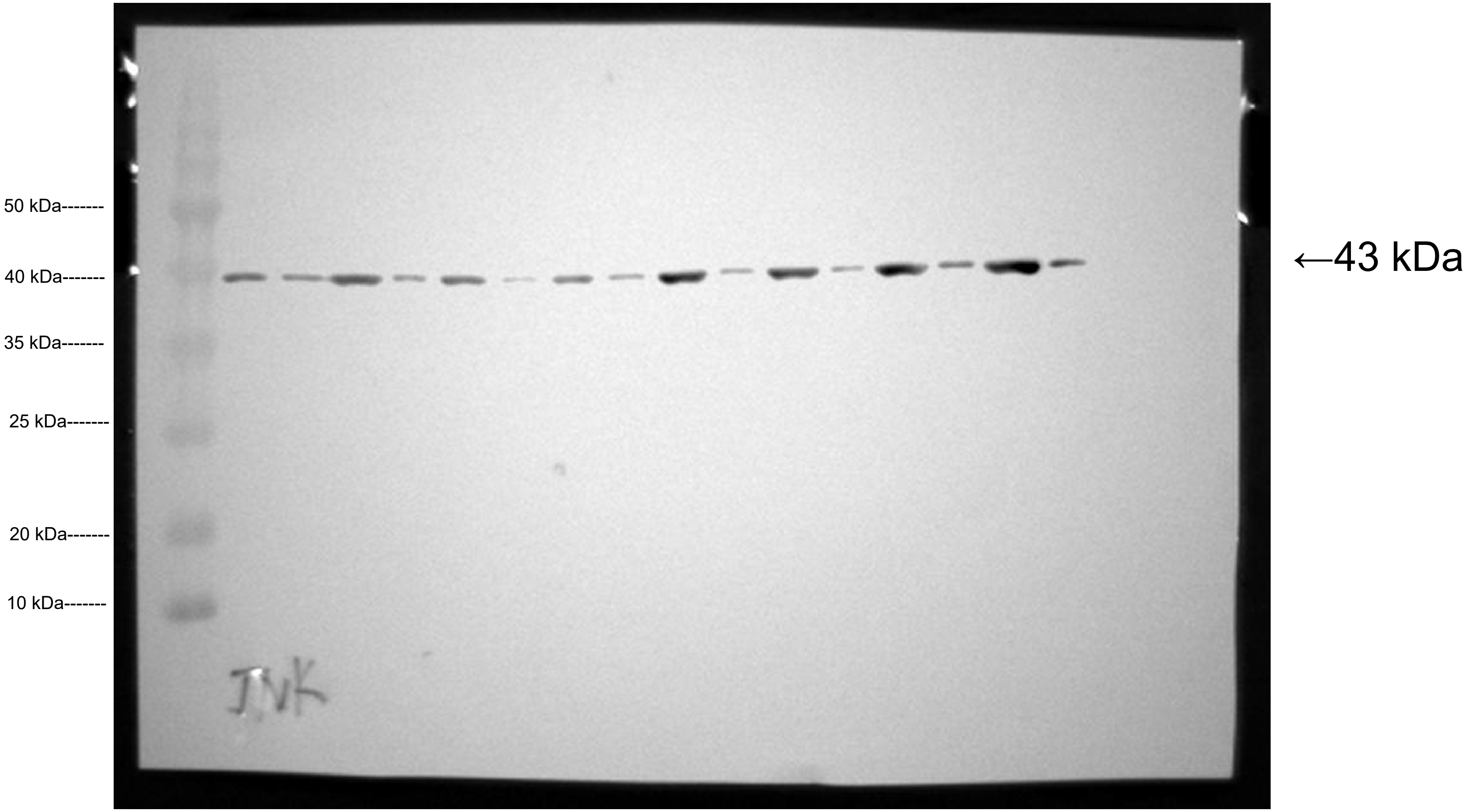
50 kDa-------
40 kDa-------
35 kDa-------
25 kDa-------
20 kDa-------
10 kDa-------
←43 kDa
JNK

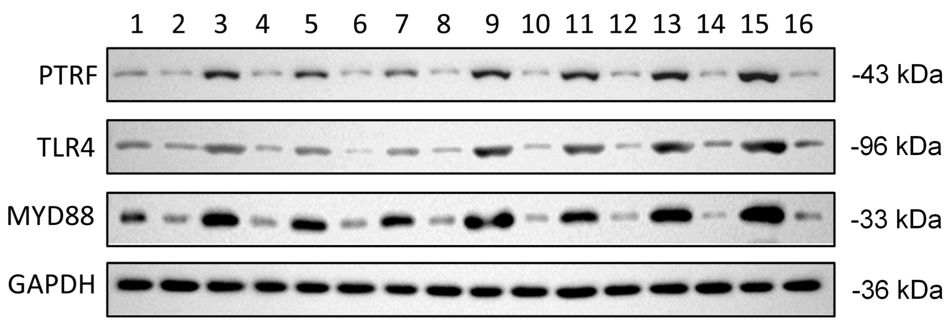
1 2 3 4 5 6 7 8 9 10 11 12 13 14 15 16
PTRF
-43 kDa
TLR4
-96 kDa
MYD88
-33 kDa
GAPDH
-36 kDa

TLR4

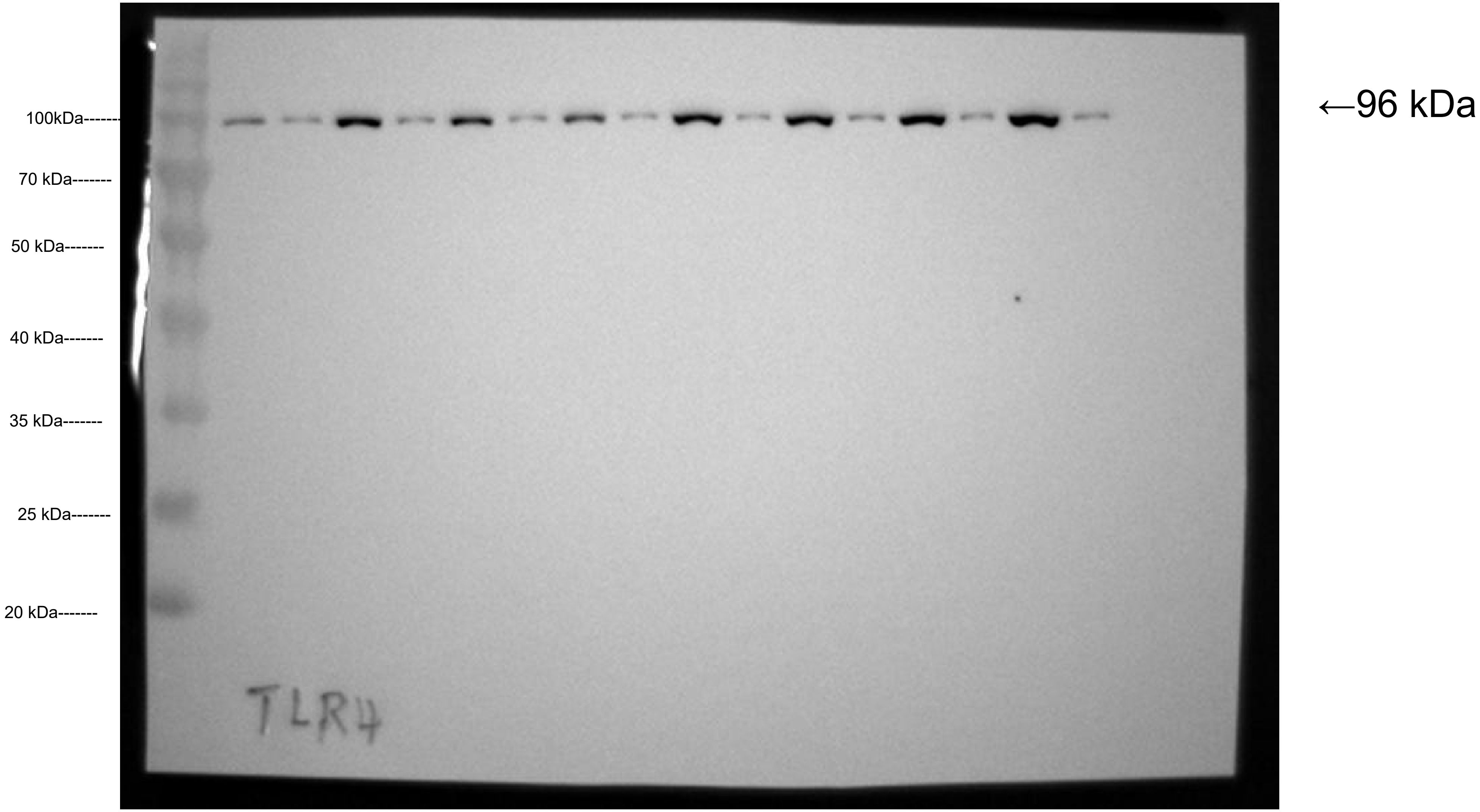
100kDa-------
70 kDa-------
50 kDa-------
40 kDa-------
35 kDa-------
25 kDa-------
20 kDa-------
TLR4
←96 kDa

MYD88

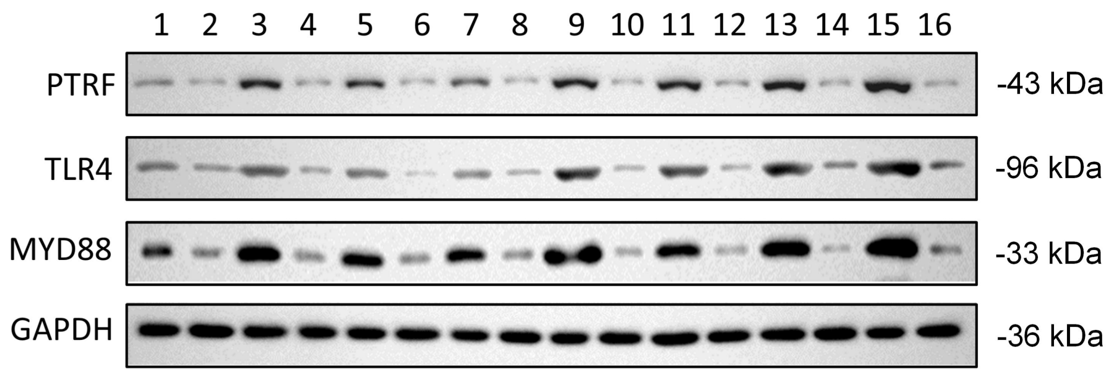

1 2 3 4 5 6 7 8 9 10 11 12 13 14 15 16
PTRF
TLR4
MYD88
GAPDH
-43 kDa
-96 kDa
-33 kDa
-36 kDa

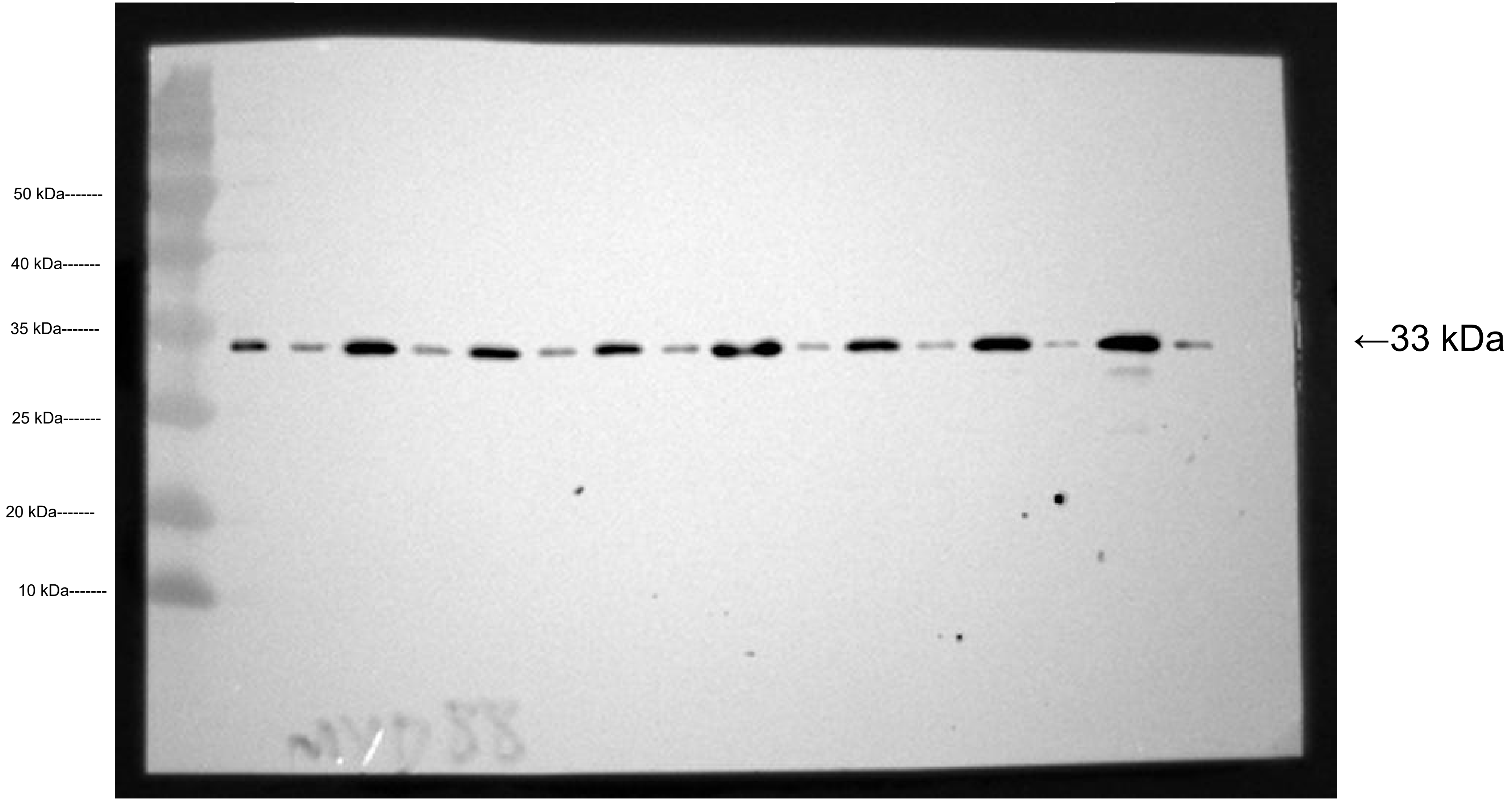

50 kDa-------
40 kDa-------
35 kDa-------
25 kDa-------
20 kDa-------
10 kDa-------
←33 kDa

GAPDH

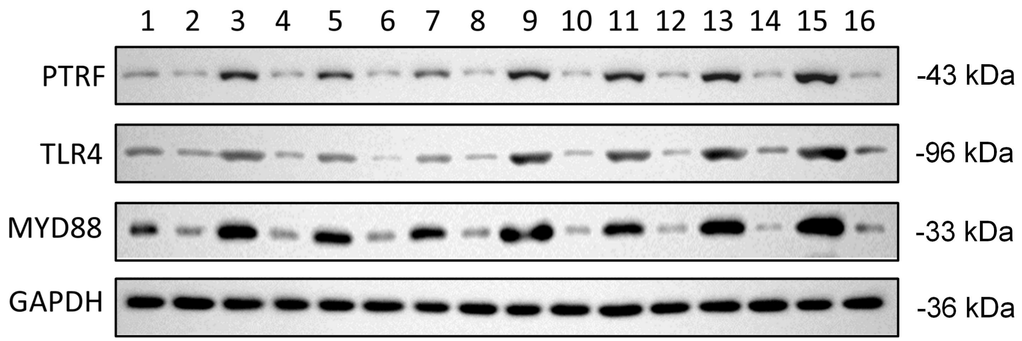
1 2 3 4 5 6 7 8 9 10 11 12 13 14 15 16
PTRF
-43 kDa
TLR4
-96 kDa
MYD88
-33 kDa
GAPDH
-36 kDa

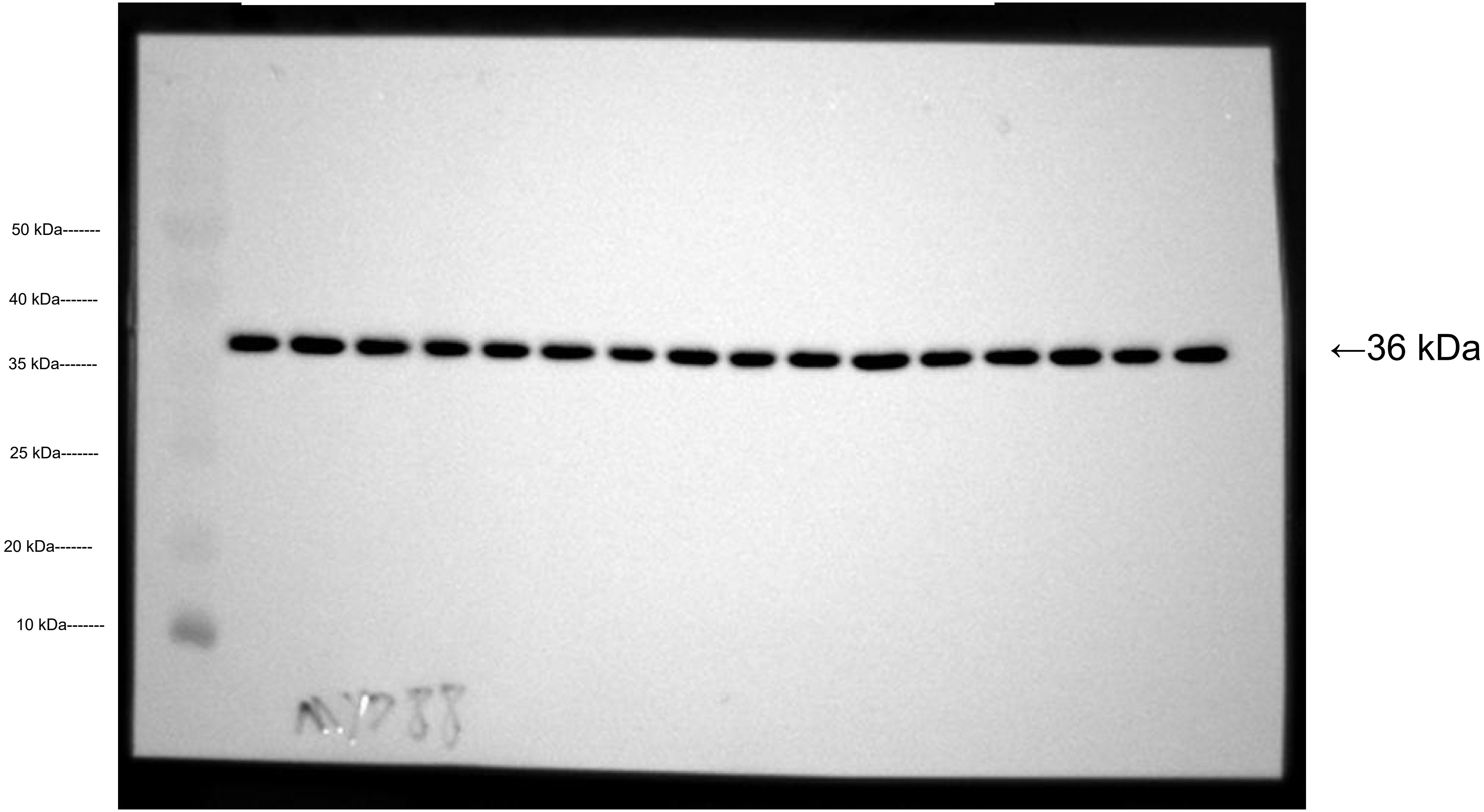
50 kDa-------
40 kDa-------
35 kDa-------
25 kDa-------
20 kDa-------
10 kDa-------
←36 kDa
